# Computational Peptide Design Cotargeting Glucagon and Glucagon-like Peptide-1 Receptors

**DOI:** 10.1021/acs.jcim.3c00752

**Published:** 2023-07-31

**Authors:** Shubham Vishnoi, Shayon Bhattacharya, Erica M. Walsh, Grace Ilevbare Okoh, Damien Thompson

**Affiliations:** † Department of Physics, Bernal Institute, University of Limerick, Limerick V94T9PX, Ireland; ‡ Merck, Kenilworth, New Jersey 07033, United States; § Merck, West Point, Pennsylvania 19486, United States

## Abstract

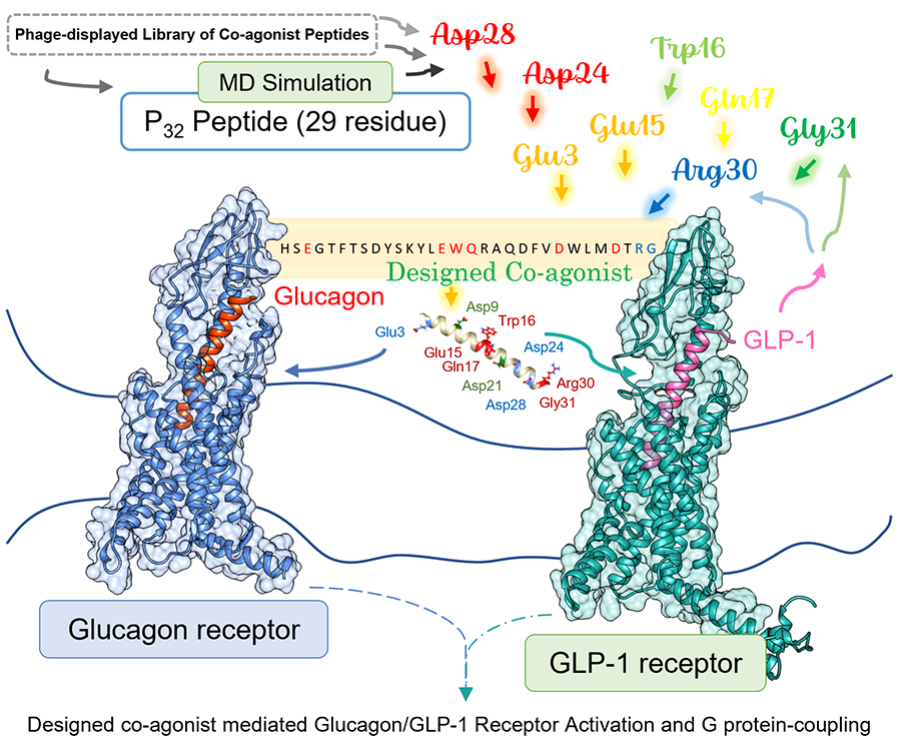

Peptides are sustainable alternatives to conventional therapeutics for G protein-coupled receptor (GPCR) linked disorders, promising biocompatible and tailorable next-generation therapeutics for metabolic disorders including type-2 diabetes, as agonists of the glucagon receptor (GCGR) and the glucagon-like peptide-1 receptor (GLP-1R). However, single agonist peptides activating GLP-1R to stimulate insulin secretion also suppress obesity-linked glucagon release. Hence, bioactive peptides cotargeting GCGR and GLP-1R may remediate the blood glucose and fatty acid metabolism imbalance, tackling both diabetes and obesity to supersede current monoagonist therapy. Here, we design and model optimized peptide sequences starting from peptide sequences derived from earlier phage-displayed library screening, identifying those with predicted molecular binding profiles for dual agonism of GCGR and GLP-1R. We derive design rules from extensive molecular dynamics simulations based on peptide–receptor binding. Our newly designed coagonist peptide exhibits improved predicted coupled binding affinity for GCGR and GLP-1R relative to endogenous ligands and could in the future be tested experimentally, which may provide superior glycemic and weight loss control.

## 1 Introduction

G protein-coupled receptors (GPCRs) are the largest family of transmembrane receptors, regulating the biological processes of growth, metabolism, and homeostasis. Currently, GPCRs are targeted by over 700 approved active pharmaceutical ingredients (APIs).^1−3^ Among these, the glucagon (GCG) and glucagon-like peptide-1 (GLP-1) receptors are targets for the treatment of type-2 *diabetes mellitus* (T2DM) and high cholesterol levels in obesity.^4−6^ Bioactive peptides derived from natural sources have proven efficacies^7,8^ with more than 50 GPCR-targeting peptide APIs approved to date.^9^ Twenty of these APIs target class B1 GPCRs with more than 30 peptide therapeutics currently in the drug development pipeline.^9−11^ Peptide APIs for GPCR targeting may function as receptor agonists stimulating GPCRs to activate intracellular signaling and trigger biological response or as antagonists.

The GCG and GLP-1 receptors (both belonging to the glucagon receptor subfamily, hence collectively termed GRs here) belong to the class B1 GPCR which includes receptors for peptide hormones such as glucagon, GLP-1, glucose-dependent insulinotropic polypeptide (GIP), and secretin. Class B1 GPCRs are activated by ligands, such as small molecules or peptides that bind to the extracellular domain (ECD) of the receptor. This binding triggers conformational changes in the transmembrane region (TMR) of the receptor, such as unravelling of the transmembrane α-helix 6 (TM6) at the highly conserved PxxG motif and disruption of the HETx network.^12^ These changes lead to the unwinding of the intracellular end of TM6 and moving outward.^10^ This promotes the recruitment following activation of heterotrimeric G protein consisting of three distinct subunits (Gα, Gβ, and Gγ) on the intracellular side of the membrane. The activated G protein then goes on to facilitate downstream signaling pathways.^13,14^ The GLP-1 and GCG endogenous peptides play a central role in regulating blood glucose levels, but marketed peptide APIs such as Semaglutide, Exenatide, and Liraglutide are only efficacious as a single agonist (monoagonist) of the GLP-1R for treatment of T2DM.^15^ Moreover, Liraglutide is prone to peptide aggregation at its injection site^16,17^ leading to undesired side effects. Both GCG and GLP-1 receptors are implicated in the pathophysiology of diabetes and fatty liver disease.^18^ Yet there is no proven pharmacotherapy currently available for the treatment of fatty liver disease. Since treatment of obesity and obesity-induced diabetes has failed with monoagonist therapy,^19−21^ the major treatment options remain lifestyle changes and body weight management. Despite the proven benefits of insulin and oral hypoglycemics such as metformin and troglitazone, there is a progressive deterioration of glycemic control in T2DM.^19,22^

In addition to GLP-1R agonists, novel unimolecular peptide dual agonists have been developed to activate both GLP-1R and GCGR and are currently under clinical trials.^23^ These peptides aim to elicit superior therapeutic effects compared to monoagonist peptides.^24^ The recent FDA approval of Tirzepatide, a GIP/GLP-1 receptor coagonist, marks a significant milestone in developing dual-receptor agonists for the treatment of T2DM^25,26^ with promising results to date.^27^ Despite these benefits, the molecular level mechanism of dual agonism remains unclear, so it is difficult to draw new design rules for other coagonists targeting GCGR/GLP-1R. In addition, the adverse interaction of Tirzepatide with alcohol and the necessity for prolonged treatment highlight the need for the design and development of more accessible and more equitable peptide-based therapy.^28^

Molecular modeling and extensive data mining indicate that GPCR agonism is achieved through local positional interactions and conformational shifts via dynamic loops.^29^ Hence, the characterization of solution structures and prediction of peptide-coupled binding affinity for GRs under physiological conditions are crucial to account for the dual activity of peptides with receptors. GRs carry a large ECD at the N-terminal domain that hosts the ligand binding pocket. The flexibility of the ECD coupled with the structured binding pocket facilitates entry and recognition of peptide agonists (see Figure S1, Supporting Information). The structural domain encompassing the TMR of both GCGR and GLP-1R is highly conserved (Figure S2A), but the primary sequences of extra- and intracellular loop regions are more diverse (Figure S2B). Cryogenic electron microscopy (cryo-EM) maps confirm the critical role of extracellular loop 2 (ECL2) in the recognition of native peptide endogenous agonists.^13,14^

The N-terminal region (NTR) of the native agonists GCG and GLP-1 of GCGR and GLP-1R shares a conserved primary sequence with oxyntomodulin (OXM), a natural dual agonist 37-residue peptide gut hormone that suppresses appetite.^30,31^ The experimental drug Cotadutide mimics this satiating effect by modulating the hepatic glycogen and fat content and is currently in phase-IIb/III clinical trials as a dual receptor peptide agonist with balanced GCG/GLP-1 activity.^26,32^ A recent study demonstrates that Cotadutide stimulates GCGR to reduce hepatic glycogen and fat accumulation in the liver,^33^ and studies of diet-induced obese mice demonstrate that Cotadutide improves insulin sensitivity and restores normal insulin secretion.^32^ In human trials, Cotadutide has been demonstrated to effectively reduce blood glucose level and body weight in treating fatty liver disease in patients with T2DM.^32,34^ Despite the demonstrated promise of Cotadutide with reduced adverse effects,^35^ only monoagonist peptides targeting either GCGR or GLP-1R^36^ – but no coagonists targeting both – have been approved by the U.S. Food and Drug Administration to date. Beyond the approval of Tirzepatide cotargeting GLP-1R and GIPR,^37^ several unimolecular peptide-based coagonists are under clinical trials at the time of writing in April 2023, highlighting the clinical significance of the dual-agonist therapy for the development of bioinspired APIs for metabolic and hormonal disorders.^38,39^

Motivated by recent experimental phage-displayed library (PDL) screens of bioactive peptides that simultaneously agonize GLP-1R and GCGR,^40^ we systematically engineered peptide sequences of chimeric analogues of the endogenous peptide ligands to computationally design new peptide APIs with promising coagonist binding profiles. We benchmarked the predicted agonistic effect against the extensive PDL together with the reference endogenous peptide ligands and the experimental drug, Cotadutide. In addition to predicting the binding profiles, our atomistic models provide an in-depth understanding of the molecular mechanism of action of dual-acting agonists, including Cotadutide, and are relevant for the broad class of candidate peptide coagonist drugs of great clinical interest.^18,32^ Utilizing Cotadutide as a reference peptide not only is important for clarifying the pharmacodynamics of peptide-based therapy but also corroborates our finding that coagonism preserves the native protein fold of both receptors. Our computationally designed new bioactive peptide candidates for GR agonism exploit both the knowledge derived from sequences of known and existing peptide ligands and the structural features of GCGR and GLP-1R. This is coupled in the present work with systematic *in silico* amino acid substitutions to map peptide binding modes and affinities to receptors employing molecular dynamics (MD) simulations to re-engineer these peptide sequences and model bioactive peptide APIs that bind strongly to both GCGR and GLP-1R, a key requirement of peptide coagonists. Our strategy of *in silico* peptide screening coupled with the systematic exploration of the plausible mutational sites could be used to potentially design novel peptide polyagonist sequences in the development of peptide-based APIs for a broad range of GPCRs^41,42^ and other emerging disease targets.^11,43,44^

## 2 Methods

Crystal structures of GCGR and GLP-1R in complex with their endogenous peptide ligands or analogues were obtained from PDB crystal structures 5YQZ(13) (resolution 3 Å) and 6B3J(14) (resolution 3.3 Å), respectively. The 5YQZ structure GCGR is bound to a low-potency partial agonist; thus, it represents the activation helix TM6 in its closed conformation and is considered the inactive state. Hence, we used also the recently solved active state of GCGR with a bound endogenous ligand (crystal structure PDB code 6WPW(45)) and compared it with inactive GCGR. The starting bound poses of PDL-peptides, Cotadutide, and our designed peptide APIs (named MDD_GLP-1R_, MDD_GCGR_, and MDD_GR_ here, see Figure S3) were obtained by superposition of the endogenous ligands on the receptors in the solved structure (further discussed below in subsection 2.1). These ligand–receptor bound complex structures were the starting points for long atomic resolution molecular dynamics (MD) simulations (a total of 2.3 μs of unconstrained dynamics in bulk water; see Table S1) performed using the GROMACS 2018.4^46,47^ code (see subsection 2.2, and further methods/analyses are described under notes S1–S5). All MD simulations converged within the first 0.1 μs of dynamics as monitored from the timelines of fraction of native contacts (*Q(x)*)^48^ (Figure S9), in which a native contact occurs between nonconsecutive residues with a pair of heavy atoms within a cutoff distance of 5 Å. The method provides a good folding coordinate for all-atom simulations and have been previously used to assess coupled helical folding or unfolding and binding events.^49^ We also include timelines of cumulative average secondary structure analyses and RMSD/RMSF (see Figures S4, S5, and S6–S8 discussed under note S1 of the Supporting Information). We note that more coarse-grained methods of native contact analyses such as overlap (OV) and Contacts of Structural Units (CSU) combined maps^50^ and Go̅-like contact maps^51^ may provide detailed information at the residue level without imposing the cutoff distance between residues.

### 2.1 Preparation of the Peptide-Receptor Complex Systems

A recent study by Demartis et al.^40^ screened 35 peptides to select 18 peptides (8 of which showed EC_50_ ≤ 30 pM) as potential coagonists of the glucagon (GCG) and GLP-1 overexpressing receptor cells from phage-displayed peptide libraries, followed by the peptide synthesis. Motivated by these preliminary experimental findings, we developed computational models of dual-acting coagonist peptides to decipher the relationship between molecular-level properties and ligand–receptor structural reorganizations. Here, we have designed the five best-scoring peptide ligands based on their GCG/GLP-1 half-maximal effective concentration (EC_50_) values: sequences #11, #23, #28, #32, and #35 from Table 2 in ref (40).

To mimic the native states under the physiological conditions of peptide-based agonists with GCG and GLP-1 receptors, we set up the simulations with the existing crystal structures of the receptor-peptide complexes. As a starting point, endogenous ligands, the glucagon (GCG) peptide in complex with the GCGR (PDB code 5YQZ,^13^ resolution of 3 Å) and glucagon-like peptide-1 (GLP-1) in complex with the GLP-1R (PDB code 6B3J,^14^ resolution of 3.3 Å), were selected. The extra component crystallized with peptide-receptor systems such as endolysin, G protein, and other small molecular entities was removed from both models. The extracellular domain (ECD), ECLs, and terminals of receptors were modeled using Robetta^53^ (https://robetta.bakerlab.org) which is a protein structure prediction tool and Modeller9.17.^54^ The ECD of receptors was modeled along with the signal sequence, which ideally represents a nascent GPCR case.^55^ The signal peptide is normally cleaved off from the mature protein.^56^ Endogenous ligands (GCG and GLP-1) for their respective receptor system were modeled by substituting the residues on crystallized peptide ligands in the PDB structure. A total of 23 computational models (Table S1) were prepared, out of which 10 models were the GCG/GLP-1 receptor protein in complex with the five selected PDL-peptide coagonists, 6 models of designed MDD-peptides in complex with GRs, 2 models of endogenous ligands (GCG and GLP-1) in complex with their respective receptors, and 2 models of Cotadutide (a reference dual-agonist peptide) in complex with the GCG/GLP-1 receptor and the other two were apo-state GRs. An endogenous peptide agonist (Glucagon) with the GCG receptor (initial structure based on the crystal structure of active GCGR, PDB ID: 6WPW,^45^ resolution of 3.1 Å) was also modeled, and the data obtained from this simulation was used to demonstrate conformational dynamics of active GCGR against inactive GCGR (5YQZ).

Cotadutide, a dual-acting peptide agonist with GCG and GLP-1 activity, is being researched for the potential treatment of disorders like obesity-induced diabetes and nonalcoholic steatohepatitis and is currently under phase-IIb/III clinical trials.^26,32^ Here, we have designed this peptide ligand as a reference peptide in complex with two different receptors, GCGR and GLP-1R, to assess and compare the dual activity against the PDL-peptides and the designed MDD-peptides. We selected Cotadutide as a positive control for this study because it targets the receptor of interest, glucagon, and GLP-1 receptor. We modeled Cotadutide as a helical structure, excluding the fatty acid modification on the Lys^10^ residue.^57^ The PDL/MDD-peptide helical structures of agonists were obtained by incorporating the mutation points on the endogenous ligands (GCG and GLP-1) using the CHARMM-GUI^58^ Web server (https://www.charmm-gui.org). The peptide-receptor complexes were designed using the UCSF Chimera package.^59^ 100 ns MD simulations were performed using GROMACS^27^ MD code with the CHARMM36m all-atom force field and solvated in the water box containing the Charmm-modified TIP3P explicit water model.

### 2.2 Details of MD Simulations

CHARMM36m force field parameters^60^ were used to describe the proteins, and the CHARMM General Force Field (CGenFF)^61,62^ was used to represent the topology and parameters for the ligand. CHARMM-modified TIP3P^63^ was used as a water model, and a minimum distance of 20 Å between any protein atom and any box edge was kept. All MD simulations were carried out using the GROMACS 2018.4^64,65^ packages with an integration time step of 2 fs implemented in the leapfrog integrator^66^ with bond lengths to hydrogen constrained using the LINCS^67^ (protein) and the SETTLE^68^ (water) algorithms. Snapshots were saved every 2 ps. Background ions were added to neutralize protein formal charges and to model the physiological ionic strength (0.15 M NaCl). Long-range electrostatics were treated by the Particle Mesh Ewald (PME) method.^69^ Protein and nonprotein molecules (water and ions) were coupled separately to an external heat bath (298 K) with a coupling time constant of 1 ps using the velocity rescaling method.^70^ All systems were energy minimized and thermalized over 100 ps and equilibrated for 1 ns in the constant volume NVT ensemble followed by another 1 ns of NPT equilibration with the reference pressure at 1 bar and a time constant of 4 ps using the Berendsen barostat.^71^ The production runs were carried out in the constant pressure NPT ensemble using the Parrinello–Rahman barostat.^72^

## 3 Results and Discussion

Motivated by the experimental findings obtained by Demartis et al.^40^ (see section 2.1), we developed computational models of dual-acting coagonist peptides to decipher the relationship between molecular-level properties and ligand–receptor structural reorganizations. We shortlisted five phage-displayed library (PDL)-peptide sequences that ranked highly^40^ in their GCGR/GLP-1R half-maximal effective concentration (EC_50_) ratio (P_11_, P_23_, P_28_, P_32_ and P_35_; see Figure S3) having potential dual coagonist activity for both receptors. We computationally predicted the binding affinities and specificities of these peptides for the two class B1 GPCRs, human GCGR and GLP-1R (Figure S1), to design and model promising peptide-based coagonists that could potentially outperform Cotadutide for cotreatment of T2DM and obesity.

### 3.1 Peptide API Design and Dual-Receptor Targeting Strategy for GRs

#### 3.1.1 Predicted GR Binding Affinity and Molecular Recognition of PDL-Peptides

**Figure 1:**
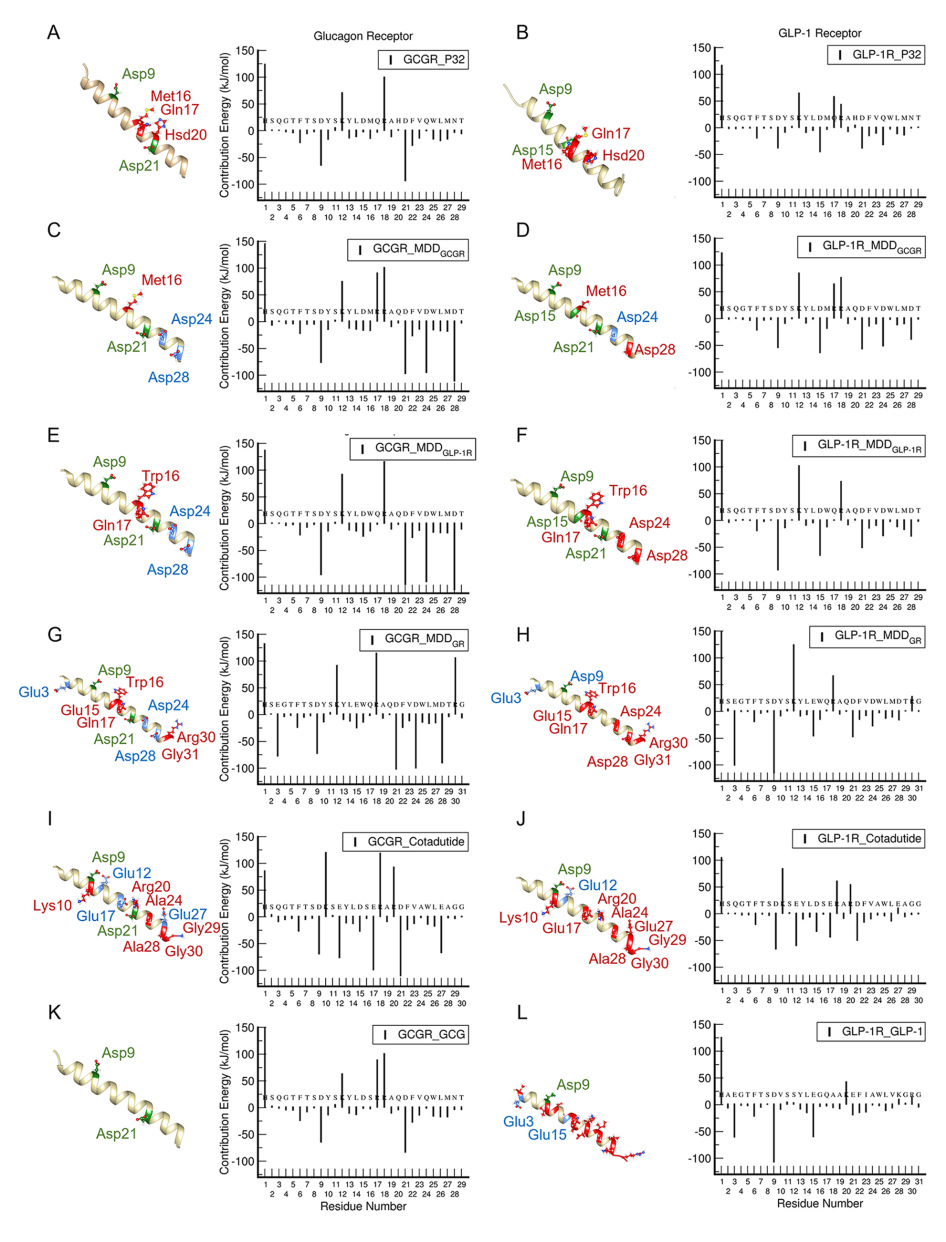
Free energy decomposition showing the contribution of each of the coagonist residues to the total binding energy for PDL-peptide P_32_ (A, B), designed coagonists MDD (C–H), and reference dual agonist peptide Cotadutide (I-J) in complex with GRs and endogenous peptide ligands GCG and GLP-1 in complex with their respective receptor GCGR and GLP-1R (K, L). In the helix structure of peptides, all mutation points are color-coded on the glucagon template in blue or red. The blue color coding identifies mutant residues that significantly contribute to Δ*G*_bind_ with a value of less than −50 kJ/mol. The red color is used for the mutation points with Δ*G*_bind_ values greater than or equal to −50 kJ/mol to ligand binding, while the WT residues that contribute significantly to ligand binding are colored green.

We first make a comprehensive assessment of the molecular recognition features of the native endogenous peptide ligands, the peptides obtained from PDL,^40^ and the reference peptide Cotadutide, by modeling the conformational dynamics and predicting binding affinity of peptide–receptor complexes. We use the data to benchmark the predicted performance of our rationally re-engineered peptide sequences, to gauge their putative superior dual coagonist activity for GCGR and GLP-1R (see Figure S3). The MM/PBSA^73^ method was used to estimate the ligand–receptor binding free energies (Δ*G*_bind_; details in note S2) and rank the affinity of coagonists to GRs. The dielectric constant of protein was set to 2.0.^74,75^ The mean Δ*G*_bind_ values (see Figure S10 A, B) indicate that PDL-peptide P_32_ exhibits the strongest coupled binding affinity to both GCGR and GLP-1R (see Table S2). In addition, P_32_ was found to bind more favorably to GCGR than to GLP-1R compared to other modeled PDL-peptides and also has stronger coupled GR binding affinity than the other PDL-peptides (Table S3). Decomposition of Δ*G*_bind_ per residue (Figure 1 below and Figures S11, S12) reveals the key amino acids that contribute toward Δ*G*_bind_ of coagonists to GRs. Binding is directed by electrostatic attractions between charged/polar sites with a minor contribution from van der Waals (vdW) contacts (Table S4).

#### 3.1.2 Identification of Key Peptide Binding Residues from Thermodynamic Stabilities

Figures S13, S14 show the residual free energy decomposition plots of PDL-peptides to GCGR and GLP-1R, respectively. Binding occurs through a cascade of molecular recognition events between the hydrophilic residues of the coagonist helix and extracellular domain (ECD) and extracellular loops (ECL1/2/3) of the GRs. We observe that acidic residues (Asp and Glu) at positions 9, 21, 24, and 28 on the PDL-peptides make a strong contribution to binding to GCGR (see Table S4), while Asp residues at positions 9 and 15 make strong electrostatic contacts with GLP-1R (Table S4).

#### 3.1.3 Structure-Based Mutagenesis Models to Design Coagonists

Five selected PDL-peptide models (P_11_, P_23_, P_28_, P_32_, and P_35_) and the full-length GCG and GLP-1 peptides were modeled, and the results were used to engineer the candidate MDD coagonist peptide models (discussed below; see Table S5 for mutation points) by *in silico* systematic point mutagenesis to rationally improve binding to both GRs (for details, see note S5). Among the PDL-peptides, P_32_ showed the strongest affinity, and P_11_ showed the weakest binding (see Figure S10 and section 2.2). Our simulation data show that point mutations P_23_^S16W^, P_28_^Q24D^, P_32_^S16M^, P_32_^R17Q^, and P_35_^N28D^ on the PDL-peptides significantly improve the computed peptide affinities to GCGR (Figure S11). On the other hand, GCG mutant P_11_^Q20H^, P_11_^M27L^, P_23_^S16W^, P_28_^Q24D^, P_28_^M27I^, P_32_^S16M^, P_32_^Q20H^, P_35_^M27L^, and P_35_^N28D^ substitutions showed improved predicted affinity for GLP-1R (Figure S12). However, the P_11_^Q20H^, P_11_^M27L^, P_23_^M27Q^, P_28_^M27I^, and P_32_^Q20H^ glucagon point mutations in the PDL-peptides penalize binding to GCGR, and the P_28_^T29S^, P_35_^M27L^, and P_35_^T29S^ point mutations confer no significant advantage for GCGR binding (Tables S6, S7). The predicted stable high-affinity binding of peptides with substitutions at positions 16, 17, 24, and 28 confirms the retention of high potency and structural integrity of native glucagon hormone in agreement with previous alanine scanning studies.^76^ With regards to the PDL-peptide binding, the P_23_^M27Q^, P_28_^T29S^, P_32_^R17Q^, and P_35_^T29S^ substitutions on the glucagon template exhibit negative or no notable predicted agonist binding affinity to GLP-1R, supporting previous alanine substitution experiments at these positions.^77,78^

A significant improvement in the predicted binding energy of P_28_ coagonist to GRs was observed for Q24D substitution and for P_35_ binding to GCGR at the N28D mutation (see Table S7). The point mutations P_23_^S16W^ and P_32_^S16M^ resulted in enhanced predicted affinity over native ligands GCG and GLP-1 at the same position, as predicted from the computed Δ*G*_bind_ values. The P_11_^Q20H^ substitution shows a comparatively lower affinity to bind GCGR compared to the very strong predicted binding to GLP-1R, which is far stronger than the binding of the wild-type GCG peptide to GLP-1R. Mutants of P_32_ at position 20 and P_35_ at residue 27 also show significant improvement over native GLP-1 in binding to GLP-1R. However, the mutation on C-terminus residue 29 (P_28_^T29S^ or P_35_^T29S^) did not significantly alter the predicted binding affinity to GRs. In contrast, the P_32_^R17Q^ substitution led to a massive improvement in the binding of coagonist to GCGR with no major improvement in binding to GLP-1R (Table S7). Hence, by identifying the consequences of residue-level designed mutations on the PDL-peptides through extensive MD simulations, we have systematically characterized the effect of these single residue substitutions on their predicted binding affinities to both GRs. This allowed us to formalize a molecular-level design rule for peptide-based dual coagonists (discussed below).

The significance of mutation points introduced on the WT GCG to effectively target the GCGR and GLP-1R for dual receptor activity can be computationally characterized by the difference maps of contribution energy per residue (Figures S11 and S12). The maps along with our extensive *in silico* mutagenesis analyses above reveal that the substitution of the polar but uncharged residues Gln and Asn with negatively charged residue Asp at residue positions 24 and 28 (see Figure S3) on the WT GCG substantially increases the affinity for coagonist binding to GLP-1R without compromising its binding affinity to GCGR. In addition, the models show that substituting basic residue His with Gln at residue position 20 (Figure 1) does not change the predicted binding affinity of the P_32_ peptide to GCGR (Figure 1K), whereas the H20K substitution improves the overall net peptide binding to GLP-1R despite the large local energy penalty at residue 20 (Figure 1L). Peptide P_23_ hosts aromatic residue Trp at position 16 instead of Ser or Gly in WT GCG or GLP-1, respectively (see Figure S11 and Table S6), and the hydrophobic Trp16 side chain may better anchor the P_23_^S16W^ mutant to the GLP-1R pocket. Thus, compared to the S16 M mutation in P_32_, the S16W mutation in P_23_ shows better predicted binding affinity to GLP-1R (Figure S12 and Table S6). Leu/Iso or Gln (P_11_, P_28_ and P_35_) substituted to Met in P_32_ at position 27 (same as WT GCG) gave a mild binding penalty in comparison to WT GCG on the GCGR (Table S7). On the other hand, the predictions show nonpolar mutations (M27L in P_11_ and P_35_ or M27I in P_28_) improve the binding of PDL-peptides to GLP-1R (Table S7). The residue-wise decomposition energies of PDL-peptides at residue position 27 are −16 and −12 kJ/mol for Iso and Leu (nonpolar, aliphatic mutations), respectively, −1 kJ/mol for Gln (polar, uncharged), and −6 kJ/mol for Val (hydrophobic) in WT GLP-1 (Table S6). Mutation of the P_23_ residue to Gln at position 27 gave a lower predicted binding energy contribution toward both GLP-1R than did the other mutated residues at the 27th position.

We rationalize the consequences of position-specific substitutions of the proposed PDL-peptide constructs^40^ based on the residue-specific energetic contributions and thermodynamics complemented by computational analysis of ligand–receptor dynamics (further discussed below). The simulations predict that the drug performance can be altered with engineered mutations at residue numbers 16, 24, and 28 on the coagonist peptides (Table S7). This rational design approach allows us to identify the effects of mutations on coagonist/receptor binding and reveals that several mutations have a strongly nonadditive effect^40^ on overall binding, reflecting the complex Coulomb sums in the binding pocket and the need for molecular dynamics derived free energy estimates to predict the impact of mutations on binding.^79^

### 3.2 Contribution of Intermolecular Interactions to Coagonist–GR Binding

Hydrogen bonds (H-bonds) play a significant role in facilitating the activation of GPCRs.^80,81^ We mapped the full H-bond networks (see Methods) between peptide APIs and binding pockets of GCGR and GLP-1R. The major donor–acceptor strong H-bonds formed with populations >80% during extended MD are listed in Table S8. We note that residues Ser25^ECD^ (superscript specifies the receptor domains; see Figure S2B), Gln27^ECD^, Asp63^ECD^, Lys64^ECD^, Gln113^ECD^, Gln122^ECD^, Tyr138^TM1^, Gln142^TM1^, Tyr202^TM2^, Ser203^TM2^, Gln204^ECL1^, and Asp385^TM7^ in the GCGR frequently form H-bonds with the coagonist peptides. GLP-1R did not sample any common H-bond-forming residues across the five simulated PDL-peptides, but residues Thr29^ECD^, Ser31^ECD^, Arg121^ECD^, Glu138^TM1^, and Glu387^TM7^ were found to participate in at least four out of five PDL-peptides. Figure S15 shows that the PDL coagonists make more H-bonds with GCGR than with GLP-1R. Despite being one of the least favorable in terms of net binding affinity to GRs, P_11_ samples more H-bonds than other simulated PDL-peptides, which is particularly evident for binding to GCGR (Figure S15A, B). Thus, a simple linear correlation between the number of ligand–receptor H-bonds and predicted binding affinity does not exist due to mixed strong and weak H-bond pairing that directs the binding of P_11_ to GRs consistent with recent findings.^82,83^

Ser2 at the N-terminus of all peptides forms strong H-bonds with Asp385^TM7^ and Glu387^TM7^ of the GCGR and GLP-1R, respectively (see Table S8, Figures S16–S17), while remaining anchored to the GR binding pocket. Ser2–Asp385^TM7^ and Ser2–Asp387^TM7^ H-bonds facilitate an outward movement of TM6 housing residues Phe365^TM6^ and Phe367^TM6^ (Figures S16, S18D). These H-bonded interactions may assist in the activation of GRs (discussed later), where binding of the peptides triggers conformational changes in TM6^84^ at the intracellular domain of GRs following a cascade of signal transduction events. Our findings further corroborate the two-domain binding mechanism as the N-terminal segment of the coagonist peptide binds the ECLs and TMR, which could lead to GR activation and the C-terminal segment of the coagonist binds the ECD, which improves the ligand–receptor binding affinity.^85^

More recently, full-length crystal structures of GRs^13,14^ have revealed the previously unknown agonist binding domains in GRs, which motivated us to further model the dynamics of full-length structures of GRs. Our inspection of these complexes with peptide coagonists identifies intermolecular salt bridges that could impart additional stability to the complex (Figures S19–S20). To investigate the significance of salt bridge formation in coagonist binding to GCGR/GLP-1R, we mapped the specific ligand–receptor residue pairs (see Table S9) involved in salt bridge formation between two oppositely charged residues (average contact distance < 4 Å with their frequency of occurrence being at least 80%; see details of Methods in the Supplementary text). Among all PDL-peptides, we observe weak salt bridges with P_11_ and P_35_ on binding to GCGR. The prominent P_11_ salt bridge Asp9^P11^–Arg378^ECL3^ formed by residue Asp9 in peptide ligands is proposed to be critical for the elicitation of the biological response required for GCGR activation,^86^ consistent with the proposed role of the Asp side chain as a hinge assisting coagonist anchoring to the receptor.^87^ In the P_35_–GCGR complex, the salt bridge between Asp28^P35^ and Arg116^ECD^ dampens the motion of the ECD loop. Asp28^P35^ might be critical when designing dual agonists for GRs, since the N28D substitution in WT GCG decreases its isoelectric point. This significantly improves the aqueous solubility of GCG at physiological pH^88^ which is crucial for peptide drug formulation.

Compared to GCGR, GLP-1R forms few strong salt bridges with the PDL coagonists (Table S9). Peptide P_11_ creates a strong and weak salt bridge via Arg17^P11^. The strong Arg17^P11^–Glu128^ECD^ salt bridge remains stable throughout the dynamics, with the Arg17^P11^–Glu138^TM1^ salt bridge also stabilizing during dynamics. A strong salt bridge is also formed between Arg17^P28^ and Glu138^TM1^ of GLP-1R. P_35_ also samples one salt bridge of moderate strength between Asp9^P35^ and Arg134^ECD^.

### 3.3 Characterization of Cotadutide–GR Binding

A molecular-level understanding of the affinity of Cotadutide (a dual receptor peptide agonist in the drug development pipeline) for GRs and corresponding receptor activation is still lacking. Cotadutide has substitutions on the WT GCG at residue positions 10, 12, 17, 20, 24, 27, and 28 with an added Gly30 residue at the C-terminus end (Figure S3). Here, we model Cotadutide in complex with GRs (Figure S21A) as a control to benchmark the predicted affinities of the PDL and designed peptides and to probe its residue-level structural and thermodynamic effects on the receptors. To formulate a structure-based design rule for dual-agonist peptides, we attempted to answer the following questions: (i) Could we design peptide-based agonists that improve binding to GRs superior to Cotadutide. (ii) How do the positional differences between Cotadutide and the PDL-peptides affect binding to the GRs. (iii) How could the consequential differences in binding affinities of the peptides modulate the activation of GRs at the intracellular domain site.

**Figure 2:**
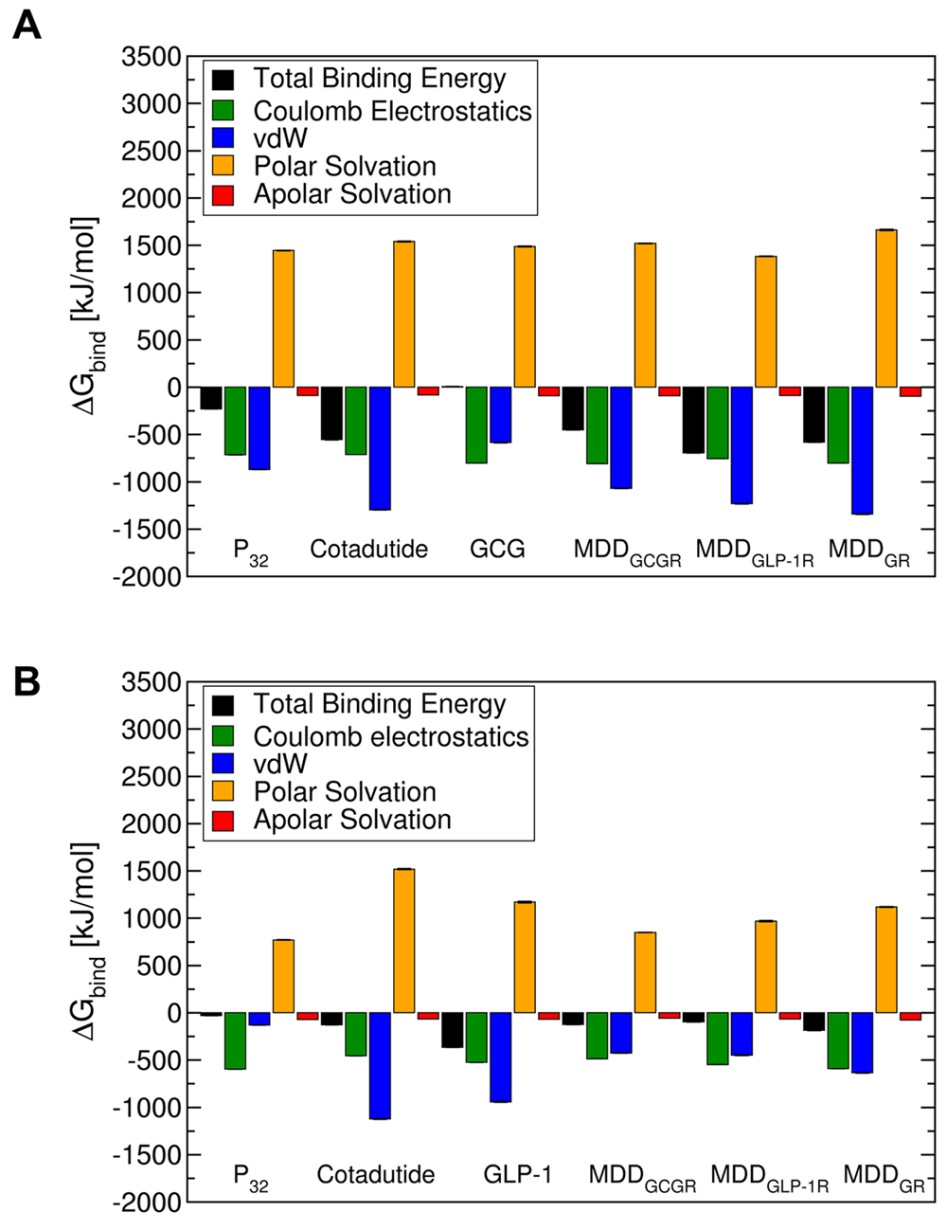
Binding free energy (in kJ/mol) profiles of a PDL coagonist (P_32_), endogenous ligand (GCG), reference dual agonist peptide (Cotadutide), and MDD peptide binding to (A) GCGR and (B) GLP-1R. The average total free energies (Total) are further decomposed into electrostatic, van der Waals (vdW), polar solvation, and nonpolar solvation.

First, we computed the binding affinities of Cotadutide to GRs. The distributions of computed Δ*G*_bind_ reveal more favorable binding to both GCGR and GLP-1R, compared to the GR binding affinities of the PDL-peptides (Figure S10 C, D). In addition, the binding energy data identifies that Cotadutide has better dual agonist affinity toward the GRs than the PDL-peptides (Figure 2). Binding energy decompositions reveal that complexes overcome a large penalty from polar solvation energy to drive favorable steric and electrostatic interactions that stabilize the binding of Cotadutide to both GCGR (Figure 2A) and GLP-1R (Figure 2B).

Next, we predicted the key residues driving the binding of Cotadutide to both GRs. From the residue-wise contributions to Δ*G*_bind_, we observe that negatively charged Asp9, Glu12, Glu17, Asp21, and Glu27 residues promote binding to GCGR, while Asp9 and Glu12 residues stabilize binding to GLP-1R (Figure 1I, J). Residues Glu17 and Asp21 contribute significantly to Cotadutide affinity with GCGR (Figure 1I), while Asp9 and Asp21 stabilize both complexes. These residues also show a large Δ*G*_bind_ contribution across all simulated PDL coagonists except for P_11_ and an endogenous ligand (GCG) (Table S4), forming persistent ligand–receptor H-bonds.

The ligand–receptor residue-wide interaction maps (Figure S21D) reveal strong interactions of Cotadutide with ECD and extracellular loop (ECL) regions of GRs, which may be important to promote molecular recognition of the peptide ligand. Computed H-bond populations show that Cotadutide forms 13 and 9–11 H-bonds with GCGR and GLP-1R, respectively (see Figure S15E, F). Cotadutide with GLP-1R samples a greater number of H-bonds than P_32_ with GLP-1R (Figure S15), whereas for GCGR, the average H-bond counts are similar to the H-bond counts with PDL peptides (Figure S15). Cotadutide forms multiple salt bridges with both receptors (Table S9). The peptide creates three salt bridges with GCGR through Glu27:Arg146^ECD^, Lys10:Asp225^α2^, and Asp15:Arg231^ECL1^. With GLP-1R, P_32_ makes Glu27:Arg151^ECD^, Glu27:Lys160^ECD^, and Asp15:Lys232^ECL1^. The observed strong salt bridging between the CTR of the peptide agonist and ECD of GRs supports the hypothesis that these regions influence agonist binding.^85,89^ The simulation data indicate the involvement of ECL of GRs through salt bridge formation in the agonist binding process, highlighting its importance for designing new dual peptide analogues.

### 3.4 Structural Design and Modeling of Novel Peptide Sequences as Dual Agonists of GRs

#### 3.4.1 MD-Directed Design (MDD) of a GR Coagonist

We computed the molecular recognition features through MD-directed (1.4 μs of free dynamics) binding affinity and specificity for targeting GRs by endogenous ligands, PDL-peptide agonists, and reference peptide Cotadutide. We identify substitutions of uncharged for charged residues at positions 16, 24, and 28 that may modulate the dual-agonist capability of the peptide ligands. It is well documented that certain classes of bioactive peptides have structural features governing their functional selectivity and activity,^10,90,91^ which could be rationally tuned to design next-generation coagonist peptide therapeutics.

**Figure 3:**
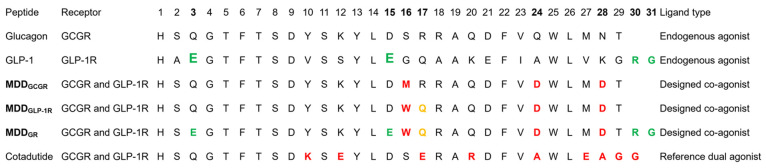
Primary sequence of endogenous ligands (GCG and GLP-1), constructed MD-guided coagonists (MDD_GCGR_, MDD_GLP-1R_, and MDD_GR_) based on the residue-wise decomposition energy data from PDL-peptide/GR simulations, and the reference dual-agonist peptide Cotadutide. In the sequence of MD-guided peptides, all mutation points are color-coded on the glucagon template with red showing phage-displayed peptide (PDL) residues that contributed toward binding with significant Δ*G*_bind_, green showing residues that belong to an endogenous ligand (GLP-1), orange showing the PDL residue with lowest repulsion, and the remaining black residues belonging to template sequence (GCG). The residue font size (in GLP-1 peptide sequence) corresponds to their importance in terms of binding against the same position residue on the GCG peptide with their target receptors, GLP-1R and GCGR, respectively. Color-coding in red on the Cotadutide peptide sequence indicates residue mutation on the peptide sequence of glucagon.

**Figure 4:**
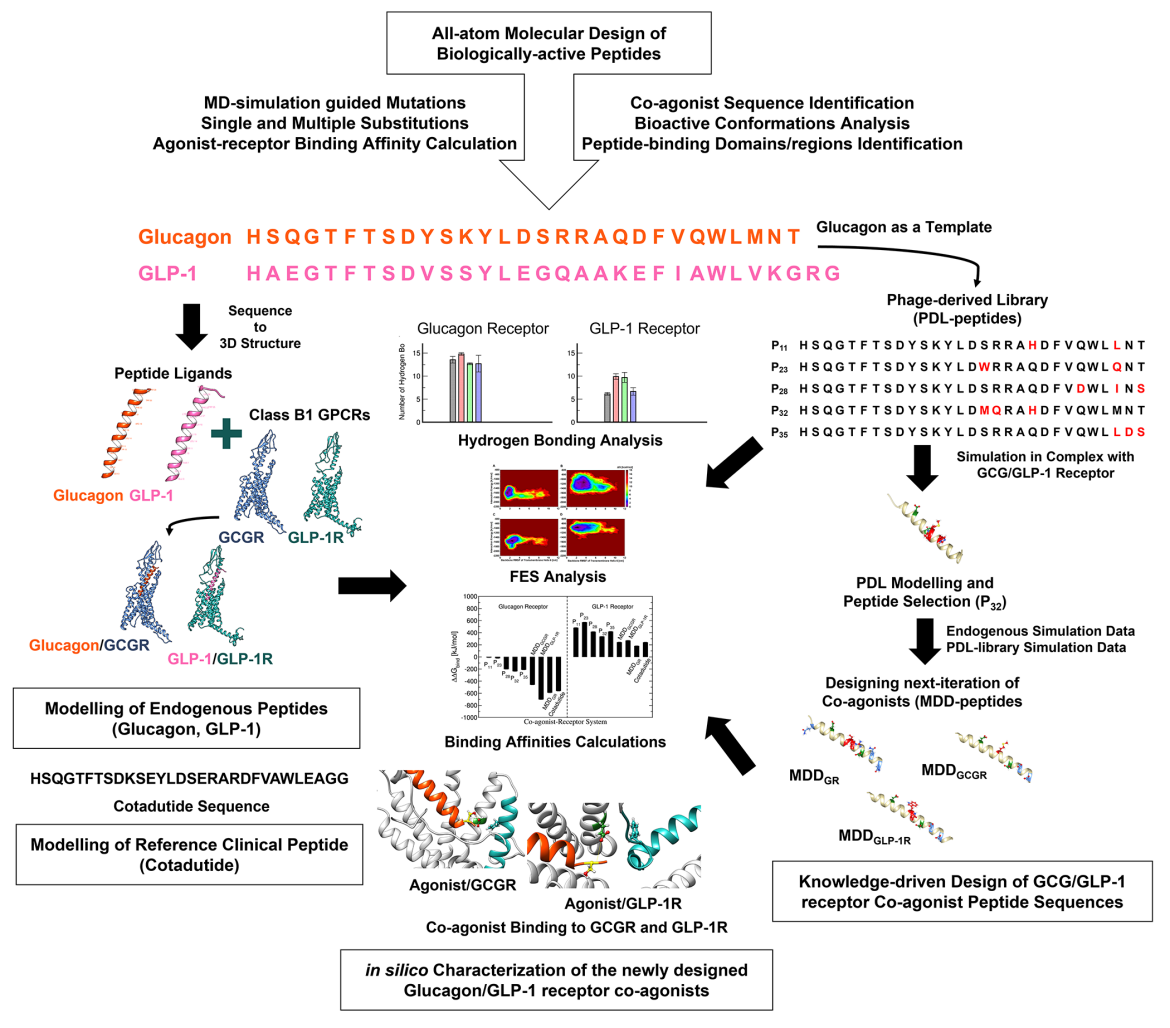
Peptide design strategy for the GCG/GLP-1 receptor coagonist.

The polypeptide sequence selection in the MD-directed Design (MDD)-peptides is based on the residues contributing strongly to binding in the PDL coagonists and the WT GCG polypeptide (see schematic in Figure 3, and also Figure S22 and Figure S23). In addition, we graft the C-terminal sequence of the WT peptide GLP-1 on the WT GCG sequence to design MDD_GR_, so that the coagonist binding affinity toward the ECD could be improved (Figure S24). Though we design the MDD_GCGR_ and MDD_GLP-1R_ (see Table S1) sequences specifically to test against GCGR and GLP-1R respectively as a reference, we also model and study the dynamics of MDD_GCGR_:GLP-1R, and MDD_GLP-1R_:GCGR was used for evaluating cross-affinities of peptides to the receptor. The design of MDD-peptides (Figure 3) was undertaken such that MDD_GCGR_ and MDD_GLP-1R_ each introduced three common mutation sites revealed from our MD analysis above. These are MDD_GCGR_^S16M^, MDD_GCGR_^Q24D^, and MDD_GCGR_^N28D^ for MDD_GCGR_ and MDD_GLP-1R_^S16W^, MDD_GLP-1R_^Q24D^ and MDD_GLP-1R_^N28D^ for MDD_GLP-1R_, with all substitutions modeled on the WT GCG template (Table S5). Additionally, to ensure optimal dual coagonist affinity, the R17Q mutation was introduced to design MDD_GLP-1R_ based on the minimal repulsion shown by Gln17 in P_32_–GCGR simulations coupled with favorable binding of GLP-1R to the peptide with Gln instead of Arg at position 17 (Table S5). With this rational design scheme (Figure 4, and see also Figure S24), we used the MDD_GLP-1R_ as a base sequence to construct MDD_GR_. In the final design step, we mutated back the residues 3 and 15 to Glu (Table S5 and Figure 3) to match the WT GLP-1 peptide since these residues contributed significantly to GLP-1R binding (Figure 1L and Table S6).

#### 3.4.2 Characterization of the Newly Designed MDD Peptide Coagonists

Our predictive analysis suggests an important effect of MDD peptides, in strengthening contacts in the ECD and reducing its flexibility by binding via the C-terminal distal end of the peptide (Figure S25). We hypothesized above that the presence of the C-terminal extended coagonist helix in MDD-GR with Arg30 and Gly31 residues could alter the coagonist binding through ECD stabilization in GRs. However, the free energy decomposition data shows that the contribution of residue Arg30 penalizes binding to the receptor (Table S5 and Figure 1L). We note from the computed structures that GLP-1 residue Gly31 in the extended helix makes strong contacts with GLP-1R, rendering extra support to the C-terminal end.

Comparing the predicted Δ*G*_bind_ of the MDD-designed peptides against the reference dual agonist peptide Cotadutide, our designed *MDD*_*GR*_*peptide is predicted to bind more strongly than Cotadutide to both GCG and GLP-1 receptors*, while MDD_GCGR_ and MDD_GLP-1R_ showed better binding affinity than the PDL peptides but not as strong as Cotadutide (Figure 2). Specifically, the single point mutation Arg to Gln at position 17 in MDD_GLP-1R_ suggests that substitution at this position is crucial for improving binding to GRs (Figures 1E, F) compared to that of WT endogenous peptides. The computed energy contributions to the binding of MDD_GR_ peptide support the significance of R17Q mutation in the design of a potent GR dual coagonist (Figure 1G, H, K, L).

The MD data predicts that MDD_GR_ residues with negatively charged side chains are important for peptide binding to GCGR (E3, D9, D21, D24 and D28) and GLP-1R (E3 and D9) (Table S4 and Figure S26). Figure S15 shows that the number of H-bonds is similar for P_32_ and MDD peptides binding to GCGR. On the other hand, MDD_GR_ has a better propensity to form H-bonds with GLP-1R than the other two MDD-peptides and PDL-peptides (Figure S15). The number of H-bonds formed by MDD_GR_ and P_32_ with the GCG receptor is found to be 15 and 14, respectively. MDD_GR_ forms 9 to 11 H-bonds with GLP-1R (Figure S15F), which is significantly higher than the P_32_–GLP-1R H-bonds (6) and similar to the H-bond count (9–11) of Cotadutide–GLP-1R. Figure S15 shows that our designed peptide MDD_GR_ provides an improved H-bond map compared to the WT peptides with their respective GRs. Our dual coagonist designed MDD-peptides show appreciably high agonist binding GR affinity and could in the future be tested experimentally with or without pharmacokinetic modification through fatty acid cross-linking.^39^

### 3.5 Free-Energy Landscapes and Insights into the Structural Basis of GR Activation by Designed Peptide Coagonists

To investigate the receptor-binding and receptor-activation mechanism of peptide coagonists, we explored the conformational space of the GRs bound to the designed and endogenous ligands (see note S7 of the Supporting Information). We observe that the unstructured N-terminus of the coagonist facilitates its interactions between TM6 and TM7 (Figures S17 and S27E, J), and the C-terminal half of bound-*co*-agonist interacts with the proximal domain of the ECD (see Figures S19, S20). The simulations of the endogenous peptide–GRs complexes reveal that the acidic residues with hydrophilic side chains (Asp9 and Asp21 of GCG and Glu3, Asp9, and Glu15 of GLP-1) facilitate extracellular agonist binding (Table S4). Thus, point mutations at these positions could destabilize agonist–GR complexes and impede the conformational transition necessary for GR activation. Here, we also analyze the agonist-induced conformational changes in the GRs, coupled with domain-specific alteration in the transmembrane domains. We computationally explore the conformational space of high-resolution crystal structures of GRs in unbound (apo) and bound (holo) states which provide atomistic-level insights into GR activation (Figure S28). We studied the effect of the extracellular binding of agonists on the structure of GRs through MD simulations (Figure S18). The structural superposition of inactive and active forms of receptors reveals that a kink is formed in the TM6 helix in both receptors, GCGR and GLP-1R (see Figure S28C, G).

**Figure 5:**
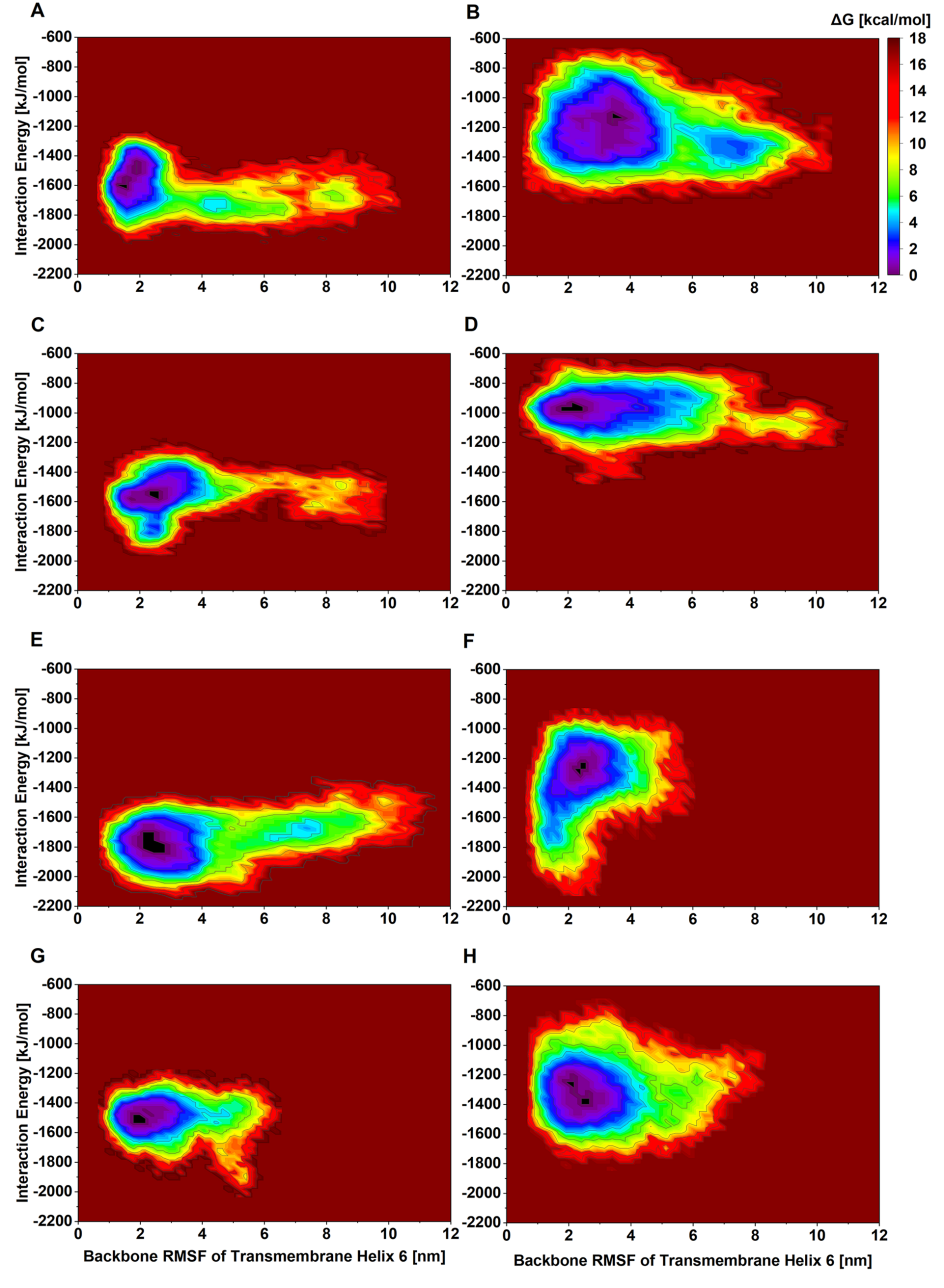
Free energy landscapes of agonist-bound GRs using order parameters, backbone RMSF of transmembrane helix 6, and ligand–receptor interaction energy. (A) Endogenous glucagon-bound GCGR, (b) endogenous GLP-1-bound GLP-1R, (C) PDL peptide (P_32_) coagonist-bound GCGR, (D) PDL peptide (P_32_) coagonist-bound GLP-1R, (E) MDD_GR_ coagonist-bound GCGR, (F) MDD_GR_ coagonist-bound GLP-1R, (G) Cotadutide-bound GCGR, and (H) Cotadutide-bound GLP-1R.

The free energy surfaces (FES) were mapped to study the internal conformation dynamics of GRs upon the extracellular binding of a peptide agonist. To assess the conformational differences in the GRs, we studied the dynamics of both apo- and holo-GR. We calculated the free energy profiles of GRs comparing the inactive and active crystallographic states of both receptors to generate the activation pathway (Figure S29). The FES of GCGR in its apo-state using the order parameters root-mean-square deviation (RMSD) vs. radius of gyration (*R*_g_) shows a densely populated minima over a narrow distribution, highlighting a compact conformation. The location of the minima basin of apo (Figure S29A) and holo (Figure S29B–D) GCGR highlights the structural heterogeneity upon agonist binding, suggesting conformational rearrangements. The FES of the apo state of the GLP-1R (Figure S29E) samples significantly wider conformational space than the peptide-bound GLP-1R holo states (Figure S29F–H). To evaluate peptide agonist binding coupled activation of GRs, in which agonist binding may facilitate the rearrangement of the TM6 helix to stabilize binding cavity formation for the α5 helix of G_αs_,^84^ we plot free energy maps of agonist-receptor interaction energy vs. root-mean-square fluctuation (RMSF) of TM6. The maps reveal that our designed peptide API, MDD_GR_, perturbs helix 6 similar to the endogenous/reference peptide ligands (Figure 5). Overall, the FES of holo-receptor shows that different agonist binding with GRs produces diverse conformational states of GRs, supporting the hypothesis that conformational rearrangement due to ligand binding regulates GR activation. While the present set of 23 0.1 μs MD trajectories provides a robust comparative data set for binding interactions, future work aided by continuous improvements in supercomputing could aim to generate multimicrosecond to millisecond trajectories to provide replicates for enhanced sampling and statistics and possibly capture also collective large-scale motions triggering activation and allostery.^92−94^

## 4 Conclusions

Long-term control of blood glucose levels in patients living with type-2 *diabetes mellitus* (T2DM) is a major challenge for conventional drug therapy. Bioactive peptide-based active pharmaceutical ingredients (APIs) not only have proven antihyperglycemic potential against T2DM by enhancing insulin release via activation of the glucagon-like peptide-1 receptor (GLP-1R) but also suffer from unwanted side effects of induced obesity by suppressing glucagon (GCG) release.^21^ Thus, single receptor agonist (monoagonist) peptide APIs face unmet challenges in the treatment and management of T2DM,^20,26^ requiring peptide APIs targeting and activating both GLP-1R and GCGR for balanced simultaneous agonist action. Although several recent efforts in this field of developing peptides as potential therapeutics of G protein-coupled receptor (GPCR) associated diseases are in clinical trial pipelines,^9^ little is known about the molecular level details^95^ of how the peptides may elicit their coagonist action on GCG and GLP-1 receptors, which hampers rational design of peptide APIs.

To address the above issues, we first evaluated the conformational space and thermodynamic stabilities of known endogenous peptide ligands and putative coagonist peptides from a recent screen by Demartis et al.^40^ which used phage display libraries (PDLs) in complex with the two receptors, GLP-1R and GCGR. We also mapped the conformational stabilities of the experimental dual-agonist peptide Cotadutide in complex with the two receptors. By employing extensive computer molecular dynamics (MD) simulations coupled with free energy calculations of binding affinities predictions and peptide-receptor contact and interaction maps, we show that the PDL peptide coagonist P_32_ shows a relatively higher affinity to both GLP-1R and GCGR compared to other PDL peptides. Thorough screening of the agonist binding pocket of both GCGR and GLP-1R identified the important residues in both receptors, which formed a consensus agonist binding network of acidic Asp and Glu residues favoring agonist binding in the receptor pocket. Using this rationale, combined with the knowledge gained from computed per-residue contributions to the predicted binding, we derived a design rule for three new peptide constructs, named as MD Designer peptides MDD_GCGR_, MDD_GLP-1R_, and MDD_GR_.

The data predicts that our 31-residue coagonist designed peptide, MDD_GR_, has significantly improved binding affinity to both GLP-1R and GCGR, with carefully selected mutation points imparting the dual-receptor agonism. Our computationally designed bioactive peptide APIs may offer a platform for developing and synthesizing dual-acting peptide agonists of GRs and could assist in the future design of bioactive coagonists with improved pharmacodynamics. For example, a recent study indicated that the stabilization of incretins could be enhanced by adding a clustering agent that can force the peptide to oligomerize.^96^ Future studies could employ a side-chain modifier moiety in our designed peptides, which could potentially act as a clustering agent to promote a balanced oligomerization, toward the design of more stable, potent next-generation peptide therapies.

## Data Availability

The focus of our manuscript is on the computational design strategy for therapeutic peptides. The GROMACS 2018.4 software used in this work for running the molecular dynamics simulations is free and open-source software. VMD 1.9.3 and XMGrace programs were used for visualization and plotting, respectively. All peptide models developed in our computational study are de novo designs with their dynamic self-consistency ascertained from the cross-correlation of predicted properties and benchmarked against the available experimental peptide-based drug and native hormones.

## Supporting Information Available

Supplementary notes S1 to S7, Figures S1 to S29, and Tables S1 to S10 (PDF)

## ABBREVIATIONS

API: Active Pharmaceutical Ingredient
Cryo-EM: Cryo-electron Microscopy
CTR: C-terminal Region
ECD: Extracellular Domain
ECL: Extracellular Loop
FDA: U.S. Food and Drug Administration
FES: Free Energy Surfaces
GCG: Glucagon
GCGR: Glucagon Receptor
GLP-1: Glucagon-like Peptide-1
GLP-1R: Glucagon-like Peptide-1 Receptor
GPCR: G Protein-coupled Receptor
GR or GRs: Collectively Glucagon Receptor and Glucagon-like Peptide-1 Receptor
ICD: Intracellular Domain
ICL: Intracellular Loop
MDD-peptides: Molecular Dynamics (MD)-directed Design peptides
MDFE: Molecular Dynamics Free Energy Simulations
MM/PBSA Approach: Molecular Mechanics combined with the Poisson–Boltzmann Surface Area Approach
NTR: N-terminal Region
OXM: Oxyntomodulin
PDL: Phage-displayed Library
PK-modifier: Pharmacokinetic-modifier
*R*_g_: Radius of Gyration
RMSD: Root-mean-square Deviation
RMSF: Root Mean Square Fluctuation
TMR: Transmembrane Region
T2DM: Type-2 *diabetes mellitus*
vdW: van der Waals

## References

[ref1] Katritch V.; Cherezov V.; Stevens R. C. Structure-function of the G protein-coupled receptor superfamily. Annu. Rev. Pharmacol Toxicol 2013, 53, 531–556. 10.1146/annurev-pharmtox-032112-135923. 23140243 PMC3540149

[ref2] Kimura T.; Pydi S. P.; Pham J.; Tanaka N. Metabolic Functions of G Protein-Coupled Receptors in Hepatocytes-Potential Applications for Diabetes and NAFLD. Biomolecules 2020, 10 (10), 1445 10.3390/biom10101445. 33076386 PMC7602561

[ref3] Sriram K.; Insel P. A. G Protein-Coupled Receptors as Targets for Approved Drugs: How Many Targets and How Many Drugs?. Mol. Pharmacol. 2018, 93 (4), 251–258. 10.1124/mol.117.111062. 29298813 PMC5820538

[ref4] Ambery P.; Parker V. E.; Stumvoll M.; Posch M. G.; Heise T.; Plum-Moerschel L.; Tsai L.-F.; Robertson D.; Jain M.; Petrone M.; et al. MEDI0382, a GLP-1 and glucagon receptor dual agonist, in obese or overweight patients with type 2 diabetes: a randomised, controlled, double-blind, ascending dose and phase 2a study. Lancet 2018, 391 (10140), 2607–2618. 10.1016/S0140-6736(18)30726-8. 29945727

[ref5] Sánchez-Garrido M. A.; Brandt S. J.; Clemmensen C.; Müller T. D.; DiMarchi R. D.; Tschöp M. H. GLP-1/glucagon receptor co-agonism for treatment of obesity. Diabetologia 2017, 60 (10), 1851–1861. 10.1007/s00125-017-4354-8. 28733905 PMC6448809

[ref6] Milliken B. T.; Elfers C.; Chepurny O. G.; Chichura K. S.; Sweet I. R.; Borner T.; Hayes M. R.; De Jonghe B. C.; Holz G. G.; Roth C. L.; Doyle R. P. Design and Evaluation of Peptide Dual-Agonists of GLP-1 and NPY2 Receptors for Glucoregulation and Weight Loss with Mitigated Nausea and Emesis. J. Med. Chem. 2021, 64 (2), 1127–1138. 10.1021/acs.jmedchem.0c01783. 33449689 PMC7956155

[ref7] Clemmensen C.; Finan B.; Müller T. D.; DiMarchi R. D.; Tschöp M. H.; Hofmann S. M. Emerging hormonal-based combination pharmacotherapies for the treatment of metabolic diseases. Nat. Rev. Endocrinol 2019, 15 (2), 90–104. 10.1038/s41574-018-0118-x. 30446744

[ref8] Newman D. J.; Cragg G. M. Natural Products as Sources of New Drugs over the Nearly Four Decades from 01/1981 to 09/2019. J. Nat. Prod. 2020, 83 (3), 770–803. 10.1021/acs.jnatprod.9b01285. 32162523

[ref9] Davenport A. P.; Scully C. C. G.; de Graaf C.; Brown A. J. H.; Maguire J. J. Advances in therapeutic peptides targeting G protein-coupled receptors. Nat. Rev. Drug Discov 2020, 19 (6), 389–413. 10.1038/s41573-020-0062-z. 32494050

[ref10] Cong Z.; Liang Y.-L.; Zhou Q.; Darbalaei S.; Zhao F.; Feng W.; Zhao L.; Xu H. E.; Yang D.; Wang M.-W. Structural perspective of class B1 GPCR signaling. Trends Pharmacol. Sci. 2022, 43 (4), 321–334. 10.1016/j.tips.2022.01.002. 35078643

[ref11] Sharma K.; Sharma K. K.; Sharma A.; Jain R. Peptide-based drug discovery: Current status and recent advances. Drug Discovery Today 2023, 28 (2), 103464 10.1016/j.drudis.2022.103464. 36481586

[ref12] Cary B. P.; Zhang X.; Cao J.; Johnson R. M.; Piper S. J.; Gerrard E. J.; Wootten D.; Sexton P. M. New Insights into the Structure and Function of Class B1 GPCRs. Endocr Rev. 2023, 44 (3), 492–517. 10.1210/endrev/bnac033. 36546772 PMC10166269

[ref13] Zhang H.; Qiao A.; Yang L.; Van Eps N.; Frederiksen K. S.; Yang D.; Dai A.; Cai X.; Zhang H.; Yi C.; Cao C.; He L.; Yang H.; Lau J.; Ernst O. P.; Hanson M. A.; Stevens R. C.; Wang M.-W.; Reedtz-Runge S.; Jiang H.; Zhao Q.; Wu B. Structure of the glucagon receptor in complex with a glucagon analogue. Nature 2018, 553 (7686), 106–110. 10.1038/nature25153. 29300013

[ref14] Liang Y.-L.; Khoshouei M.; Glukhova A.; Furness S. G. B.; Zhao P.; Clydesdale L.; Koole C.; Truong T. T.; Thal D. M.; Lei S.; Radjainia M.; Danev R.; Baumeister W.; Wang M.-W.; Miller L. J.; Christopoulos A.; Sexton P. M.; Wootten D. Phase-plate cryo-EM structure of a biased agonist-bound human GLP-1 receptor–Gs complex. Nature 2018, 555 (7694), 121–125. 10.1038/nature25773. 29466332

[ref15] Nauck M. A.; Quast D. R.; Wefers J.; Meier J. J. GLP-1 receptor agonists in the treatment of type 2 diabetes – state-of-the-art. Molecular Metabolism 2021, 46, 101102 10.1016/j.molmet.2020.101102. 33068776 PMC8085572

[ref16] Vilsbøll T. Liraglutide: a human GLP-1 analog for type 2 diabetes. Clinical Practice 2009, 6 (2), 199 10.2217/14750708.6.2.199.

[ref17] Hjalte J.; Hossain S.; Hugerth A.; Sjögren H.; Wahlgren M.; Larsson P.; Lundberg D. Aggregation Behavior of Structurally Similar Therapeutic Peptides Investigated by 1H NMR and All-Atom Molecular Dynamics Simulations. Mol. Pharmaceutics 2022, 19 (3), 904–917. 10.1021/acs.molpharmaceut.1c00883. PMC8905580 35104408

[ref18] Nahra R.; Wang T.; Gadde K. M.; Oscarsson J.; Stumvoll M.; Jermutus L.; Hirshberg B.; Ambery P. Effects of Cotadutide on Metabolic and Hepatic Parameters in Adults With Overweight or Obesity and Type 2 Diabetes: A 54-Week Randomized Phase 2b Study. Diabetes Care 2021, 44 (6), 1433–1442. 10.2337/dc20-2151. 34016612 PMC8247525

[ref19] Firouzjaei A.; Li G. C.; Wang N.; Liu W. X.; Zhu B. M. Comparative evaluation of the therapeutic effect of metformin monotherapy with metformin and acupuncture combined therapy on weight loss and insulin sensitivity in diabetic patients. Nutrition & Diabetes 2016, 6 (5), e209–e209. 10.1038/nutd.2016.16. 27136447 PMC4895377

[ref20] Alam F.; Islam M. A.; Mohamed M.; Ahmad I.; Kamal M. A.; Donnelly R.; Idris I.; Gan S. H. Efficacy and Safety of Pioglitazone Monotherapy in Type 2 Diabetes Mellitus: A Systematic Review and Meta-Analysis of Randomised Controlled Trials. Sci. Rep. 2019, 9 (1), 5389 10.1038/s41598-019-41854-2. 30926892 PMC6441028

[ref21] Drucker D. J. Mechanisms of Action and Therapeutic Application of Glucagon-like Peptide-1. Cell Metabolism 2018, 27 (4), 740–756. 10.1016/j.cmet.2018.03.001. 29617641

[ref22] Tahrani A. A.; Piya M. K.; Kennedy A.; Barnett A. H. Glycaemic control in type 2 diabetes: Targets and new therapies. Pharmacology & Therapeutics 2010, 125 (2), 328–361. 10.1016/j.pharmthera.2009.11.001. 19931305

[ref23] Jepsen M. M.; Christensen M. B. Emerging glucagon-like peptide 1 receptor agonists for the treatment of obesity. Expert Opinion on Emerging Drugs 2021, 26 (3), 231–243. 10.1080/14728214.2021.1947240. 34176426

[ref24] Nestor J. J.; Parkes D.; Feigh M.; Suschak J. J.; Harris M. S. Effects of ALT-801, a GLP-1 and glucagon receptor dual agonist, in a translational mouse model of non-alcoholic steatohepatitis. Sci. Rep. 2022, 12 (1), 6666 10.1038/s41598-022-10577-2. 35461369 PMC9035150

[ref25] Jastreboff A. M.; Aronne L. J.; Ahmad N. N.; Wharton S.; Connery L.; Alves B.; Kiyosue A.; Zhang S.; Liu B.; Bunck M. C.; Stefanski A. Tirzepatide Once Weekly for the Treatment of Obesity. New England Journal of Medicine 2022, 387 (3), 205–216. 10.1056/NEJMoa2206038. 35658024

[ref26] Tan T. M. M. Co-agonist therapeutics come of age for obesity. Nature Reviews Endocrinology 2023, 19 (2), 66–67. 10.1038/s41574-022-00788-y. 36526874

[ref27] Bailey C. J.; Flatt P. R.; Conlon J. M. An update on peptide-based therapies for type 2 diabetes and obesity. Peptides 2023, 161, 170939 10.1016/j.peptides.2023.170939. 36608818

[ref28] Spezani R.; Mandarim-de-Lacerda C. A. The current significance and prospects for the use of dual receptor agonism GLP-1/Glucagon. Life Sciences 2022, 288, 120188 10.1016/j.lfs.2021.120188. 34861287

[ref29] Hauser A. S.; Kooistra A. J.; Munk C.; Heydenreich F. M.; Veprintsev D. B.; Bouvier M.; Babu M. M.; Gloriam D. E. GPCR activation mechanisms across classes and macro/microscales. Nature Structural & Molecular Biology 2021, 28 (11), 879–888. 10.1038/s41594-021-00674-7. PMC8580822 34759375

[ref30] Longwell C. K.; Hanna S.; Hartrampf N.; Sperberg R. A. P.; Huang P. S.; Pentelute B. L.; Cochran J. R. Identification of N-Terminally Diversified GLP-1R Agonists Using Saturation Mutagenesis and Chemical Design. ACS Chem. Biol. 2021, 16 (1), 58–66. 10.1021/acschembio.0c00722. 33307682 PMC8068314

[ref31] Pocai A.; Carrington P. E.; Adams J. R.; Wright M.; Eiermann G.; Zhu L.; Du X.; Petrov A.; Lassman M. E.; Jiang G.; Liu F.; Miller C.; Tota L. M.; Zhou G.; Zhang X.; Sountis M. M.; Santoprete A.; Capito E.; Chicchi G. G.; Thornberry N.; Bianchi E.; Pessi A.; Marsh D. J.; SinhaRoy R. Glucagon-like peptide 1/glucagon receptor dual agonism reverses obesity in mice. Diabetes 2009, 58 (10), 2258–66. 10.2337/db09-0278. 19602537 PMC2750209

[ref32] Boland M. L.; Laker R. C.; Mather K.; Nawrocki A.; Oldham S.; Boland B. B.; Lewis H.; Conway J.; Naylor J.; Guionaud S.; Feigh M.; Veidal S. S.; Lantier L.; McGuinness O. P.; Grimsby J.; Rondinone C. M.; Jermutus L.; Larsen M. R.; Trevaskis J. L.; Rhodes C. J. Resolution of NASH and hepatic fibrosis by the GLP-1R and GCGR dual-agonist cotadutide via modulating mitochondrial function and lipogenesis. Nature Metabolism 2020, 2 (5), 413–431. 10.1038/s42255-020-0209-6. PMC7258337 32478287

[ref33] Robertson D.; Hansen L.; Ambery P.; Esterline R. L.; Jermutus L.; Chang Y.-T.; Petrone M.; Johansson E.; Johansson L.; SjÖBerg F. B.; Parker V. 354-OR: Cotadutide (medi0382), a Dual Receptor Agonist with Glucagon-Like Peptide-1 and Glucagon Activity, Modulates Hepatic Glycogen and Fat Content. Diabetes 2020, 69 (Supplement_1), 354-OR 10.2337/db20-354-OR.

[ref34] Parker V. E. R.; Robertson D.; Wang T.; Hornigold D. C.; Petrone M.; Cooper A. T.; Posch M. G.; Heise T.; Plum-Moerschel L.; Schlichthaar H.; Klaus B.; Ambery P. D.; Meier J. J.; Hirshberg B. Efficacy, Safety, and Mechanistic Insights of Cotadutide, a Dual Receptor Glucagon-Like Peptide-1 and Glucagon Agonist. Journal of Clinical Endocrinology & Metabolism 2020, 105 (3), 803–820. 10.1210/clinem/dgz047. 31608926

[ref35] Ali M.; Hafez A.; Abdelgalil M. S.; Hasan M. T.; El-Ghannam M. M.; Ghogar O.; Elrashedy A. A.; Abd-ElGawad M. Impact of Cotadutide Drug on Obese Individuals with Type 2 Diabetes Mellitus: A Systematic Review and Meta-analysis. BMC Endocrine Disorders 2022, 22, 113 10.1186/s12902-022-01031-5. 35488292 PMC9055739

[ref36] Scheen A. J. Dual GIP/GLP-1 receptor agonists: New advances for treating type-2 diabetes. Annales d’Endocrinologie 2023, 84, 316 10.1016/j.ando.2022.12.423. 36639119

[ref37] Nauck M. A.; D'Alessio D. A. Tirzepatide, a dual GIP/GLP-1 receptor co-agonist for the treatment of type 2 diabetes with unmatched effectiveness regrading glycaemic control and body weight reduction. Cardiovascular Diabetology 2022, 21 (1), 169 10.1186/s12933-022-01604-7. 36050763 PMC9438179

[ref38] Finan B.; Douros J. D. GLP-1/GIP/glucagon receptor triagonism gets its try in humans. Cell Metabolism 2022, 34 (1), 3–4. 10.1016/j.cmet.2021.12.010. 34986336

[ref39] Zhihong Y.; Chen W.; Qianqian Z.; Lidan S.; Qiang Z.; Jing H.; Wenxi W.; Bhawal R. Emerging roles of oxyntomodulin-based glucagon-like peptide-1/glucagon co-agonist analogs in diabetes and obesity. Peptides 2023, 162, 170955 10.1016/j.peptides.2023.170955. 36669563

[ref40] Demartis A.; Lahm A.; Tomei L.; Beghetto E.; Di Biasio V.; Orvieto F.; Frattolillo F.; Carrington P. E.; Mumick S.; Hawes B.; Bianchi E.; Palani A.; Pessi A. Polypharmacy through Phage Display: Selection of Glucagon and GLP-1 Receptor Co-agonists from a Phage-Displayed Peptide Library. Sci. Rep 2018, 8 (1), 585 10.1038/s41598-017-18494-5. 29330364 PMC5766609

[ref41] Pocai A. Unraveling oxyntomodulin, GLP1’s enigmatic brother. Journal of endocrinology 2012, 215 (3), 335–346. 10.1530/JOE-12-0368. 23019069 PMC3493657

[ref42] Price S. L.; Minnion J. S.; Bloom S. R. Investigating the Glucagon Receptor and Glucagon-Like Peptide 1 Receptor Activity of Oxyntomodulin-Like Analogues in Male Wistar Rats. Current Therapeutic Research 2015, 77, 111–115. 10.1016/j.curtheres.2015.10.003. 26843896 PMC4701715

[ref43] Wang L.; Wang N.; Zhang W.; Cheng X.; Yan Z.; Shao G.; Wang X.; Wang R.; Fu C. Therapeutic peptides: current applications and future directions. Signal Transduction and Targeted Therapy 2022, 7 (1), 48 10.1038/s41392-022-00904-4. 35165272 PMC8844085

[ref44] Li C. M.; Haratipour P.; Lingeman R. G.; Perry J. J. P.; Gu L.; Hickey R. J.; Malkas L. H. Novel Peptide Therapeutic Approaches for Cancer Treatment. Cells 2021, 10 (11), 2908 10.3390/cells10112908. 34831131 PMC8616177

[ref45] Hilger D.; Kumar K. K.; Hu H.; Pedersen M. F.; O’Brien E. S.; Giehm L.; Jennings C.; Eskici G.; Inoue A.; Lerch M.; Mathiesen J. M.; Skiniotis G.; Kobilka B. K. Structural insights into differences in G protein activation by family A and family B GPCRs. Science 2020, 369 (6503), eaba3373 10.1126/science.aba3373. 32732395 PMC7954662

[ref46] Abraham M. J.; Murtola T.; Schulz R.; Páll S.; Smith J. C.; Hess B.; Lindahl E. GROMACS: High performance molecular simulations through multi-level parallelism from laptops to supercomputers. SoftwareX 2015, 1, 19–25. 10.1016/j.softx.2015.06.001.

[ref47] Van Der Spoel D.; Lindahl E.; Hess B.; Groenhof G.; Mark A. E.; Berendsen H. J. GROMACS: fast, flexible, and free. J. Comput. Chem. 2005, 26 (16), 1701–18. 10.1002/jcc.20291. 16211538

[ref48] Best R. B.; Hummer G.; Eaton W. A. Native contacts determine protein folding mechanisms in atomistic simulations. Proc. Natl. Acad. Sci. U. S. A. 2013, 110 (44), 17874–9. 10.1073/pnas.1311599110. 24128758 PMC3816414

[ref49] Bhattacharya S.; Xu L.; Thompson D. Molecular Simulations Reveal Terminal Group Mediated Stabilization of Helical Conformers in Both Amyloid-beta42 and alpha-Synuclein. ACS Chem. Neurosci. 2019, 10 (6), 2830–2842. 10.1021/acschemneuro.9b00053. 30917651

[ref50] Wolek K.; Gomez-Sicilia A.; Cieplak M. Determination of contact maps in proteins: A combination of structural and chemical approaches. J. Chem. Phys. 2015, 143 (24), 243105 10.1063/1.4929599. 26723590

[ref51] Poma A. B.; Cieplak M.; Theodorakis P. E. Combining the MARTINI and Structure-Based Coarse-Grained Approaches for the Molecular Dynamics Studies of Conformational Transitions in Proteins. J. Chem. Theory Comput 2017, 13 (3), 1366–1374. 10.1021/acs.jctc.6b00986. 28195464

[ref53] Kim D. E.; Chivian D.; Baker D. Protein structure prediction and analysis using the Robetta server. Nucleic Acids Res. 2004, 32 (Web Server), W526–31. 10.1093/nar/gkh468. 15215442 PMC441606

[ref54] Webb B.; Sali A. Protein structure modeling with MODELLER. Methods Mol. Biol. 2014, 1137, 1–15. 10.1007/978-1-4939-0366-5_1. 24573470

[ref55] Fasciani I.; Carli M.; Petragnano F.; Colaianni F.; Aloisi G.; Maggio R.; Scarselli M.; Rossi M. GPCRs in Intracellular Compartments: New Targets for Drug Discovery. Biomolecules 2022, 12 (10), 1343 10.3390/biom12101343. 36291552 PMC9599219

[ref56] Ge Y.; Yang D.; Dai A.; Zhou C.; Zhu Y.; Wang M. W. The putative signal peptide of glucagon-like peptide-1 receptor is not required for receptor synthesis but promotes receptor expression. Biosci Rep 2014, 34 (6), e00152 10.1042/BSR20140120. 25330813 PMC4240022

[ref57] Henderson S. J.; Konkar A.; Hornigold D. C.; Trevaskis J. L.; Jackson R.; Fritsch Fredin M.; Jansson-Löfmark R.; Naylor J.; Rossi A.; Bednarek M. A.; Bhagroo N.; Salari H.; Will S.; Oldham S.; Hansen G.; Feigh M.; Klein T.; Grimsby J.; Maguire S.; Jermutus L.; Rondinone C. M.; Coghlan M. P. Robust anti-obesity and metabolic effects of a dual GLP-1/glucagon receptor peptide agonist in rodents and non-human primates. Diabetes, Obesity and Metabolism 2016, 18 (12), 1176–1190. 10.1111/dom.12735. PMC5129521 27377054

[ref58] Wu E. L.; Cheng X.; Jo S.; Rui H.; Song K. C.; Dávila-Contreras E. M.; Qi Y.; Lee J.; Monje-Galvan V.; Venable R. M.; Klauda J. B.; Im W. CHARMM-GUI Membrane Builder toward realistic biological membrane simulations. J. Comput. Chem. 2014, 35 (27), 1997–2004. 10.1002/jcc.23702. 25130509 PMC4165794

[ref59] Pettersen E. F.; Goddard T. D.; Huang C. C.; Couch G. S.; Greenblatt D. M.; Meng E. C.; Ferrin T. E. UCSF Chimera--a visualization system for exploratory research and analysis. J. Comput. Chem. 2004, 25 (13), 1605–12. 10.1002/jcc.20084. 15264254

[ref60] Huang J.; Rauscher S.; Nawrocki G.; Ran T.; Feig M.; de Groot B. L.; Grubmüller H.; MacKerell A. D. Jr. CHARMM36m: an improved force field for folded and intrinsically disordered proteins. Nat. Methods 2017, 14 (1), 71–73. 10.1038/nmeth.4067. 27819658 PMC5199616

[ref61] Vanommeslaeghe K.; Hatcher E.; Acharya C.; Kundu S.; Zhong S.; Shim J.; Darian E.; Guvench O.; Lopes P.; Vorobyov I.; Mackerell A. D. Jr. CHARMM general force field: A force field for drug-like molecules compatible with the CHARMM all-atom additive biological force fields. J. Comput. Chem. 2010, 31 (4), 671–90. 10.1002/jcc.21367. 19575467 PMC2888302

[ref62] Yu W.; He X.; Vanommeslaeghe K.; MacKerell A. D. Jr. Extension of the CHARMM General Force Field to sulfonyl-containing compounds and its utility in biomolecular simulations. J. Comput. Chem. 2012, 33 (31), 2451–68. 10.1002/jcc.23067. 22821581 PMC3477297

[ref63] MacKerell A. D. Jr.; Bashford D.; Bellott M.; Dunbrack R. L. Jr.; Evanseck J. D.; Field M. J.; Fischer S.; Gao J.; Guo H.; Ha S.; Joseph-McCarthy D.; Kuchnir L.; Kuczera K.; Lau F. T. K.; Mattos C.; Michnick S.; Ngo T.; Nguyen D. T.; Prodhom B.; Reiher W. E.; Roux B.; Schlenkrich M.; Smith J. C.; Stote R.; Straub J.; Watanabe M.; Wiórkiewicz-Kuczera J.; Yin D.; Karplus M. All-Atom Empirical Potential for Molecular Modeling and Dynamics Studies of Proteins. J. Phys. Chem. B 1998, 102 (18), 3586–3616. 10.1021/jp973084f. 24889800

[ref64] Berendsen H. J. C.; van der Spoel D.; van Drunen R. GROMACS: A message-passing parallel molecular dynamics implementation. Comput. Phys. Commun. 1995, 91 (1), 43–56. 10.1016/0010-4655(95)00042-E.

[ref65] Van Der Spoel D.; Lindahl E.; Hess B.; Groenhof G.; Mark A. E.; Berendsen H. J. C. GROMACS: Fast, flexible, and free. J. Comput. Chem. 2005, 26 (16), 1701–1718. 10.1002/jcc.20291. 16211538

[ref66] Hockney R. W. The Potenitial Calculation and Some Applications. Methods Comput. Phys. 1970, 20, 135.

[ref67] Hess B.; Bekker H.; Berendsen H. J. C.; Fraaije J. G. E. M. LINCS: A linear constraint solver for molecular simulations. J. Comput. Chem. 1997, 18 (12), 1463–1472. 10.1002/(SICI)1096-987X(199709)18:12<1463::AID-JCC4>3.0.CO;2-H.

[ref68] Miyamoto S.; Kollman P. A. Settle: An analytical version of the SHAKE and RATTLE algorithm for rigid water models. J. Comput. Chem. 1992, 13 (8), 952–962. 10.1002/jcc.540130805.

[ref69] Darden T.; York D.; Pedersen L. Particle mesh Ewald: An N·log(N) method for Ewald sums in large systems. J. Chem. Phys. 1993, 98 (12), 10089–10092. 10.1063/1.464397.

[ref70] Bussi G.; Donadio D.; Parrinello M. Canonical sampling through velocity rescaling. J. Chem. Phys. 2007, 126 (1), 014101 10.1063/1.2408420. 17212484

[ref71] Berendsen H. J. C.; Postma J. P. M.; Gunsteren W. F. v.; DiNola A.; Haak J. R. Molecular dynamics with coupling to an external bath. J. Chem. Phys. 1984, 81 (8), 3684–3690. 10.1063/1.448118.

[ref72] Parrinello M.; Rahman A. Polymorphic transitions in single crystals: A new molecular dynamics method. J. Appl. Phys. 1981, 52 (12), 7182–7190. 10.1063/1.328693.

[ref73] Kumari R.; Kumar R.; Lynn A. g_mmpbsa—A GROMACS Tool for High-Throughput MM-PBSA Calculations. J. Chem. Inf. Model. 2014, 54 (7), 1951–1962. 10.1021/ci500020m. 24850022

[ref74] Fogolari F.; Brigo A.; Molinari H. Protocol for MM/PBSA Molecular Dynamics Simulations of Proteins. Biophys. J. 2003, 85 (1), 159–166. 10.1016/S0006-3495(03)74462-2. 12829472 PMC1303073

[ref75] Schutz C. N.; Warshel A. What are the dielectric “constants” of proteins and how to validate electrostatic models?. Proteins: Struct., Funct., Bioinf. 2001, 44 (4), 400–417. 10.1002/prot.1106. 11484218

[ref76] Chabenne J.; Chabenne M. D.; Zhao Y.; Levy J.; Smiley D.; Gelfanov V.; DiMarchi R. A glucagon analog chemically stabilized for immediate treatment of life-threatening hypoglycemia. Molecular Metabolism 2014, 3 (3), 293–300. 10.1016/j.molmet.2014.01.006. 24749059 PMC3986664

[ref77] Gallwitz B.; Witt M.; Paetzold G.; Morys-Wortmann C.; Zimmermann B.; Eckart K.; Fölsch U. R.; Schmidt W. E. Structure/activity characterization of glucagon-like peptide-1. Eur. J. Biochem. 1994, 225 (3), 1151–6. 10.1111/j.1432-1033.1994.1151b.x. 7957206

[ref78] Adelhorst K.; Hedegaard B. B.; Knudsen L. B.; Kirk O. Structure-activity studies of glucagon-like peptide-1. J. Biol. Chem. 1994, 269 (9), 6275–8. 10.1016/S0021-9258(17)37366-0. 8119974

[ref79] Thompson D.; Lazennec C.; Plateau P.; Simonson T. Ammonium Scanning in an Enzyme Active Site: THE CHIRAL SPECIFICITY OF ASPARTYL-tRNA SYNTHETASE. J. Biol. Chem. 2007, 282 (42), 30856–30868. 10.1074/jbc.M704788200. 17690095

[ref80] Cabana J.; Holleran B.; Beaulieu M.-È.; Leduc R.; Escher E.; Guillemette G.; Lavigne P. Critical Hydrogen Bond Formation for Activation of the Angiotensin II Type 1 Receptor *. J. Biol. Chem. 2013, 288 (4), 2593–2604. 10.1074/jbc.M112.395939. 23223579 PMC3554926

[ref81] Zhang X.; Belousoff M. J.; Zhao P.; Kooistra A. J.; Truong T. T.; Ang S. Y.; Underwood C. R.; Egebjerg T.; Šenel P.; Stewart G. D.; Liang Y.-L.; Glukhova A.; Venugopal H.; Christopoulos A.; Furness S. G. B.; Miller L. J.; Reedtz-Runge S.; Langmead C. J.; Gloriam D. E.; Danev R.; Sexton P. M.; Wootten D. Differential GLP-1R Binding and Activation by Peptide and Non-peptide Agonists. Mol. Cell 2020, 80 (3), 485–500.e7. 10.1016/j.molcel.2020.09.020. 33027691

[ref82] Chen D.; Oezguen N.; Urvil P.; Ferguson C.; Dann S. M.; Savidge T. C. Regulation of protein-ligand binding affinity by hydrogen bond pairing. Science advances 2016, 2 (3), e1501240 10.1126/sciadv.1501240. 27051863 PMC4820369

[ref83] Chodera J. D.; Mobley D. L. Entropy-enthalpy compensation: role and ramifications in biomolecular ligand recognition and design. Annu. Rev. Biophys 2013, 42, 121–142. 10.1146/annurev-biophys-083012-130318. 23654303 PMC4124006

[ref84] Mattedi G.; Acosta-Gutiérrez S.; Clark T.; Gervasio F. L. A combined activation mechanism for the glucagon receptor. Proc. Natl. Acad. Sci. U. S. A. 2020, 117 (27), 15414 10.1073/pnas.1921851117. 32571939 PMC7355025

[ref85] Underwood C. R.; Garibay P.; Knudsen L. B.; Hastrup S.; Peters G. H.; Rudolph R.; Reedtz-Runge S. Crystal structure of glucagon-like peptide-1 in complex with the extracellular domain of the glucagon-like peptide-1 receptor. J. Biol. Chem. 2010, 285 (1), 723–730. 10.1074/jbc.M109.033829. 19861722 PMC2804221

[ref86] Unson C. G.; Macdonald D.; Ray K.; Durrah T. L.; Merrifield R. B. Position 9 replacement analogs of glucagon uncouple biological activity and receptor binding. J. Biol. Chem. 1991, 266 (5), 2763–6. 10.1016/S0021-9258(18)49911-5. 1847133

[ref87] Unson C. G.; Wu C. R.; Merrifield R. B. Roles of aspartic acid 15 and 21 in glucagon action: receptor anchor and surrogates for aspartic acid 9. Biochemistry 1994, 33 (22), 6884–7. 10.1021/bi00188a018. 8204623

[ref88] Müller T. D.; Finan B.; Clemmensen C.; DiMarchi R. D.; Tschöp M. H. The New Biology and Pharmacology of Glucagon. Physiol. Rev. 2017, 97 (2), 721–766. 10.1152/physrev.00025.2016. 28275047

[ref89] Wu F.; Yang L.; Hang K.; Laursen M.; Wu L.; Han G. W.; Ren Q.; Roed N. K.; Lin G.; Hanson M. A.; Jiang H.; Wang M.-W.; Reedtz-Runge S.; Song G.; Stevens R. C. Full-length human GLP-1 receptor structure without orthosteric ligands. Nat. Commun. 2020, 11 (1), 1272 10.1038/s41467-020-14934-5. 32152292 PMC7062719

[ref90] Yang F.-j.; Chen X.; Huang M.-c.; Yang Q.; Cai X.-x.; Chen X.; Du M.; Huang J.-l.; Wang S.-y. Molecular characteristics and structure–activity relationships of food-derived bioactive peptides. Journal of Integrative Agriculture 2021, 20 (9), 2313–2332. 10.1016/S2095-3119(20)63463-3.

[ref91] Zhang X.; Cai Y.; Yao Z.; Chi H.; Li Y.; Shi J.; Zhou Z.; Sun L. Discovery of novel OXM-based glucagon-like peptide 1 (GLP-1)/glucagon receptor dual agonists. Peptides 2023, 161, 170948 10.1016/j.peptides.2023.170948. 36646385

[ref92] Lu S.; He X.; Yang Z.; Chai Z.; Zhou S.; Wang J.; Rehman A. U.; Ni D.; Pu J.; Sun J.; Zhang J. Activation pathway of a G protein-coupled receptor uncovers conformational intermediates as targets for allosteric drug design. Nat. Commun. 2021, 12 (1), 4721 10.1038/s41467-021-25020-9. 34354057 PMC8342441

[ref93] Latorraca N. R.; Venkatakrishnan A. J.; Dror R. O. GPCR Dynamics: Structures in Motion. Chem. Rev. 2017, 117 (1), 139–155. 10.1021/acs.chemrev.6b00177. 27622975

[ref94] Abrol R.; Serrano E.; Santiago L. J. Development of enhanced conformational sampling methods to probe the activation landscape of GPCRs. Adv. Protein Chem. Struct Biol. 2022, 128, 325–359. 10.1016/bs.apcsb.2021.11.001. 35034722 PMC11476118

[ref95] Yaqub T.; Tikhonova I. G.; Lättig J.; Magnan R.; Laval M.; Escrieut C.; Boulègue C.; Hewage C.; Fourmy D. Identification of Determinants of Glucose-Dependent Insulinotropic Polypeptide Receptor That Interact with N-Terminal Biologically Active Region of the Natural Ligand. Mol. Pharmacol. 2010, 77 (4), 547 10.1124/mol.109.060111. 20061446

[ref96] Li Y.; Shao M.; Zheng X.; Kong W.; Zhang J.; Gong M. Self-assembling peptides improve the stability of glucagon-like peptide-1 by forming a stable and sustained complex. Mol. Pharmaceutics 2013, 10 (9), 3356–65. 10.1021/mp4001734. 23859692

