# Supplementary material for: Computational Peptide Design Cotargeting Glucagon and Glucagon-like Peptide-1 Receptors
Source: J Chem Inf Model. 2023 Jul 31;63(15):4934–47. doi: 10.1021/acs.jcim.3c00752 (PMC10428222; doi:10.1021/acs.jcim.3c00752)
Supplement: Supplementary file 1 — ci3c00752_si_001.pdf [file ci3c00752_si_001.pdf]

Supporting Information

Computational peptide design cotargeting glucagon and glucagon-like peptide-1 receptors

Shubham Vishnoi[1], Shayon Bhattacharya[1*], Erica M. Walsh[2], Grace Ilevbare Okoh[3] and Damien Thompson[1*]

[1]Department of Physics, Bernal Institute, University of Limerick, V94T9PX, Ireland.

[2]Merck, Kenilworth, New Jersey 07033, United States.

[3]Merck, West Point, Pennsylvania 19486, United States.

Table of Contents

Supplementary Notes

S1. Convergence of MD simulations S4

S2. Calculation of binding energy S5

S3. Computed interaction and contact maps S6

S4. Free energy maps S7

S5. Peptide point mutations in the design of co-agonists S7

S6. MD-directed Design (MDD) of a GR co-agonist S8

S7. Activation and conformational dynamics of GRs S8

List of Figures

Fig. S1. Cartoon representation of (A) GCGR and (B) GLP-1R S10

Fig. S2. Sequence information on Glucagon and Glucagon-like Peptide-1 Receptor S11

Fig. S3. Primary sequences of peptides (both obtained through PDL and designed) were used for MD simulations to study their binding affinities with GCGR and GLP-1R. S12

Fig. S4. Cumulative average secondary structural changes as a function of simulation time in the glucagon receptor during simulations of peptide co-agonist binding to GCGR. S13

Fig. S5. Secondary structural changes in the glucagon-like peptide 1 (GLP-1) receptor S14

Fig. S6. RMSF of backbone atoms of class B1 receptors during co-agonist/GR binding dynamics S15

Fig. S7. RMSF of C-alpha atoms of class B1 receptors during co-agonist/GR binding dynamics S16

Fig. S8. RMSD of protein backbone of GRs during peptide agonist/GR binding dynamics S17

Fig. S9. The Fraction of Native Contacts (Q) from simulations of peptide agonist/GR complexes. S18

Fig. S10. Comparison of binding free energy (ΔG_{bind} in kJ/mol). S20

Fig. S11. Difference of residue-wise contribution maps for PDL co-agonist peptides binding to GCGR........ S21

Fig. S12. Difference of residue-wise contribution maps for PDL co-agonist peptides binding to GLP-1R. S22

Fig. S13. Energy contribution of PDL-peptide residues to the GCG receptor binding energy. S23

Fig. S14. Energy contribution of PDL-peptide residues to the GLP-1 receptor binding energy S24

Fig. S15. The average number of hydrogen bonds formed between co-agonist and GRs S25

Fig. S16. Schematic representation of (A) GCGR and (B) GLP-1R S26

Fig. S17. Calculated distance timelines between co-agonists and receptors S26

Fig. S18. Remote control of Glucagon & GLP-1 receptor function from the extracellular vestibule........ S27

Fig. S19. Interaction maps of (A) PDL co-agonist-GCGR and (B) PDL co-agonist-GLP-1R complexes S28

Fig. S20. Computed contact maps between residues of simulated PDL co-agonists and GCG/GLP-1 receptors. S29

Fig. S21. Modelling of Cotadutide in complex with GRs S30

Fig. S22. The primary sequence of modelled co-agonist peptides from PDL along with template peptide glucagon (endogenous ligand for GCGR) S31

Fig. S23. The primary sequence of modelled co-agonist peptides from PDL along with template peptide glucagon (endogenous ligand for GCGR) S32

Fig. S24. The primary sequence of the endogenous ligand, designed MD-guided co-agonists based on the per residue-wise decomposition energies data from PDL-peptide/GRs simulation, and reference dual-agonist peptide (Cotadutide) S33

Fig. S25. Computed interaction maps of MDD-peptide/GRs complex. S34

Fig. S26. Conformational free energy maps of agonist-bound GRs against apo-GRs S35

Fig. S27. Structural basis of glucagon and glucagon-like peptide-1 receptor co-agonist binding and activation........... S36

Fig. S28. Activation mechanism of class B1 GPCRs. S37

Fig. S29. Difference of residue-wise contribution maps of MDD co-agonist peptides binding to GCGR. S38

List of Tables

Table S1. Description of systems used for MD simulation with corresponding timescales of runs. S39

Table S2. MM/PBSA binding free energies (in kJ/mol) of different peptide agonist/GR complexes S42

Table S3. Peptide agonist binding on GCGR and GLP-1R. S44

Table S4. Contribution of important peptide residues to the binding of peptide co-agonist to GRs. S45

Table S5. Mutation points on GCG template to design selected PDL-peptide and MDD peptides S46

Table S6. The residue-wise decomposition of ΔGbind (kJ/mol) of PDL mutants compared to wild-type endogenous agonists (GCG and GLP-1).. S47

Table S7. The effect of in silico GCG mutations on GR binding affinities.. .... S48

Table S8. Co-agonist-GRs intermolecular hydrogen bonds (H-bonds) from MD simulations. S49

Table S9. Co-agonist-GRs intermolecular salt bridge during molecular dynamics simulation. S51

Table S10. Intrinsic dynamics stability statistics of all peptides used in this study S52

Supplementary Notes

S1. Convergence of MD simulations

To evaluate the convergence of MD simulations, we monitored the secondary structure elements of receptors throughout the 100 ns MD runs. We computed the timelines of cumulative average secondary structure features. Cumulative averages calculate the average property at every timestamp of simulation, such that each sliding averaged property over the time interval is computed. **Figs. S4** and **S5** show the preservation of protein secondary structures in the GCG and GLP-1 receptor throughout the simulations and provide support for the high stability of the receptor structure in the complex system between co-agonist and GRs. **Fig. S6** depicts the low Root Mean Square Fluctuation (RMSF) of GCGR and GLP-1R, respectively during the simulations with enumerated agonist peptides. RMSF, *i.e.* standard deviation of atomic position in the 100 ns trajectory after fitting to the reference "average" structure of peptide/receptor, is calculated to check the fluctuations of C-alpha atoms of receptor in individual co-agonist/GR complex in the simulations. The highly fluctuating atoms in residues in all the systems (including the apo-GRs) are in the intra- and extracellular loop regions while other regions displayed low fluctuation over time (**Fig. S7**). Since the GRs ECD consists of many mobile loops, it exhibits a larger deviation than the transmembrane domain (TMD) for both receptors. The root-mean-square deviation (RMSD) analysis allows the quantification of the degree of conformation changes that occurred during the co-agonist/GR simulations. To examine the conformational changes in GRs, we used the post-processed MD trajectories of the receptors to generate the RMSD plots (**Fig. S8**). It was noted that the GCGR backbone RMSDs exhibited deviation in the range of 0.5-2.5 Å concerning their equilibrated structure, mostly this deviation was caused due to the structural changes in flexible termini and loop regions. The intrinsic dynamics stability of the designed PDL dual co-agonist against the endogenous peptide ligand (GCG and GLP-1) during the simulation has been summarized in **Table S10**, which shows the peptide helix is very stable throughout the simulation. The peptide remains stable and intact during the simulation, as formulation development of parenteral peptide therapeutics frequently encounters aggregation challenges. Native contacts determine protein folding and stability mechanisms in atomistic simulations. It is evident from the fraction of native contacts analysis, that both the receptor in the simulations with co-agonistic peptides, reached a plateau after 50 ns (except for P_{11}/GLP-1R complex which reflects the stability after reaching ~80 ns) from the fraction of native contacts (**Fig. S9**). Fraction of native contacts, *Q(X)*[1] presents the property of retainment of contacts in the native folds of receptors as a function of simulation time, and was calculated using the following equation:

$$\boldsymbol{Q(x)} = \frac{\mathbf{1}}{\boldsymbol{N}}\sum_{(i,j)} \frac{\mathbf{1}}{\mathbf{1} + \boldsymbol{exp}\,[\boldsymbol{\beta(r_{ij}(x)} \; - \boldsymbol{\lambda r^{0}_{ij}}]} \qquad \textbf{(S1)}$$

Where, N represents the set of all pairs of heavy atoms (i, j) that are in contact if their distance is less than 6 Å and are separated by at least 3 residues. The distance between heavy atoms i and j in the conformation (x) sampled at time t is represented by $r_{ij}(x)$. The smoothing parameter, β, is taken to be 5 $Å^{-1}$, and λ is a factor that describes fluctuations when the contact is formed, with a value of 1.8, which are standard values[1].

S2. Calculation of binding energy

Exploring co-agonist and GRs interaction with MD simulations and binding free energy calculations:

Quantitative knowledge of class B1 GPCRs (glucagon and glucagon-like peptide-1 receptor)-peptide ligand binding affinities is essential in understanding molecular recognition; hence, efficient, and accurate binding free energy (ΔG_{bind}) calculations are a central goal in bioinformatics and drug design.

Molecular mechanics combined with Poisson–Boltzmann and surface area (MM/PBSA)

$$\mathbf{\Delta G_{bind} = G_{GPCR/co-agonist} - \left(G_{GPCR} + G_{co-agonist}\right)} \qquad \textbf{(S2)}$$

$G_{GPCR/co\text{-}agonist}$ represents the free energy of the peptide ligand and class B GPCRs complex, and G_{GPCR} and $G_{co\text{-}agonist}$ represent the free energies of the unbound secretin receptor and co-agonist, respectively. The free energy term G_x, where x corresponds to GPCR/co-agonist or GPCR or c-agonist and is given by,

$$\mathbf{G_x = E_{bonded} + E_{vdW} + E_{ele} + G_{polar} + G_{apolar} - TS} \qquad \textbf{(S3)}$$

where E_{bonded} comprises bond-stretch, angle-bend, torsion, and improper-dihedral energies, and E_{vdW} and E_{ele} are the van der Waals and electrostatic nonbonded interaction energies, respectively. Together, the sum of these terms makes up the vacuum molecular mechanics (MM) energy terms as shown below,

$$\mathbf{E_{MM} = E_{bonded} + E_{vdW} + E_{ele}} \qquad \textbf{(S4)}$$

while G_{polar} and G_{apolar} in Equation S3 constitute the solvation-free energies calculated using a continuum (implicit) solvation model, representing the free energy change due to transferring a solute in a vacuum to the solution. G_{polar} is the electrostatic contribution to solvation and is obtained by solving the Poisson-Boltzmann (PB) equation[2], while the G_{apolar} term is the nonpolar contribution and

is often approximated by a solvent accessible surface area (SASA) term. G_{apolar} is estimated from a linear relation to solvent-accessible surface area (SASA) as:

$$\mathbf{G_{apolar} = \gamma . SASA + b} \qquad \textbf{(S5)}$$

where γ is a coefficient set to the surface tension of the solvent and b is a fitting parameter. TS is the entropy contribution, where T is the absolute temperature and S is the configurational entropy. It should be noted that although the solvation energy change, ΔG_{solv} is a free energy term, the ΔE_{MM} does not consider the change in entropy due to binding, and hence is not a free energy term. Therefore, the total binding energy ΔG_{bind} approximates the Gibbs free energy, with entropy contributions to the overall binding free energy difference for one peptide ligand vs. another, the binding specificity, $\Delta\Delta G_{binding}$ considered minimal in the present case as both ligands both bind to the same site and are similar in size, typical of competitive native *vs.* inhibitor ligand binding to biological receptors.

Free energy calculation methods including MM/PBSA or MM/GBSA are suitable for predicting the binding free energies and evaluating the relative stabilities and binding strengths of different biomolecular complexes with a low computational cost[3, 4]. We note that the slight overall unfavorability observed in peptide-receptor affinities for a few systems (see **Table S2)** stem from larger penalties imposed by polar solvation energies (solute-water electrostatics) on solute-solute electrostatic energies estimated in a vacuum. It is important to note that a positive overall ΔG does not necessarily reflect an unfavourable binding of the peptide to the receptor[5]. The calculated binding free energy is only an estimate of the change in free energy associated with the process of ligand binding to the receptor and could be accurately evaluated from their relative binding energies (ΔΔG) highlighting the specificity of peptide binding in one system over the other (see **Fig. S10D**). Additionally, there may be other intrinsic factors such as entropic contributions and changes in the solvent environment that could affect the overall binding affinity[6].

S3. Computed interaction and contact maps

Interaction energy profiles were calculated using GROMACS tools[7]. Interdomain contact probability maps and interaction maps were generated using the CONAN contact analysis tool[8]. These interaction maps identify H-bonds, salt bridges and hydrophobic networks between the co-agonist peptide ligand and GCG/GLP-1 receptors. These maps were produced with a cut-off inter-atomic (heavy atoms) distance of 5 Å as the minimum average distance between the heavy atoms of each residue. The intermolecular interaction maps were then generated using a truncation lifetime of 0.5. The average

numbers of hydrogen bonds (H-bonds) were counted using the auxiliary GROMACS[7] module *gmx hbond* using the criteria of donor-acceptor distance ≤ 3.5 Å and hydrogen–donor–acceptor angle ≤ 30°. The choice of 3.5 Å cutoff distance includes also weaker H-bond interactions between 3.0 and 3.5 Å, which play a crucial role in determining protein secondary structure conformations[9, 10].

S4. Free energy maps

We computed 2D free energy maps of GR ligand-bound and unbound states using Root Mean Square Deviation (RMSD) of backbone atoms and radii of gyration (R_{gyr}) order parameters (**Fig. S26**), and ligand-receptor interaction energy and receptor backbone Root Mean Square Fluctuations (RMSF) (**Fig. 27**). The values for the Gibbs free energy (kJ/mol) using two order parameters were obtained using the equation:

$$\Delta G = -k_B.T.(\boldsymbol{ln}P_i - lnP_{max}) \tag{S6}$$

where P_i is the probability distribution for pairs of order parameters, and P_{max} is its maximum, such that $lnP_i - lnP$max identifies the lowest free energy point at ΔG = 0.

S5. Peptide point mutation in the design of co-agonists

Five selected PDL-peptide sequences were modelled using the experimental GCG helical peptide structure as a reference template (see **Methods**) to construct the PDL-peptides : GR complexes (P_{11}, P_{23}, P_{28}, P_{32}, and P_{35} each in complex with GCGR and GLP-1R) and the "MD-directed design" (MDD) peptides in complex with GRs (see **Table S5** for mutation points on the peptide templates) as a starting point for MD runs. Full-length GCG : GCGR and GLP-1 : GLP-1R complexes were also modelled to guide systematic mutagenesis and rationally improve co-agonist selectivity to GRs. N-terminal residues (His1 and Ser2 or Ala2, for GCGR and GLP-1R, respectively) of peptides are known to be critical for GR interaction[11] and potency[12] and mutations at these points of agonist peptides may lead to antagonistic effect[13]. Previous studies have reported that substitution at the 22nd or 23rd position in the C-terminus of the peptides leads to a lower affinity for GRs which lowers receptor activation[14-16]. Hence, we retained these N-terminus residues when designing the GR co-agonists.

Affinity and residue specificity to GRs of the designed point mutations on the PDL-peptides was compared against the native peptide endogenous ligands (see **Tables S6, S7**) by accounting for the contributions at all residue positions towards overall net ΔG_{bind}. Q20H substitution on P_{11} and P_{32}

favoured binding to GLP-1R as position 20 also increases the affinity of native GLP-1 to GLP-1R. The effect is analogous to the positively charged side chain of Lys20 in GLP-1 facilitating receptor binding[17].

S6. MD-directed Design (MDD) of a GR co-agonist

Based on the data obtained from PDL peptide/GR molecular level simulations along with endogenous ligands, we designed and tested three new GR co-agonist peptides (MDD_{GCGR}, $MDD_{GLP\text{-}1R}$, MDD_{GR}). Free energy decomposition analysis for MDD_{GR} co-agonist binding with GRs was compared against PDL-peptide (P_{32}), endogenous ligand, and reference peptides (**Fig. S27**).

S7. Activation and conformational dynamics of GRs

S7.1 Insights into the structural basis of GR activation by peptide co-agonists

Both GRs, GCGR and GLP-1R send activation signals mainly *via* the G protein subtype (G_s) class of heterotrimeric G proteins[18-20]. While a recent study[20] reports that the GCGR activation may present some aberration in G protein coupling by also binding to a different G protein subtype (G_i), in both cases, the glucagon initially binds to the same extracellular site. Briefly, extracellular binding to class B1 GPCRs triggers a conformational change that initiates heterotrimeric G protein coupling on the intracellular receptor domain. This stimulates the GPCR to transform the inactive G protein into its active form, which further enables the intracellular physiological processes. Our computational models also suggest a large degree of GR conformational dynamics that is required for the co-agonist binding and receptor activation. We note that the designed PDL and MDD co-agonist occupies the native active site cleft within the GR's 7-TM helical bundle (see **Figs. S27B**, **G**).

S7.2 Agonist binding alters the GR conformational space

We analysed the agonist-induced conformational changes in the GRs, coupled with domain-specific alteration in the transmembrane domains. By comparing the inactive (apo/unbound) state with the active (holo/dual co-agonist bound) forms/conformations of the receptors, we can propose a mechanism for the activation pathway of GRs (**Figs. S27E**, **J**). The slow kinetics leading to delayed conformational changes from inactive to the active state of GRs precludes drawing any substantial evidence of structural stability from simulations starting from an inactive state or active structure of GRs[21-23]. Hence, we explore the conformational space of GRs in unbound (apo) and bound (holo) states **(Fig. S28)**. Our model predictions are in line with the known conformational rearrangement of

the TM6 helix observed experimentally[21, 24-26]. In the inactive state of the GRs, the HETx (His-Glu-Thr-Tyr) network locks the conformation (**Fig. S18D**). By contrast, the outward conformation of the TM6 helix opens the pocket (see **Figs. Fig. S18C**, **S27B** and **S28F**).

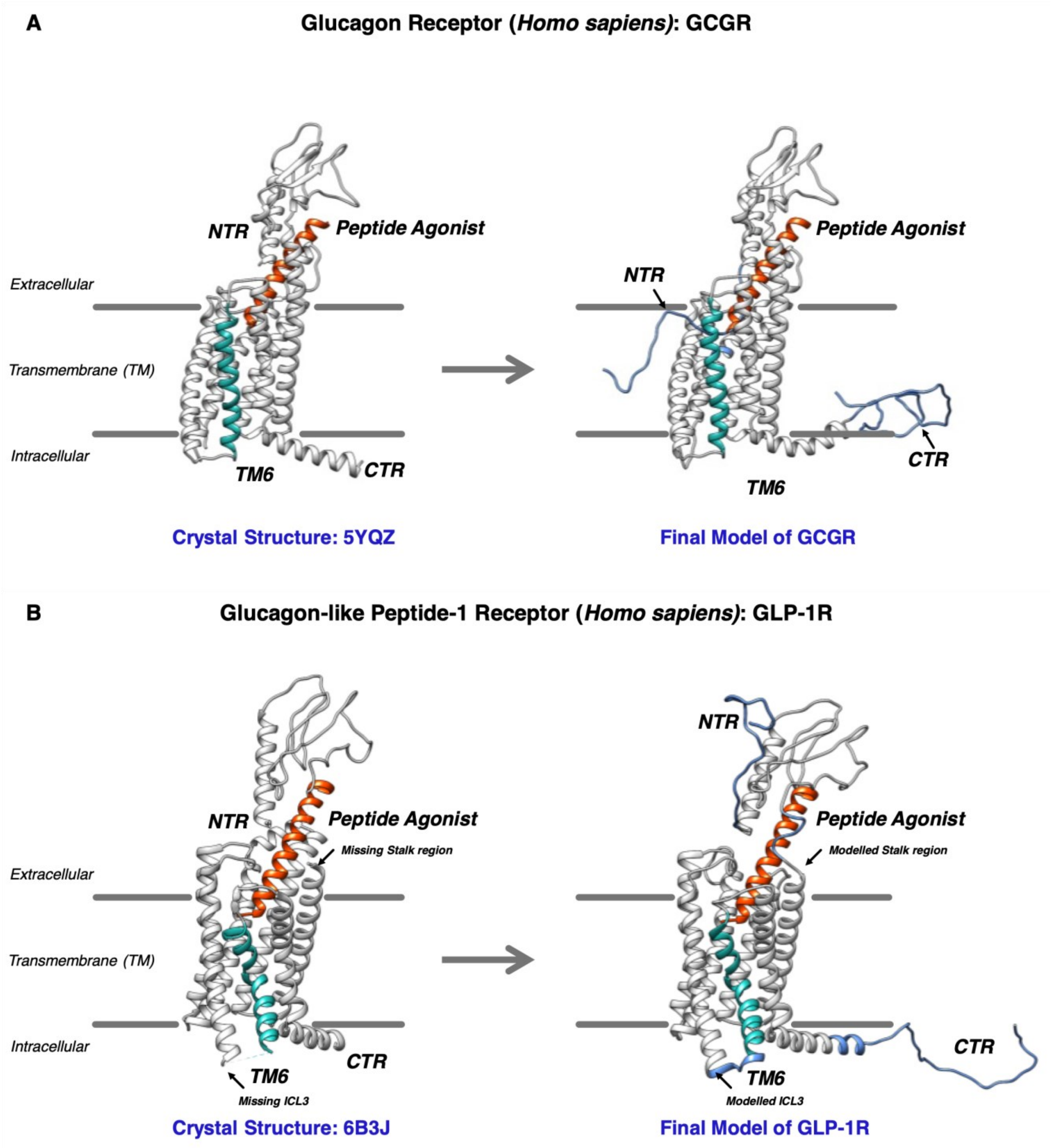

Fig. S1. Cartoon representation of (A) GCGR and (B) GLP-1R with modelled termini and inter-domain loops (shown in blue). The extracellular domain (ECD), transmembrane region (TMR) and intracellular region are depicted along with TM6 (green) and crystalized peptide agonist (orange).

A. Sequence alignment between GCGR and GLP-1R:

```
CLUSTAL O(1.2.4) multiple sequence alignment


sp|P47871|GLR_HUMAN        MPPCQPQRPLLLLLLLLA----CQPQVPSAQ-VMDFLFEKWKLYGDQCHHNLSLLPP-PT 54
sp|P43220|GLP1R_HUMAN      --MAGAPGPLRLALLLLGMVGRAGPRPQGATVSLWETVQKWREYRRQCQRSLTEDPPPAT 58
                              .    ** * ****.    . *:  .*   :   .:**: *  **::.*:  **  *

sp|P47871|GLR_HUMAN        ELVCNRTFDKYSCWPDTPANTTANISCPWYLPWHHKVQHRFVFKRCGPDGQWVRGP-RGQ 113
sp|P43220|GLP1R_HUMAN      DLFCNRTFDEYACWPDGEPGSFVNVSCPWYLPWASSVPQGHVYRFCTAEGLWLQKDNSSL 118
                           :*.******:*:****   .:.*:********   .* : .*:: *  :* *::    .

sp|P47871|GLR_HUMAN        PWRDASQCQMDGEEIEVQKEVAKMYSSFQVMYTVGYSLSLGALLLALAILGGLSKLHCTR 173
sp|P43220|GLP1R_HUMAN      PWRDLSECEESKRGERSSPEE--QLLFLYIIYTVGYALSFSALVIASAILLGFRHLHCTR 176
                           **** *:*: . .  . . *       : ::*****:**:.**::* *** *: :*****

sp|P47871|GLR_HUMAN        NAIHANLFASFVLKASSVLVIDGLLRTRYSQKIGDDLSVSTWLSDGAVAGCRVAAVFMQY 233
sp|P43220|GLP1R_HUMAN      NYIHLNLFASFILRALSVFIKDAALKWMYSTAAQQH-QWDGLLSYQDSLSCRLVFLLMQY 235
                           * ** ******:*:* **:: *. *:  **    :. . .  **     .**:. ::***

sp|P47871|GLR_HUMAN        GIVANYCWLLVEGLYLHNLLGLATLPERSFFSLYLGIGWGAPMLFVVPWAVVKCLFENVQ 293
sp|P43220|GLP1R_HUMAN      CVAANYYWLLVEGVYLYTLLAFSVLSEQWIFRLYVSIGWGVPLLFVVPWGIVKYLYEDEG 295
                            :.*** ******:**:.**.::.* *: :* **:.****.*:******.:** *:*:

sp|P47871|GLR_HUMAN        CWTSNDNMGFWWILRFPVFLAILINFFIFVRIVQLLVAKLRARQMHHTDYKFRLAKSTLT 353
sp|P43220|GLP1R_HUMAN      CWTRNSNMNYWLIIRLPILFAIGVNFLIFVRVICIVVSKLKANLMCKTDIKCRLAKSTLT 355
                           *** *.**.:* *:*:*::** :**:****::  ::*:**:*. * :** * ********

sp|P47871|GLR_HUMAN        LIPLLGVHEVVFAFVTDEHAQGTLRSAKLFFDLFLSSFQGLLVAVLYCFLNKEVQSELRR 413
sp|P43220|GLP1R_HUMAN      LIPLLGTHEVIFAFVMDEHARGTLRFIKLFTELSFTSFQGLMVAILYCFVNNEVQLEFRK 415
                           ******.***:**** ****:****  *** :* ::*****:**:****:*:*** *:*:

sp|P47871|GLR_HUMAN        RWHRWRLGKVLWEERNTSNHRASSSPGHGPPSKELQFGRGGGSQDSSAETPLAGGLPRLA 473
sp|P43220|GLP1R_HUMAN      SWERWRLEHLHIQRDSSMK------PL-KCPTSSLSSGATAGSSMYTATCQASCS----- 463
                            *.**** ::  :. .: :      *    *:..*. *  .**.  :*    : .

sp|P47871|GLR_HUMAN        ESPF 477
sp|P43220|GLP1R_HUMAN      ---- 463
```

B. Secondary structure annotation:

GCGR:

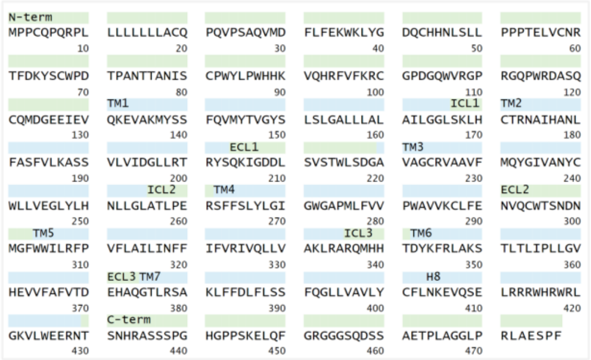

GLP-1R:

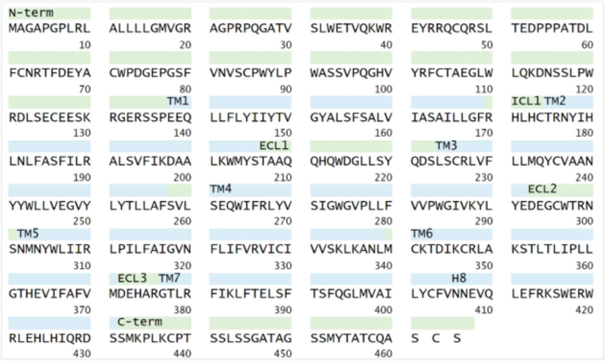

Fig. S2. Sequence information on Glucagon and Glucagon-like Peptide-1 Receptor. (A) Primary sequence alignment between GCGR and GLP-1R having a percent identity of 47.15%, (B) Secondary structure annotation of GCGR and GLP-1R.

Peptide	Receptor	1	2	3	4	5	6	7	8	9	10	11	12	13	14	15	16	17	18	19	20	21	22	23	24	25	26	27	28	29	30	31	Ligand Type
Glucagon	GCGR	H	S	Q	G	T	F	T	S	D	Y	S	K	Y	L	D	S	R	R	A	Q	D	F	V	Q	W	L	M	N	T			Endogenous
GLP-1	GLP-1R	H	**A**	**E**	G	T	F	T	S	D	**V**	S	**S**	Y	L	**E**	**G**	**Q**	**A**	A	**K**	**E**	F	**I**	**A**	W	L	**V**	**K**	**G**	**R**	**G**	Endogenous
P_{11}	GRs	H	S	Q	G	T	F	T	S	D	Y	S	K	Y	L	D	S	R	R	A	**H**	D	F	V	Q	W	L	**L**	N	T			PDL-obtained
P_{23}	GRs	H	S	Q	G	T	F	T	S	D	Y	S	K	Y	L	D	**W**	R	R	A	Q	D	F	V	Q	W	L	**Q**	N	T			PDL-obtained
P_{28}	GRs	H	S	Q	G	T	F	T	S	D	Y	S	K	Y	L	D	S	R	R	A	Q	D	F	V	**D**	W	L	**I**	N	**S**			PDL-obtained
P_{32}	GRs	H	S	Q	G	T	F	T	S	D	Y	S	K	Y	L	D	**M**	**Q**	R	A	**H**	D	F	V	Q	W	L	M	N	T			PDL-obtained
P_{35}	GRs	H	S	Q	G	T	F	T	S	D	Y	S	K	Y	L	D	S	R	R	A	Q	D	F	V	Q	W	L	**L**	**D**	**S**			PDL-obtained
MDD_{GLP-1R}	GRs	H	S	Q	G	T	F	T	S	D	Y	S	K	Y	L	D	**W**	**Q**	R	A	Q	D	F	V	**D**	W	L	M	**D**	T			Designed
MDD_{GCGR}	GRs	H	S	Q	G	T	F	T	S	D	Y	S	K	Y	L	D	**M**	R	R	A	Q	D	F	V	**D**	W	L	M	**D**	T			Designed
MDD_{GR}	GRs	H	S	**E**	G	T	F	T	S	D	Y	S	K	Y	L	**E**	**W**	**Q**	R	A	Q	D	F	V	**D**	W	L	M	**D**	T	**R**	**G**	Designed
Cotadutide	GRs	H	S	Q	G	T	F	T	S	D	**K**	S	**E**	Y	L	D	S	**E**	R	A	**R**	D	F	V	**A**	W	L	**E**	**A**	**G**	**G**		Reference

Fig. S3. Primary sequences of peptides (both obtained through PDL and designed) were used for MD simulations to study their binding affinities with GCGR and GLP-1R. The endogenous peptide ligands are GCG and GLP-1, the PDL peptides are P_{11}, P_{23}, P_{28}, P_{32} and P_{35}, and the MD-guided designed co-agonists are named MDD_{GCGR}, MDD_{GLP-1R} and MDD_{GR}, which are rationally designed based on the residue-wise binding affinities from the PDL-peptide/GRs MD simulations, and reference dual-agonist peptide (Cotadutide); all the mutation points are colour-coded on glucagon template where red residue indicates mutation on glucagon template. Colour-coding in blue indicates C-terminal residue insertions on the peptide sequence of the glucagon template. All the enlisted peptides studied with both receptors (GCGR and GLP-1R, together named as GRs) except the endogenous ligands, which are only simulated with their respective receptors GCGR and GLP-1R, respectively. All simulated agonist peptides modelled with C-terminal carboxamide ($CONH_2$) as uncharged C-terminal amide end more closely mimic the native protein and hence increase the biological activity of peptide[27].

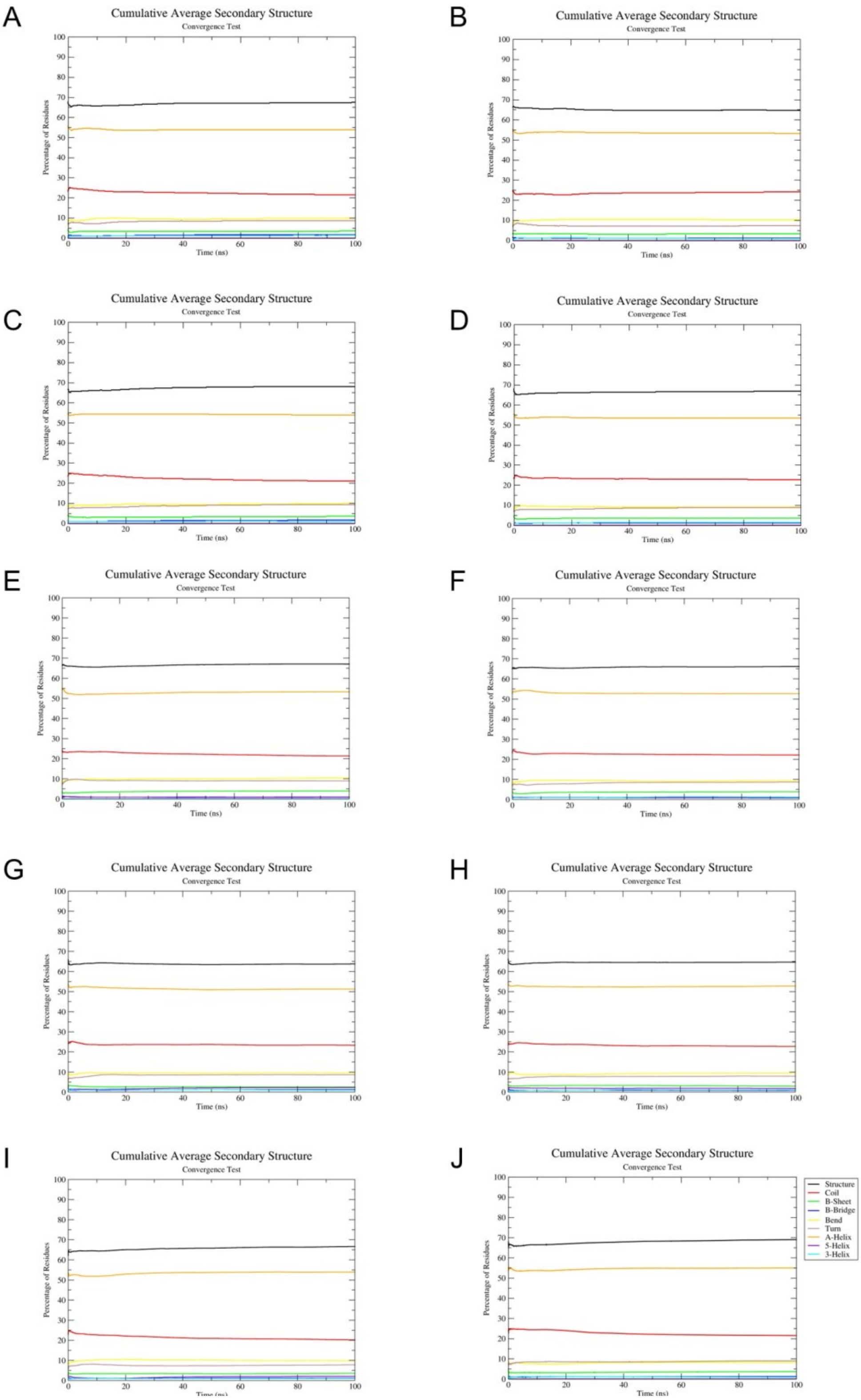

Fig. S4. Cumulative average secondary structural changes in the glucagon receptor throughout simulations of peptide co-agonist/GCGR complex: (A) P11 co-agonist-bound GCGR, (B) P23 co-agonist-bound GCGR, (C) P28 co-agonist-bound GCGR, (D) P32 co-agonist-bound GCGR, (E) P35 co-agonist-bound GCGR, (F) MDDGCGR co-agonist-bound GCGR, (G) MDDGLP-1R co-agonist-bound GCGR, (H) MDDGR co-agonist-bound GCGR, (I) Glucagon agonist-bound GCGR and (J) Cotadutide-bound GCGR.

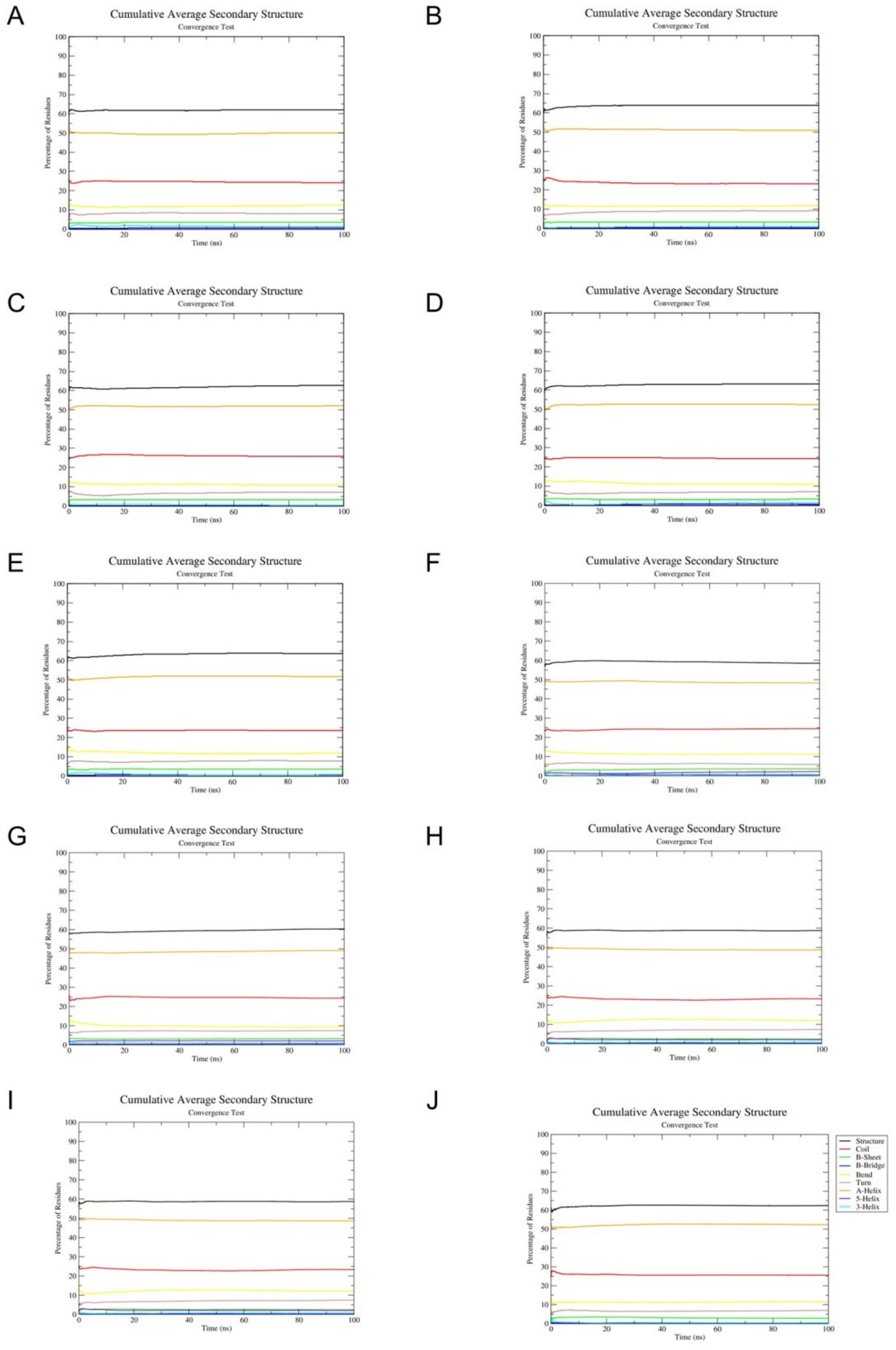

Fig. S5. Secondary structural changes in the glucagon-like peptide 1 (GLP-1) receptor throughout simulations of peptide co-agonist/GLP-1R complex: (A) P_{11} co-agonist-bound GLP-1R, (B) P_{23} co-agonist-bound GLP-1R, (C) P_{28} co-agonist-bound GLP-1R, (D) P_{32} co-agonist-bound GLP-1R, (E) P_{35} co-agonist-bound GLP-1R, (F) MDD_{GCGR} co-agonist-bound GLP-1R, (G) $MDD_{GLP\text{-}1R}$ co-agonist-bound GLP-1R, (H) MDD_{GR} co-agonist-bound GLP-1R, (I) GLP-1 agonist-bound GLP-1R and (J) Cotadutide-bound GLP-1R.

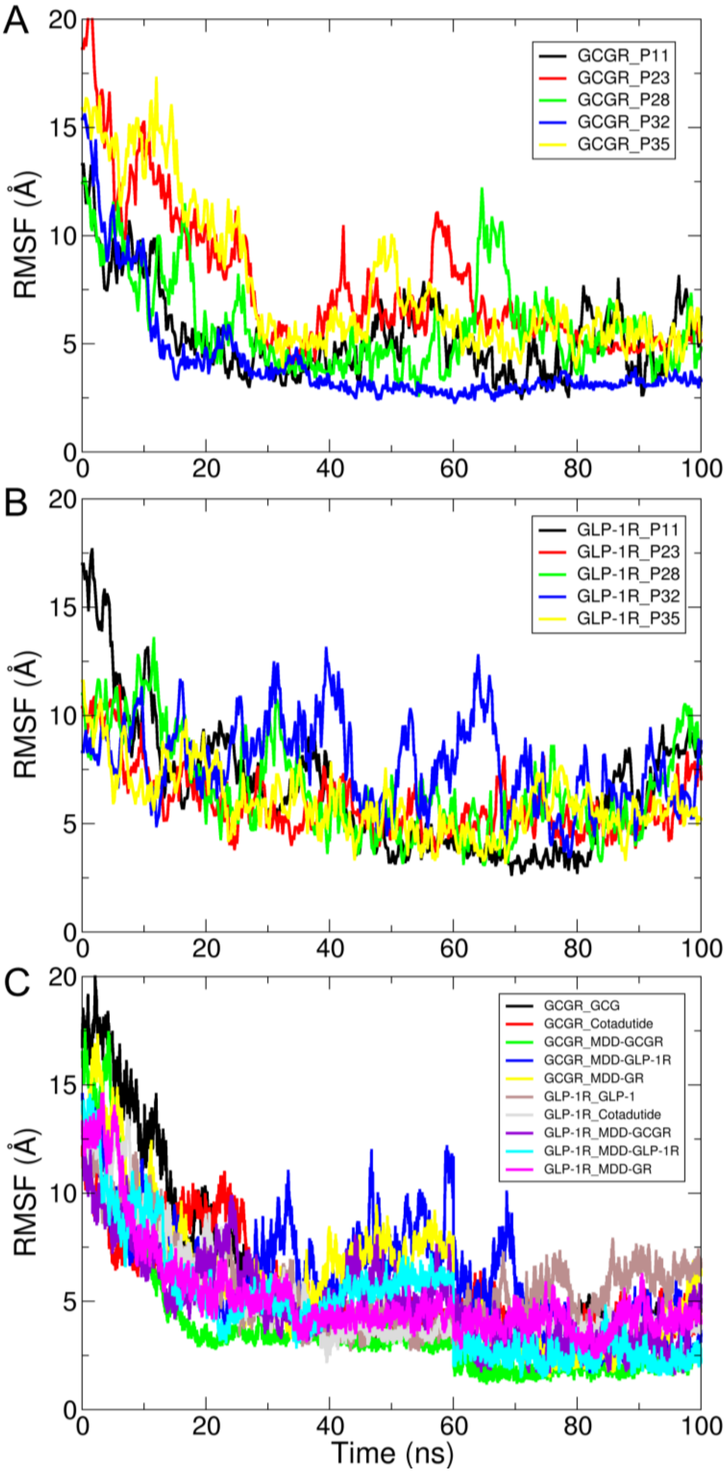

Fig. S6. Root mean square fluctuations (RMSF) of backbone atoms of class B1 receptors in individual co-agonist/GR over the 100 ns simulations at 298 K: (A) PDL co-agonist/GCGR complex, (B) PDL co-agonist/GLP-1R complex and (C) RMSF of backbone atoms of glucagon and glucagon-like peptide-1 receptor in individual agonist/GRs complex compared against endogenous ligand/GRs system.

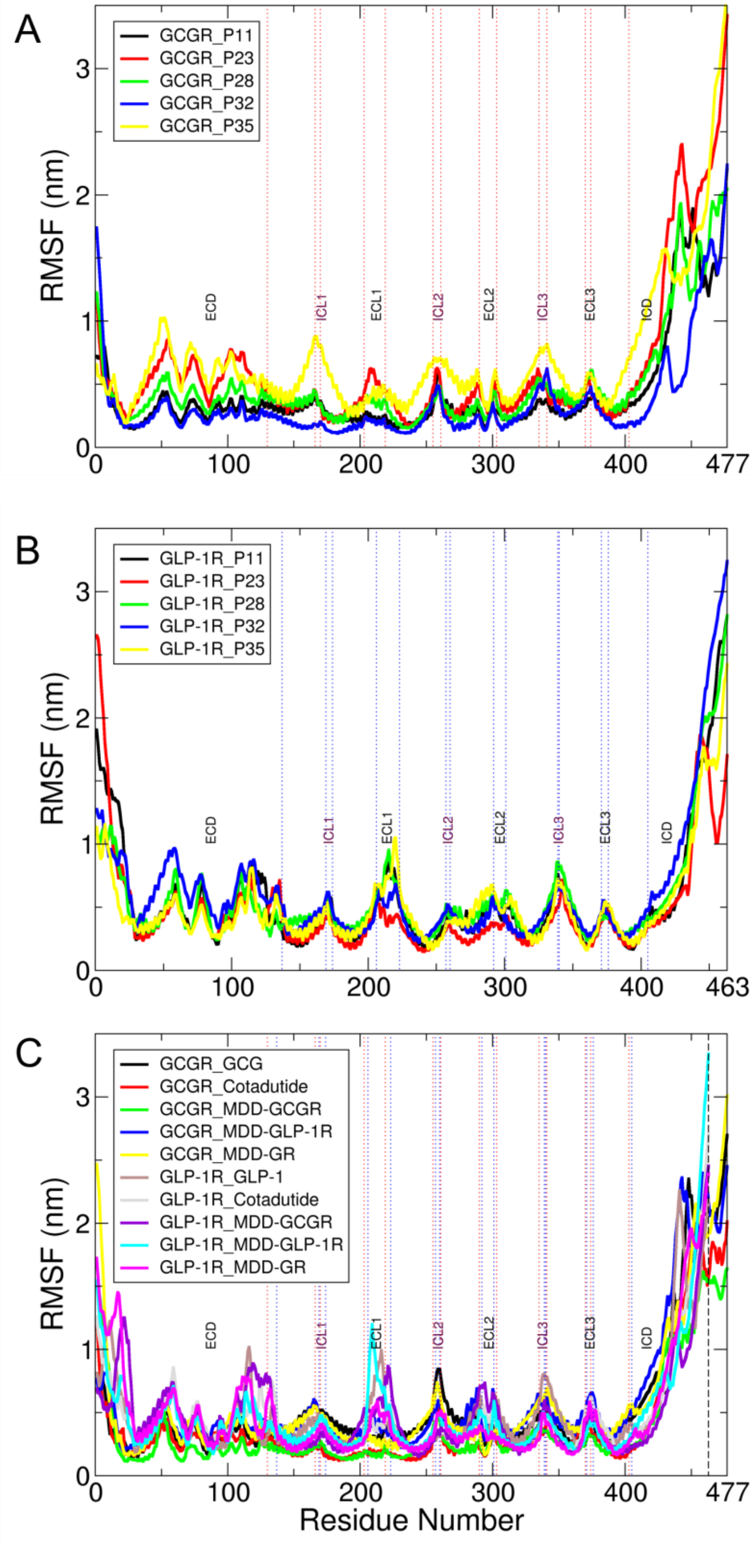

Fig. S7. Root mean square fluctuations (RMSF) of C-alpha atoms of class B1 receptors in individual co-agonist/GR in the simulations at 298 K: (A) PDL co-agonist/GCGR complex, (B) PDL co-agonist/GLP-1R complex and (C) RMSF of C-alpha atoms of glucagon and glucagon-like peptide-1 receptor in individual agonist/GRs complex compared against endogenous ligand/GRs system.

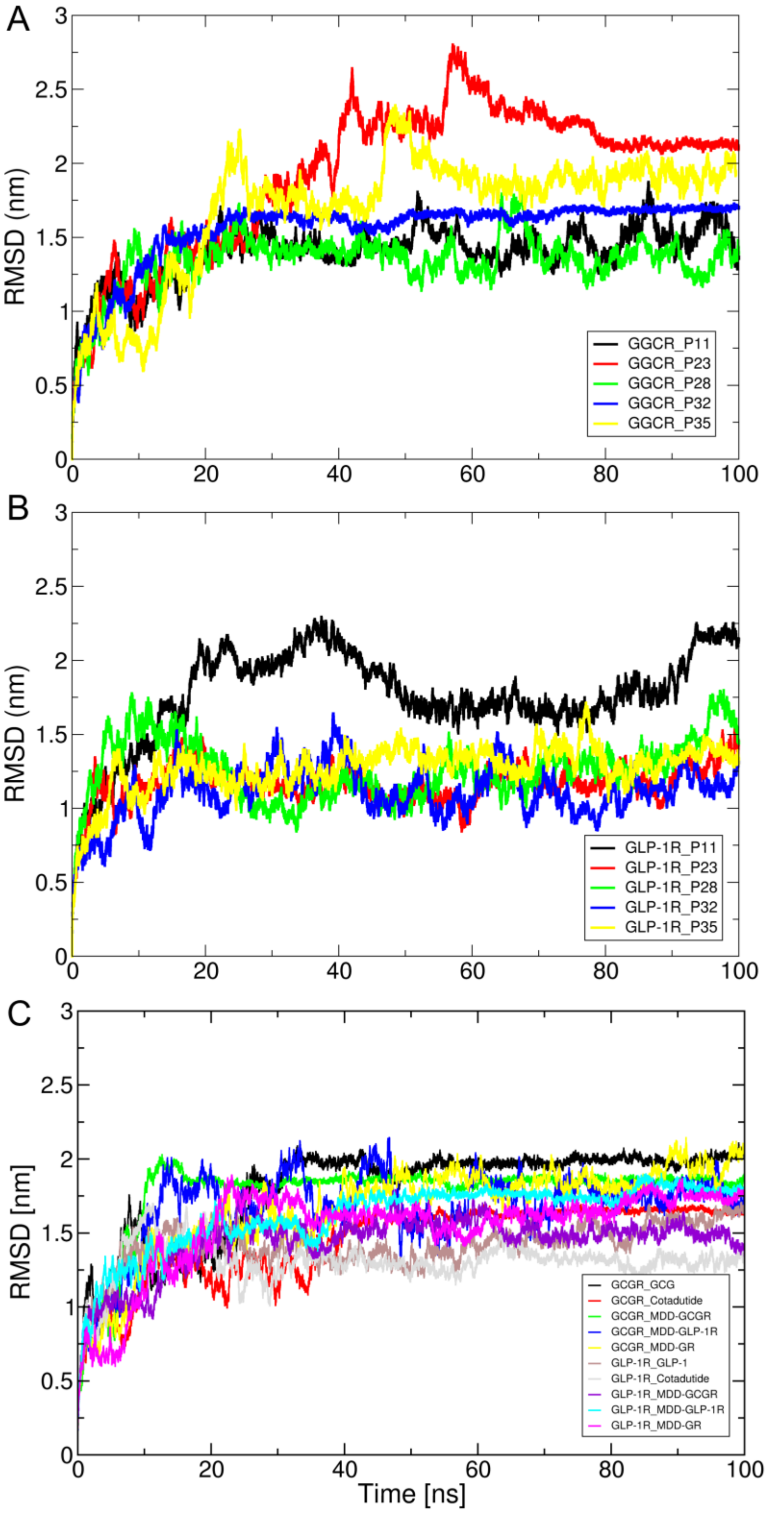

Fig. S8. Root mean square deviation (RMSD) of protein backbone of GRs in simulations of peptide agonist/GR complex. The reference structure was taken from a 100 ps "isothermal-isobaric" ensemble equilibrium simulation: (A) PDL co-agonist/GCGR complex, (B) PDL co-agonist/GLP-1R complex and (C) RMSD of protein backbone of GCGR and GLP-1R in simulations of different agonist/GRs complex compared against endogenous ligand/GRs system.

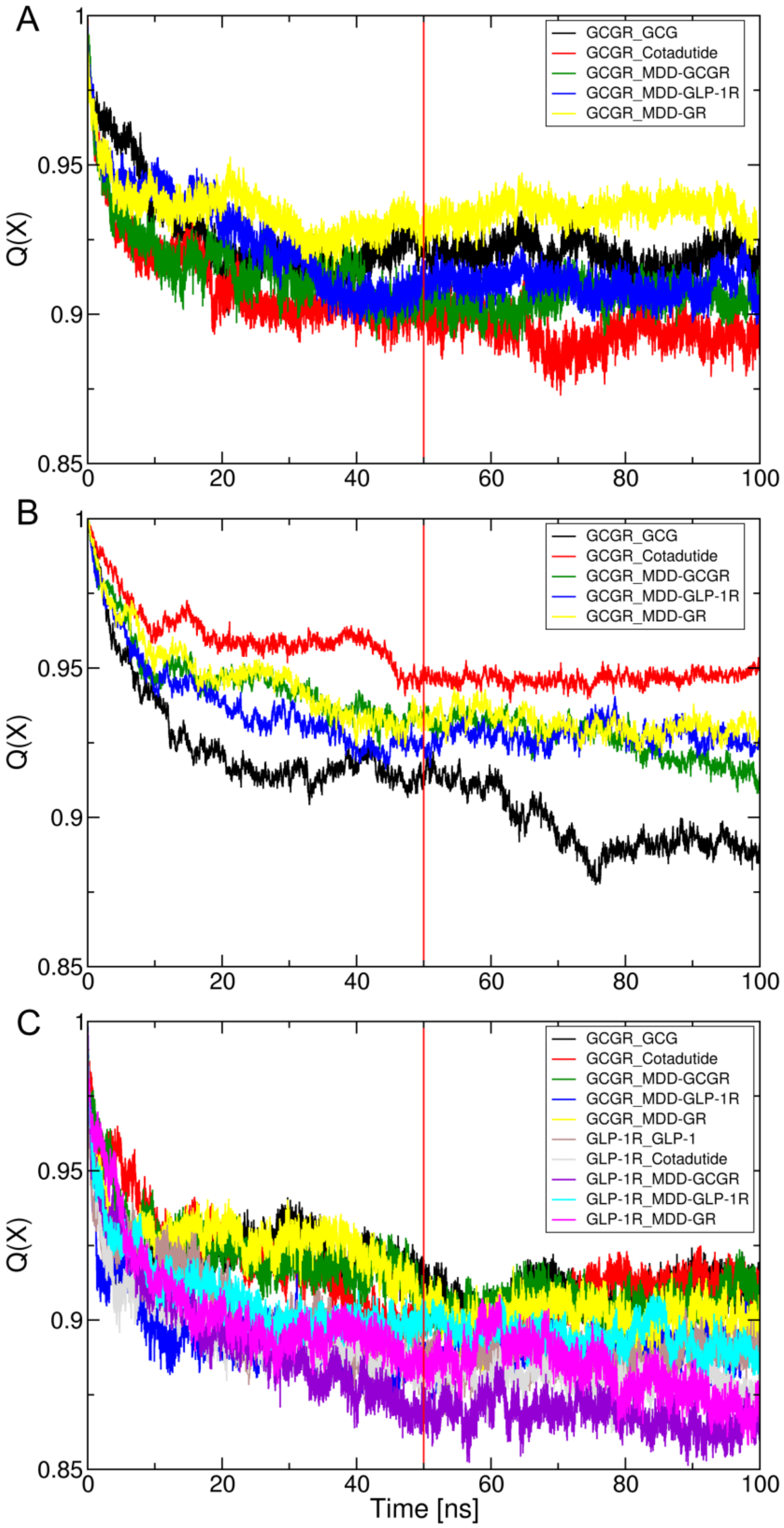

Fig. S9. The Fraction of Native Contacts (Q) for class B1 receptors in simulations of peptide agonist/GR complex: (A) for GCGR in simulations of PDL co-agonist/GCGR complex, (B) for GLP-1R in simulations of PDL co-agonist/GLP-1R complex and (C) for GCGR and GLP-1R in simulations of designed agonist/GRs complex compared against endogenous ligand/GRs system.

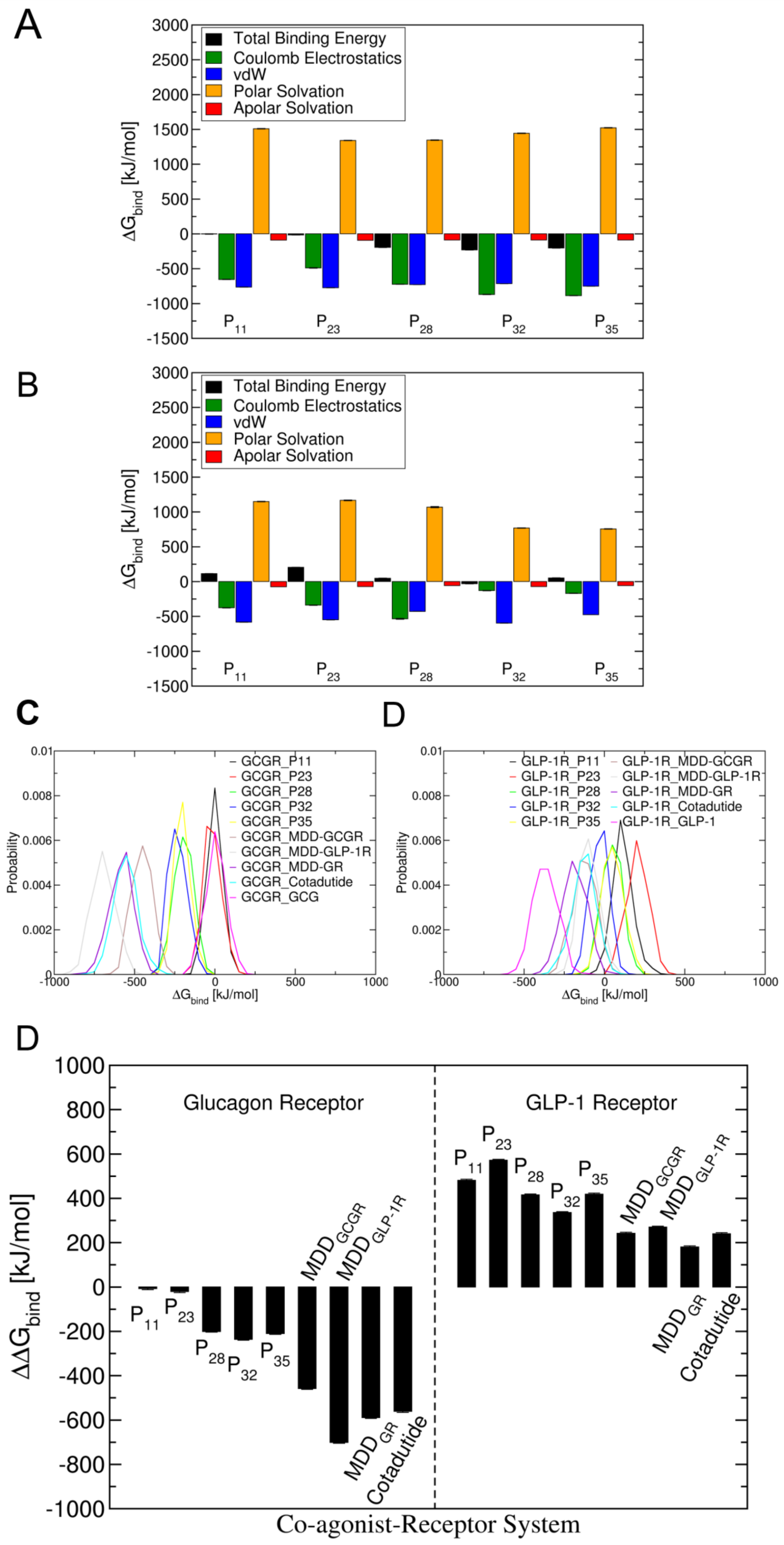
A
B
Total Binding Energy
Coulomb Electrostatics
vdW
Polar Solvation
Apolar Solvation
ΔG_bind [kJ/mol]
P11 P23 P28 P32 P35
C
D
GCGR_P11
GCGR_P23
GCGR_P28
GCGR_P32
GCGR_P35
GCGR_MDD-GCGR
GCGR_MDD-GLP-1R
GCGR_MDD-GR
GCGR_Cotadutide
GCGR_GCG
GLP-1R_P11
GLP-1R_P23
GLP-1R_P28
GLP-1R_P32
GLP-1R_P35
GLP-1R_MDD-GCGR
GLP-1R_MDD-GLP-1R
GLP-1R_MDD-GR
GLP-1R_Cotadutide
GLP-1R_GLP-1
Probability
ΔG_bind [kJ/mol]
D
Glucagon Receptor
GLP-1 Receptor
MDD GCGR
MDD GLP-1R
MDD GR
Cotadutide
ΔΔG_bind [kJ/mol]
Co-agonist-Receptor System

Fig. S10. Comparison of binding free energy (ΔG_{bind} in kJ/mol) (A) PDL co-agonists/GCGR, (B) PDL co-agonists/GLP-1R. The average total free energies (Total) for the last 50 ns further decomposed into Coulomb electrostatics, van der Waals (vdW), polar and apolar (non-polar), (C) Probability distribution of binding free energy calculated for peptide agonists/GCGR complex and peptide agonists/GLP-1R complex, (D) relative binding energies ($\Delta\Delta G_{bind}$ in kJ/mol) of the modelled peptide against endogenous ligands (glucagon and GLP-1).

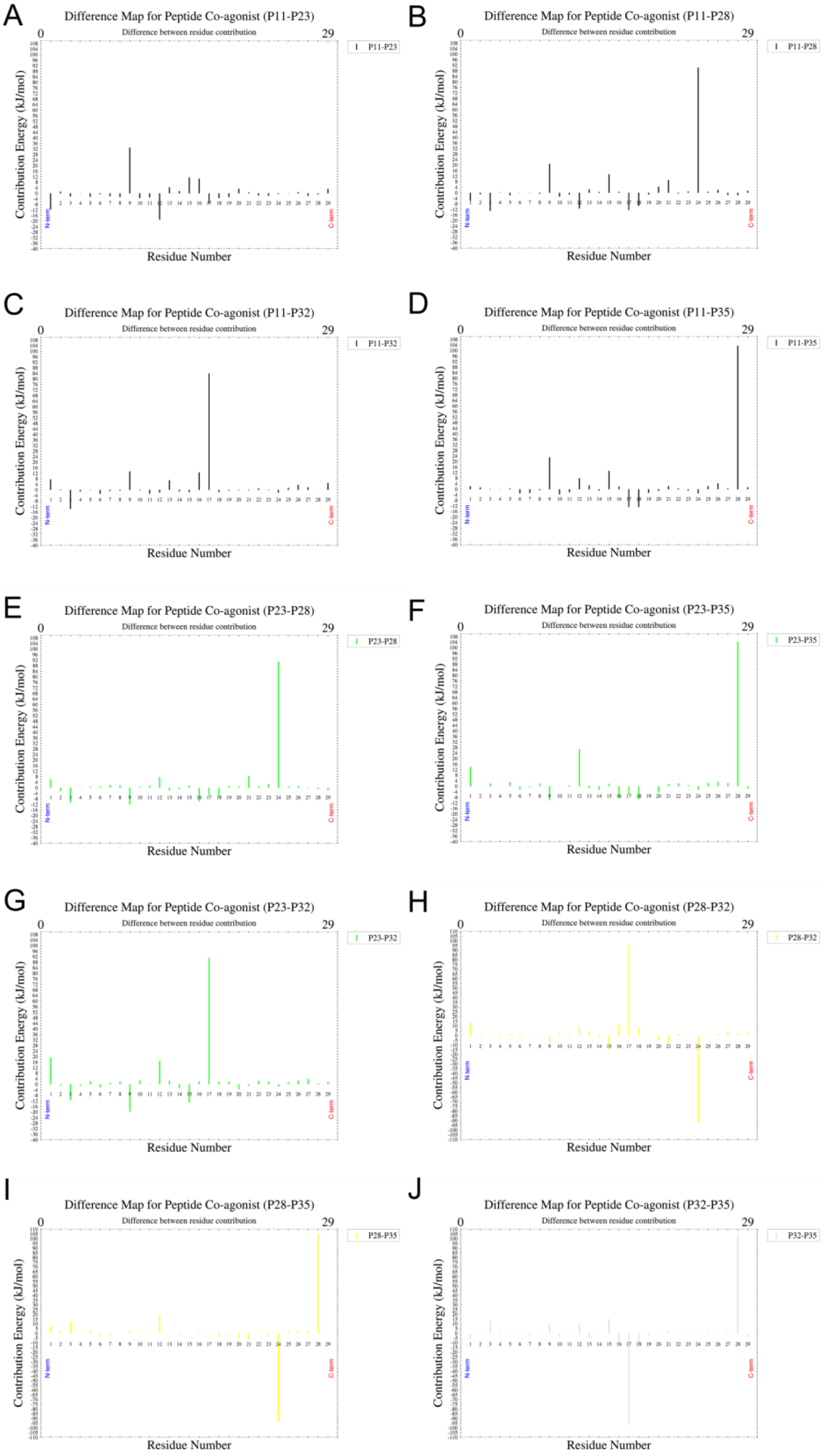

Fig. S11. Difference of residue-wise contribution maps for PDL co-agonist peptides binding to GCGR: (A) P_{11} relative to P_{23}, (B) P_{11} relative to P_{28}, (C) P_{11} relative to P_{32}, (D) P_{11} relative to P_{35}, (E) P_{23} relative to P_{28}, (F) P_{23} relative to P_{32}, (G) P_{23} relative to P_{35}, (H) P_{28} relative to P_{32}, (I) P_{28} relative to P_{35}, and (J) P_{32} relative to P_{35}.

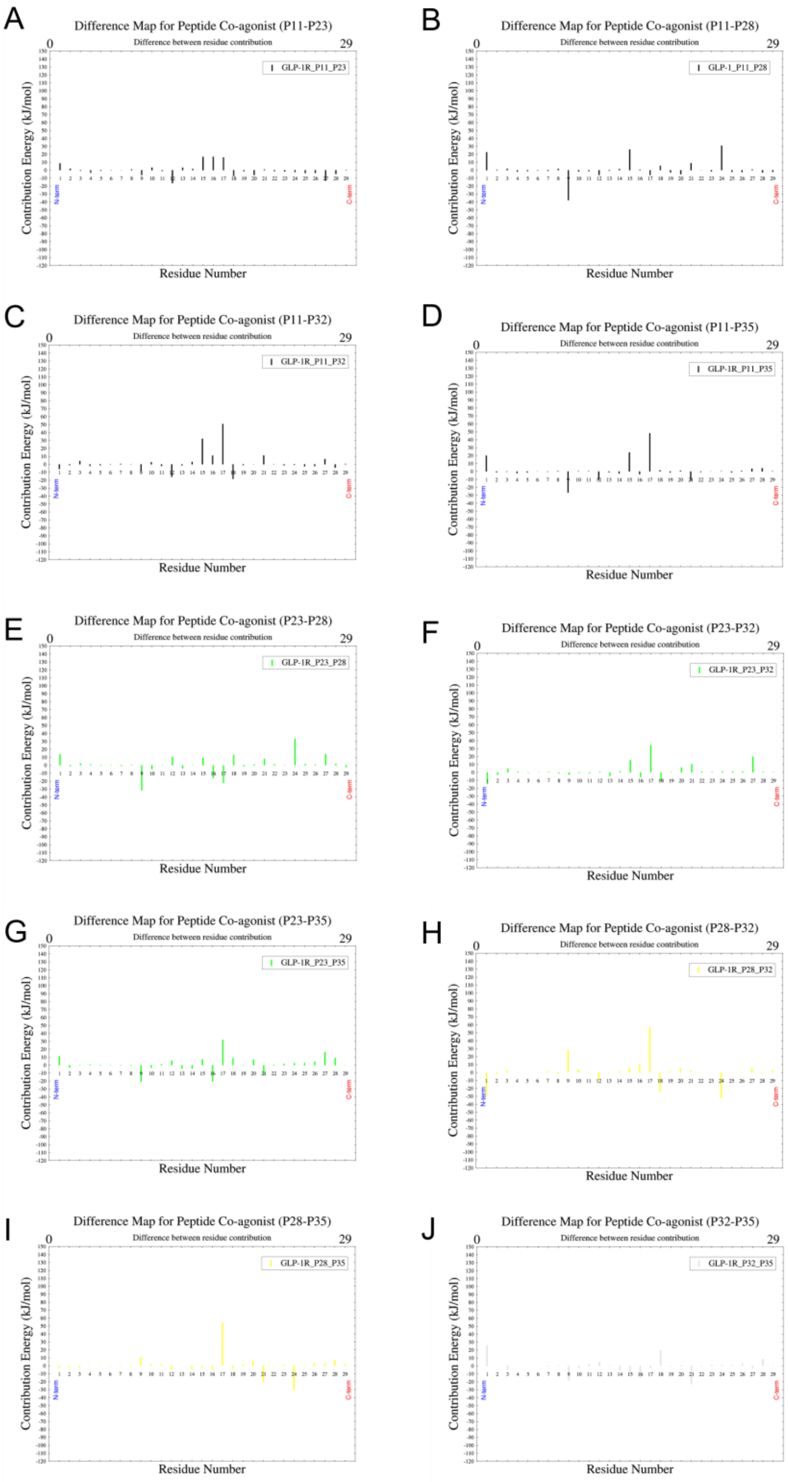

Fig. S12. Difference of residue-wise contribution maps for PDL co-agonist peptides binding to GLP-1R: (A) P_{11} relative to P_{23}, (B) P_{11} relative to P_{28}, (C) P_{11} relative to P_{32}, (D) P_{11} relative to P_{35}, (E) P_{23} relative to P_{28}, (F) P_{23} relative to P_{32}, (G) P_{23} relative to P_{35}, (H) P_{28} relative to P_{32}, (I) P_{28} relative to P_{35}, and (J) P_{32} relative to P_{35}.

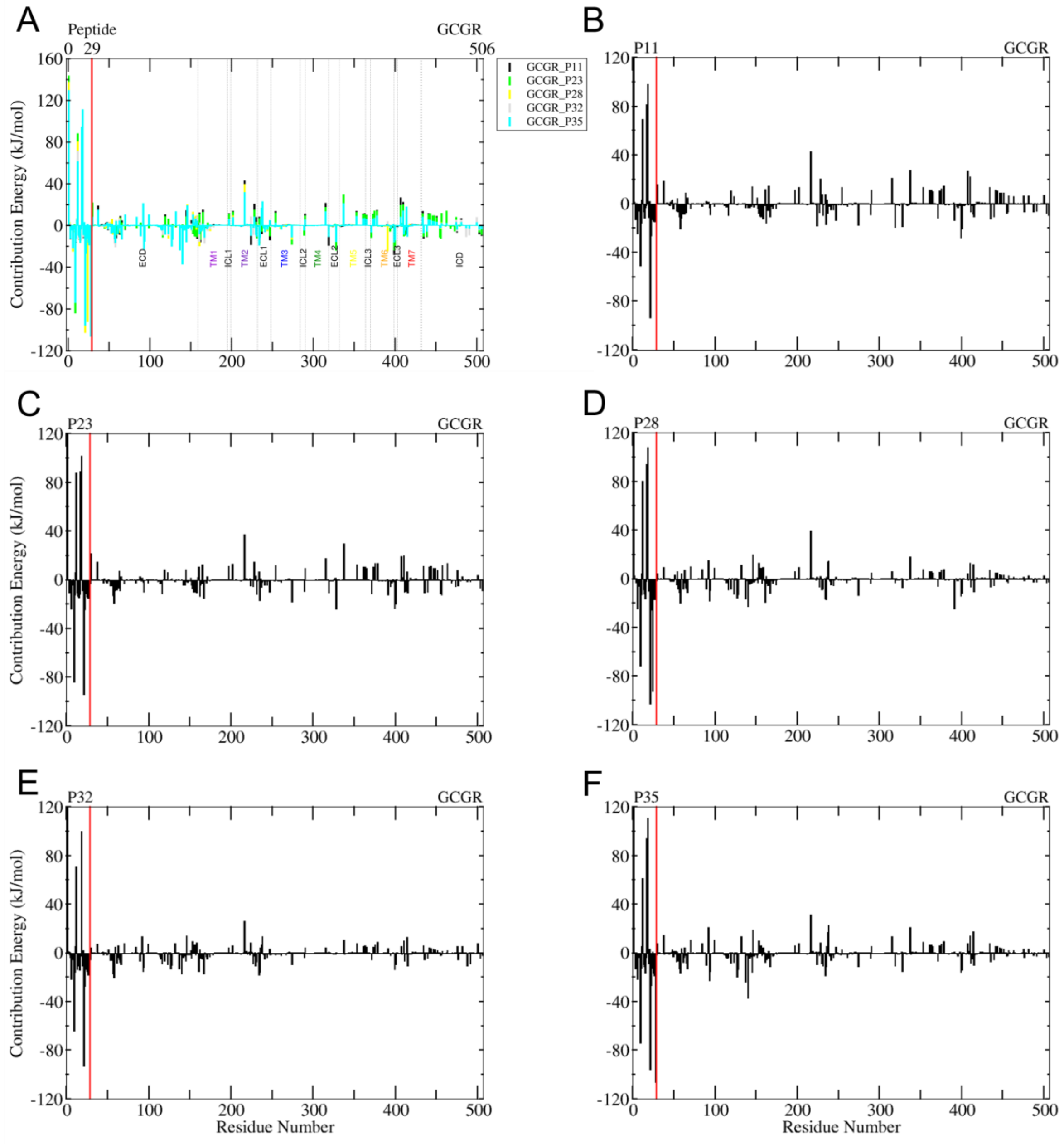

Fig. S13. Contribution of PDL-peptide residues to the GCG receptor binding energy (free energy decomposition analysis): (A) Superimposed free energy decomposition plot compares the contribution of residues in terms of binding energy for PDL co-agonists and glucagon receptor complexes (Black-P_{11}/GCGR, Green-P_{23}/GCGR, Yellow- P_{28}/GCGR, Grey- P_{32}/GCGR, Blue-P_{35}/GCGR), (B) P_{11}/GCGR, (C) P_{23}/GCGR, (D) P_{28}/GCGR, (E) P_{32}/GCGR, (F) P_{35}/GCGR.

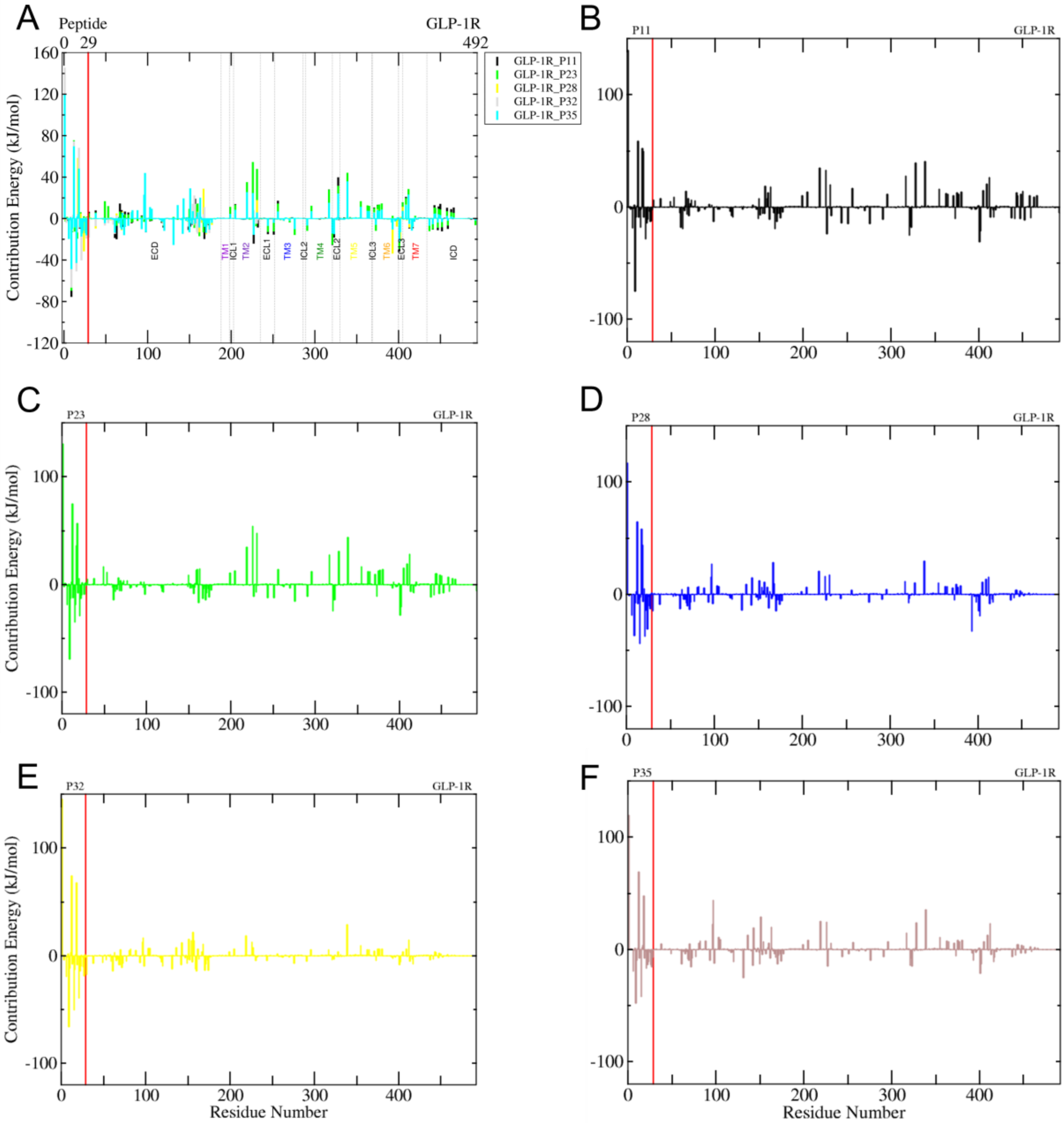

Fig. S14. Contribution of PDL-peptide residues to the GLP-1 receptor binding energy (free energy decomposition analysis): (A) Superimposed free energy decomposition plot compares the contribution of residues in terms of binding energy for PDL co-agonists and GLP-1 receptor complexes (Black-P11/GLP-1R, Green-P23/GLP-1R, Yellow- P28/GLP-1R, Grey- P32/GLP-1R, Blue- P35/GLP-1R), (B) P11/GLP-1R, (C) P23/GLP-1R, (D) P28/GLP-1R, (E) P32/GLP-1R, (F) P35/GLP-1R.

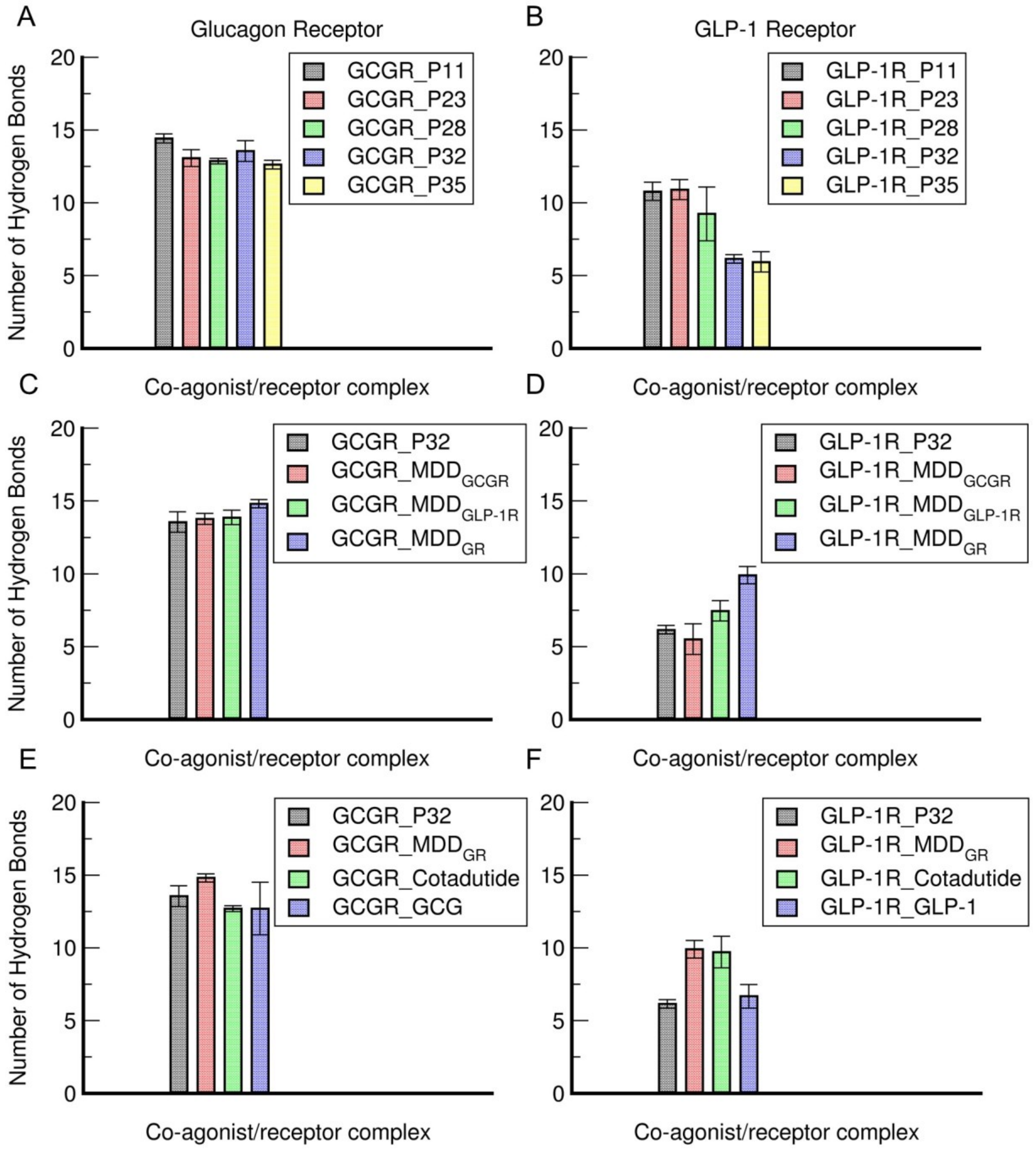

Fig. S15. The average number of hydrogen bonds formed within the period of the last 50 ns of simulations of co-agonist with GRs. The hydrogen bond length and angle cut-offs used in this analysis were 3.5 Å and 30°, respectively.

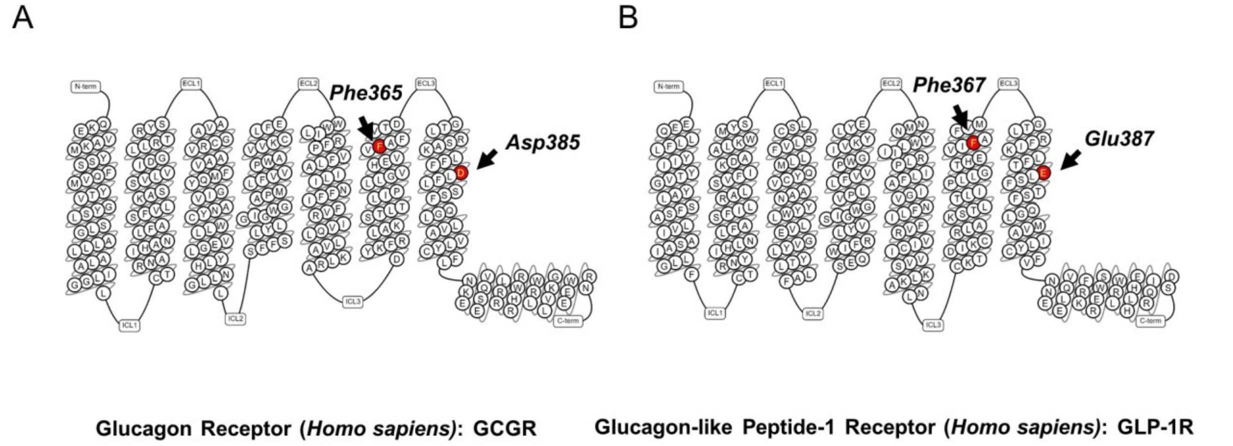

Fig. S16. Schematic representation of (A) GCGR and (B) GLP-1R. The residues (Phe365$_{TM6}$ and Asp385$_{TM7}$ for GCGR, and Phe367$_{TM6}$ and Glu387$_{TM7}$ of GLP-1R) play important roles in co-agonist binding by H-bond formation with Ser2 of co-agonist (P_{32}) and receptor activation are highlighted.

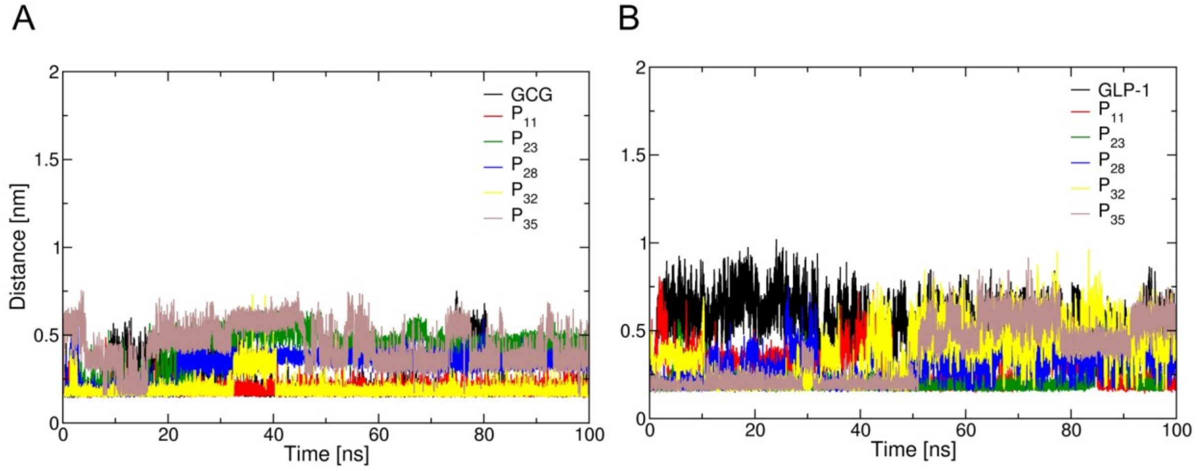

Fig. S17. Calculated distance timelines between (A) penultimate residue (Ser2) of peptide co-agonist and Asp385$_{TM7}$ of GCGR (Ser2:OG-HG1---OD2:Asp385$_{TM7}$), and (B) penultimate residue (Ser2) of peptide co-agonist and Glu387$_{TM7}$ of GLP-1R (Ser2:N-HN---OE1:Glu387$_{TM7}$).

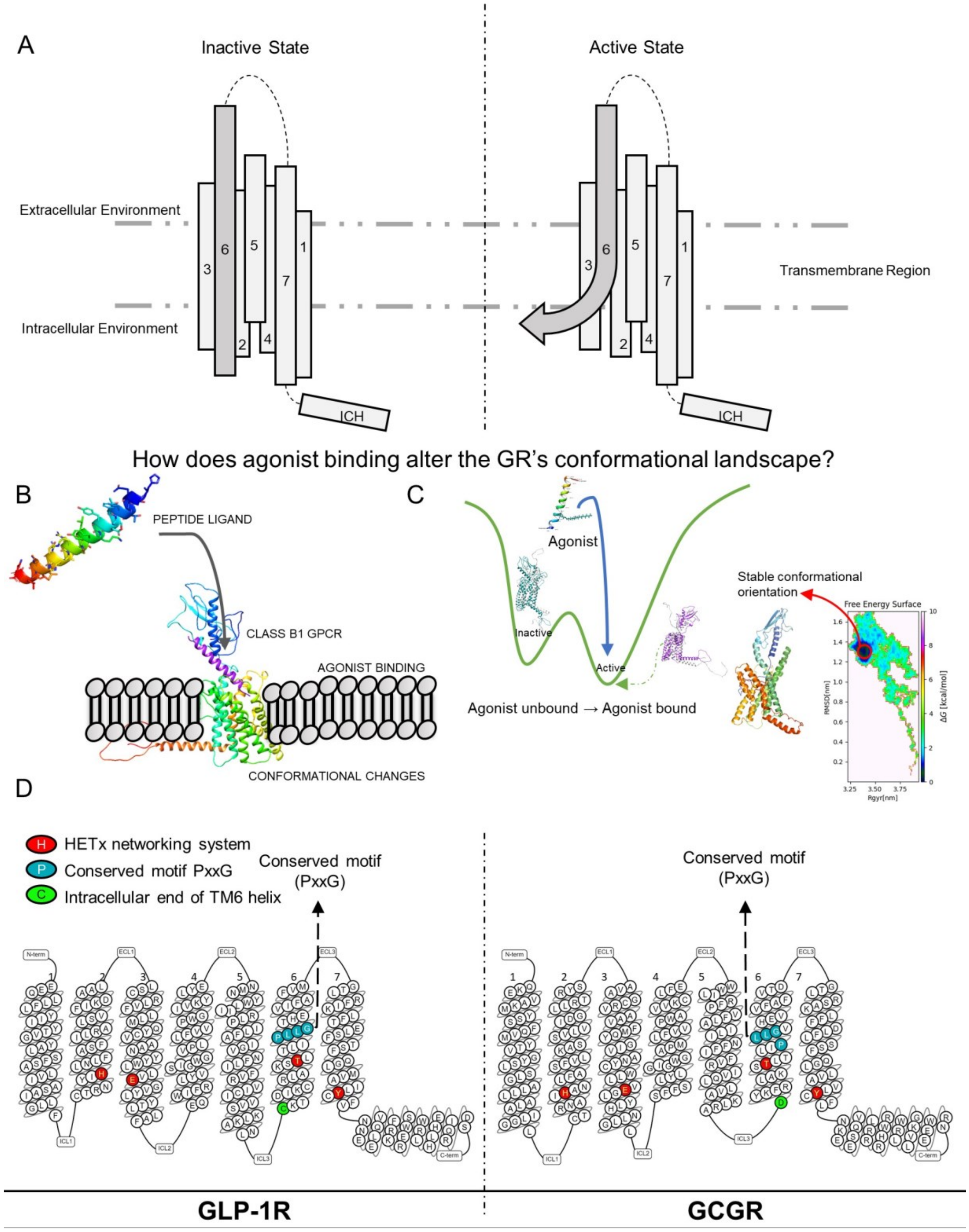

Fig. S18. Remote control of Glucagon & GLP-1 receptor function from the extracellular vestibule: (A) Schematics of representative conformations of the active and inactive state of GRs, (B) Schematics of peptide co-agonist targeting GR, (C) assessing the GR's conformational landscape and (D) Schematic diagram of GCG/GLP-1 receptor, showing residues of highly conserved PxxG motif and HETx network that provides a stable outward conformation of TM VI by structural rearrangements[28].

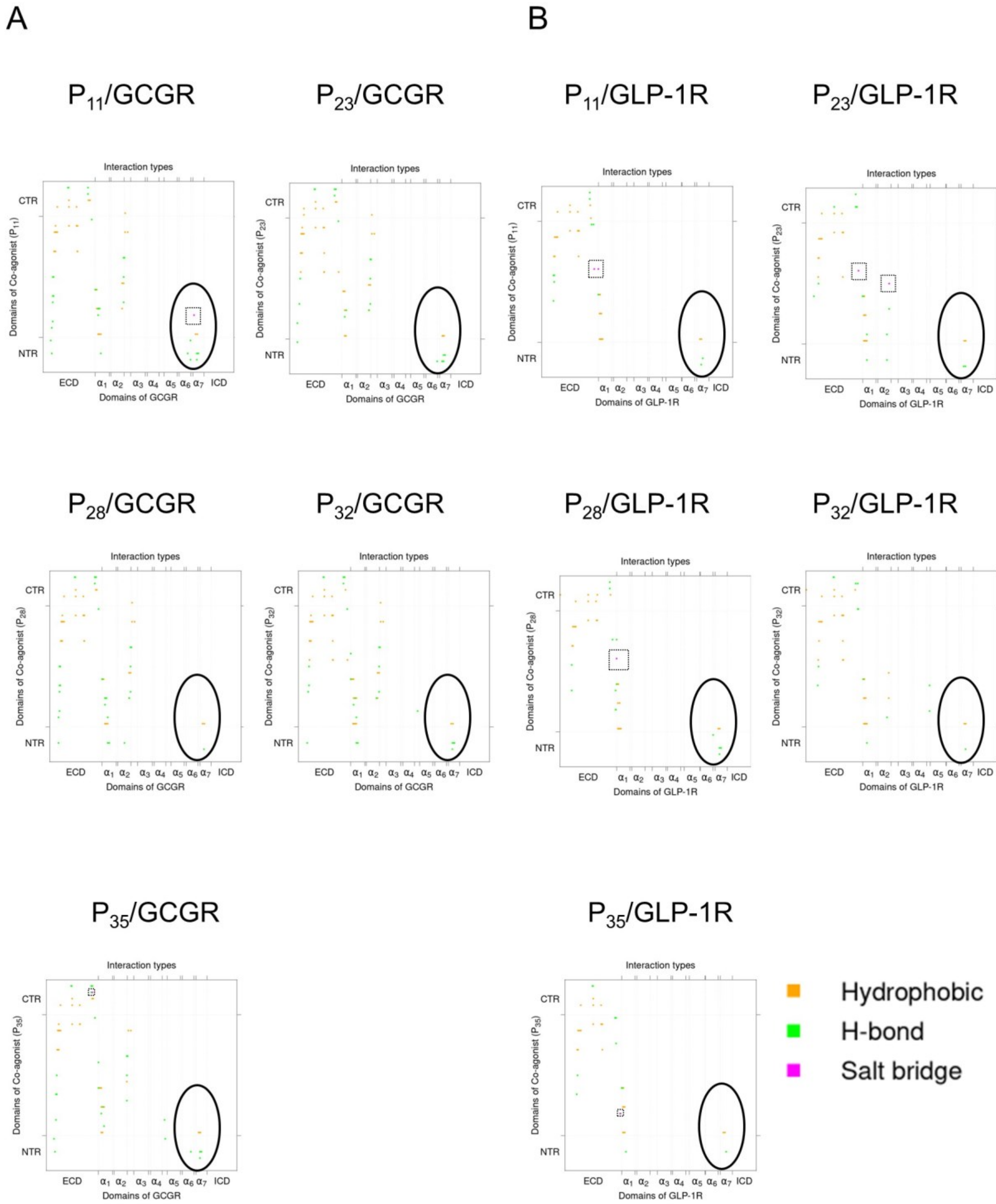

Fig. S19. Interaction maps of (A) PDL co-agonist-GCGR and (B) PDL co-agonist-GLP-1R complexes were computed from the last 50 ns of simulations using the CONAN tool. Specific interactions in the maps are coloured: orange - hydrophobic, green - hydrogen bond, and purple - salt bridge. The Circled area shows the interactions between the NTR of peptides and ECL3 connecting the helix TM6 and TM7. Salt bridge interactions are circled by broken black lines.

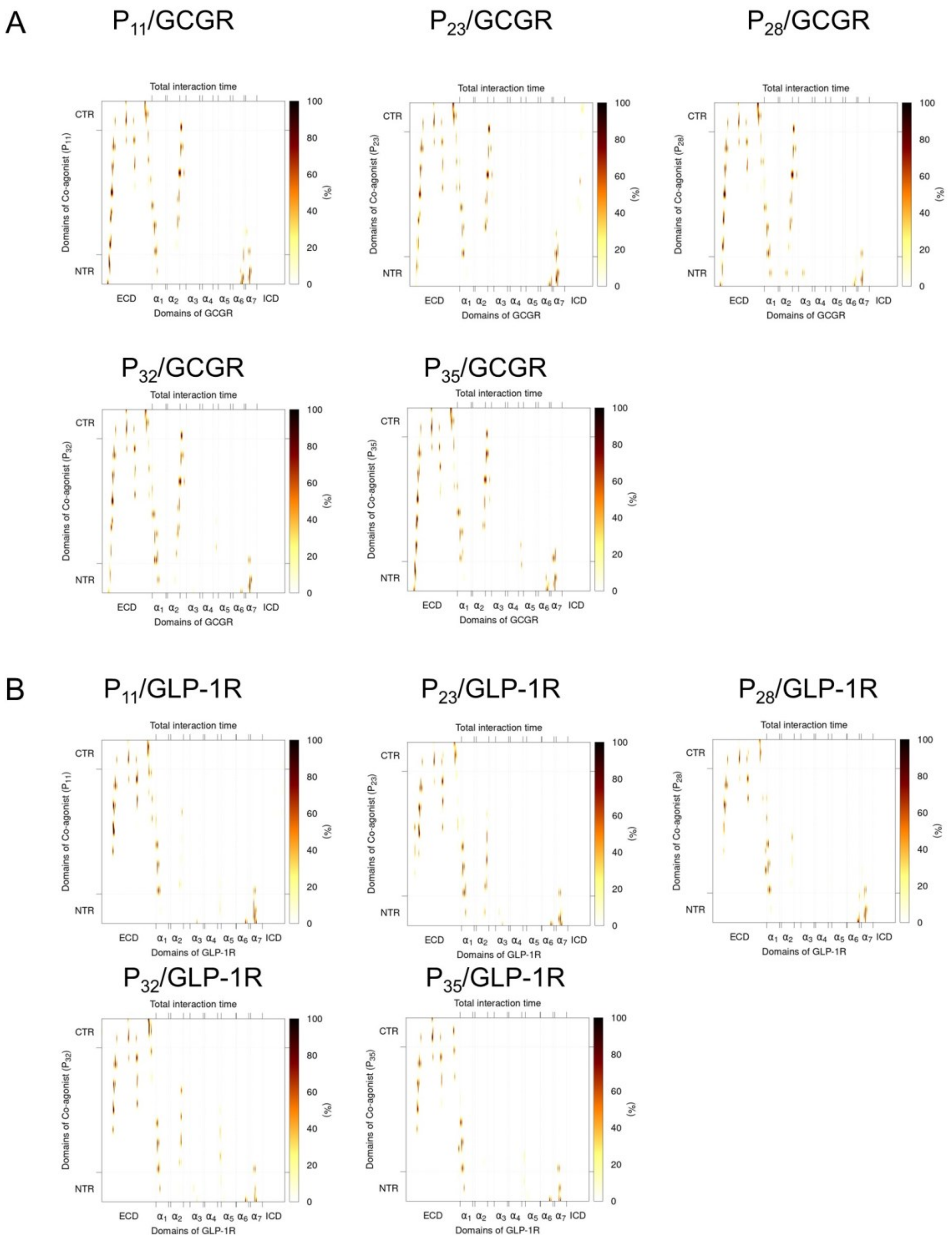

Fig. S20. Computed contact maps (frequency, %) between residues of different simulated PDL co-agonist and GCG/GLP-1 receptors: (A) contact maps of PDL co-agonist-GCGR and (B) contact maps of PDL co-agonist-GLP-1R complexes computed from the last 50 ns of simulations using the CONAN tool.

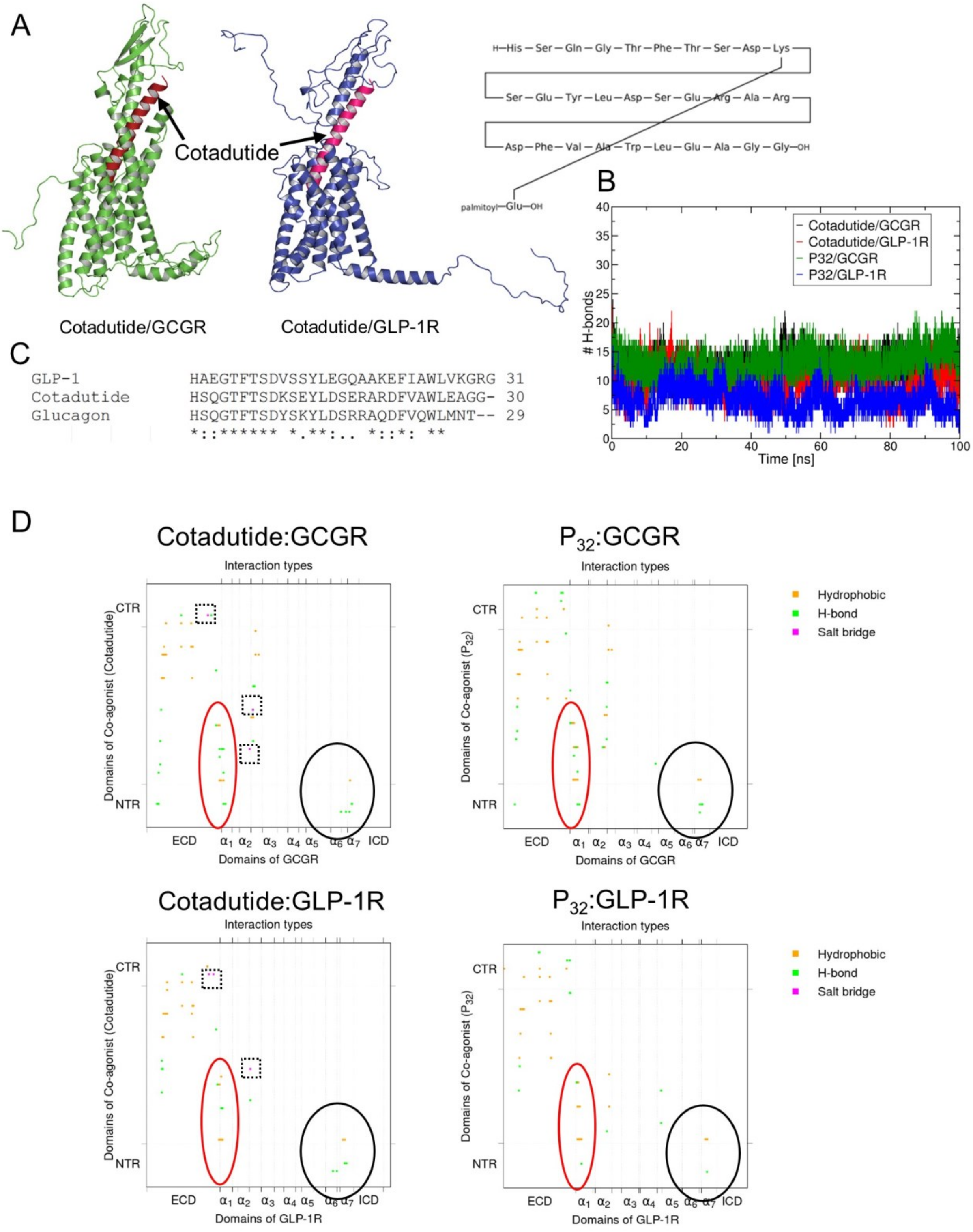

Fig. S21. Modelling of Cotadutide in complex with GRs: (A) Structure of GCGR/GLP-1R in complex with Cotadutide (medi0382), a Dual Receptor Agonist with Glucagon-Like Peptide-1 and Glucagon Activity (under clinical trial), (B) The timelines of H-bonds count show the Cotadutide with GRs along with screened peptide from the phage-displayed library (P_{32}), (C) Sequence alignment between Cotadutide peptide chain and endogenous ligand of GCGR and GLP-1R, (D) Computed interaction maps of Cotadutide/GRs complex compared against interaction maps P_{32}/GRs computed from the

last 50 ns of simulations using the CONAN tool. The red circled area shows the interactions between the NTR of peptides and the hinge region connecting ECD and TMD of receptors, and the black circled area shows the interactions between the NTR of peptides and ECL3 connecting the helix TM6 and TM7. Salt bridge interactions are circled by broken black lines.

**Peptide**	Receptor	1	2	3	4	5	6	7	8	9	10	11	12	13	14	15	**16**	**17**	18	19	**20**	21	22	23	**24**	25	26	**27**	**28**	**29**	**Ligand type**
**Glucagon**	GCGR	H	S	Q	G	T	F	T	S	D	Y	S	K	Y	L	D	S	R	R	A	Q	D	F	V	Q	W	L	M	N	T	**Endogenous**
**P_{11}**	GCGR	H	S	Q	G	T	F	T	S	D	Y	S	K	Y	L	D	S	R	R	A	H	D	F	V	Q	W	L	L	N	T	**PDL-obtained**
**P_{23}**	GCGR	H	S	Q	G	T	F	T	S	D	Y	S	K	Y	L	D	W	R	R	A	Q	D	F	V	Q	W	L	Q	N	T	**PDL-obtained**
**P_{28}**	GCGR	H	S	Q	G	T	F	T	S	D	Y	S	K	Y	L	D	S	R	R	A	Q	D	F	V	D	W	L	I	N	S	**PDL-obtained**
**P_{32}**	GCGR	H	S	Q	G	T	F	T	S	D	Y	S	K	Y	L	D	M	Q	R	A	H	D	F	V	Q	W	L	M	N	T	**PDL-obtained**
**P_{35}**	GCGR	H	S	Q	G	T	F	T	S	D	Y	S	K	Y	L	D	S	R	R	A	Q	D	F	V	Q	W	L	L	D	S	**PDL-obtained**
**MDD_{GCGR}**	GRs	H	S	Q	G	T	F	T	S	D	Y	S	K	Y	L	D	M	R	R	A	Q	D	F	V	D	W	L	M	D	T	**Designed**

Fig. S22. The primary sequence of modelled co-agonist peptides from phage-displayed library[29] (P_{11}, P_{23}, P_{28}, P_{32} and P_{35}), along with template peptide glucagon (endogenous ligand for GCGR); In the sequence of peptides all mutation points are colour-coded in red on glucagon template, and the black ones are the residue belongs to template sequence (GCG). Where residue size corresponds to their importance in terms of binding to GCGR (residue contributed towards GCGR binding with significant ΔG_{bind}).

Peptide	Receptor	1	2	3	4	5	6	7	8	9	10	11	12	13	14	15	**16**	**17**	18	19	**20**	21	22	23	**24**	25	26	**27**	**28**	**29**	Ligand type
**Glucagon**	GCGR	H	S	Q	G	T	F	T	S	D	Y	S	K	Y	L	D	S	R	R	A	Q	D	F	V	Q	W	L	M	N	T	**Endogenous**
**P_{11}**	GLP-1R	H	S	Q	G	T	F	T	S	D	Y	S	K	Y	L	D	S	R	R	A	**H**	D	F	V	Q	W	L	**L**	N	T	**PDL-obtained**
**P_{23}**	GLP-1R	H	S	Q	G	T	F	T	S	D	Y	S	K	Y	L	D	**W**	R	R	A	Q	D	F	V	Q	W	L	**Q**	N	T	**PDL-obtained**
**P_{28}**	GLP-1R	H	S	Q	G	T	F	T	S	D	Y	S	K	Y	L	D	S	R	R	A	Q	D	F	V	**D**	W	L	**I**	N	**S**	**PDL-obtained**
**P_{32}**	GLP-1R	H	S	Q	G	T	F	T	S	D	Y	S	K	Y	L	D	**M**	**Q**	R	A	**H**	D	F	V	Q	W	L	M	N	T	**PDL-obtained**
**P_{35}**	GLP-1R	H	S	Q	G	T	F	T	S	D	Y	S	K	Y	L	D	S	R	R	A	Q	D	F	V	Q	W	L	**L**	**D**	**S**	**PDL-obtained**
**MDD_{GLP-1R}**	GRs	H	S	Q	G	T	F	T	S	D	Y	S	K	Y	L	D	**W**	**Q**	R	A	Q	D	F	V	**D**	W	L	M	**D**	T	**Designed**

Fig. S23. The primary sequence of modelled co-agonist peptides from phage-displayed library[29] (P_{11}, P_{23}, P_{28}, P_{32} and P_{35}), along with template peptide glucagon (endogenous ligand for GCGR); In the sequence of peptides, all mutation points are colour-coded (red and orange) on glucagon template: Orange Residue: residue with lowest repulsive force than the other studied alternative at the same position, and the black ones are the residue belongs to template sequence (GCG). Where residue size corresponds to their importance in terms of binding to GLP-1R (residue contributed towards GLP-1R binding with significant ΔG_{bind}).

Peptide	Receptor	1	2	3	4	5	6	7	8	9	10	11	12	13	14	15	16	17	18	19	20	21	22	23	24	25	26	27	28	29	30	31	Ligand type
Glucagon	GCGR	H	S	Q	G	T	F	T	S	D	Y	S	K	Y	L	D	S	R	R	A	Q	D	F	V	Q	W	L	M	N	T			Endogenous
GLP-1	GLP-1R	H	A	E	G	T	F	T	S	D	V	S	S	Y	L	E	G	Q	A	A	K	E	F	I	A	W	L	V	K	G	R	G	Endogenous
MDD_{GLP-1R}	GRs	H	S	Q	G	T	F	T	S	D	Y	S	K	Y	L	D	W	Q	R	A	Q	D	F	V	D	W	L	M	D	T			Designed
MDD_{GCGR}	GRs	H	S	Q	G	T	F	T	S	D	Y	S	K	Y	L	D	M	R	R	A	Q	D	F	V	D	W	L	M	D	T			Designed
MDD_{GR}	GRs	H	S	E	G	T	F	T	S	D	Y	S	K	Y	L	E	W	Q	R	A	Q	D	F	V	D	W	L	M	D	T	R	G	Designed
Cotadutide	GRs	H	S	Q	G	T	F	T	S	D	K	S	E	Y	L	D	S	E	R	A	R	D	F	V	A	W	L	E	A	G	G		Reference

Fig. S24. The primary sequence of endogenous ligand (GCG and GLP-1), constructed MD-guided co-agonists (MDD_{GCGR}, MDD_{GLP-1R} and MDD_{GR}) based on the per residue-wise decomposition energies data from PDL-peptide/GRs simulation, and reference dual-agonist peptide (Cotadutide); In the sequence of MD-guided peptides all mutation points are colour-coded on glucagon template where red residue: phage-displayed peptide (PDL) residue contributed towards binding with significant ΔG_{bind}, green residue: residue belongs to endogenous ligand (GLP-1), Orange Residue: PDL Residue with lowest repulsive force, and the black ones are the residue belongs to template sequence (GCG). Where residue size (in GLP-1 peptide sequence) corresponds to their importance in terms of binding against the same position residue on GCG peptide with their target receptors, GLP-1R and GCGR, respectively. Colour-coding in blue on the Cotadutide peptide sequence indicates residue mutation on the peptide sequence of glucagon.

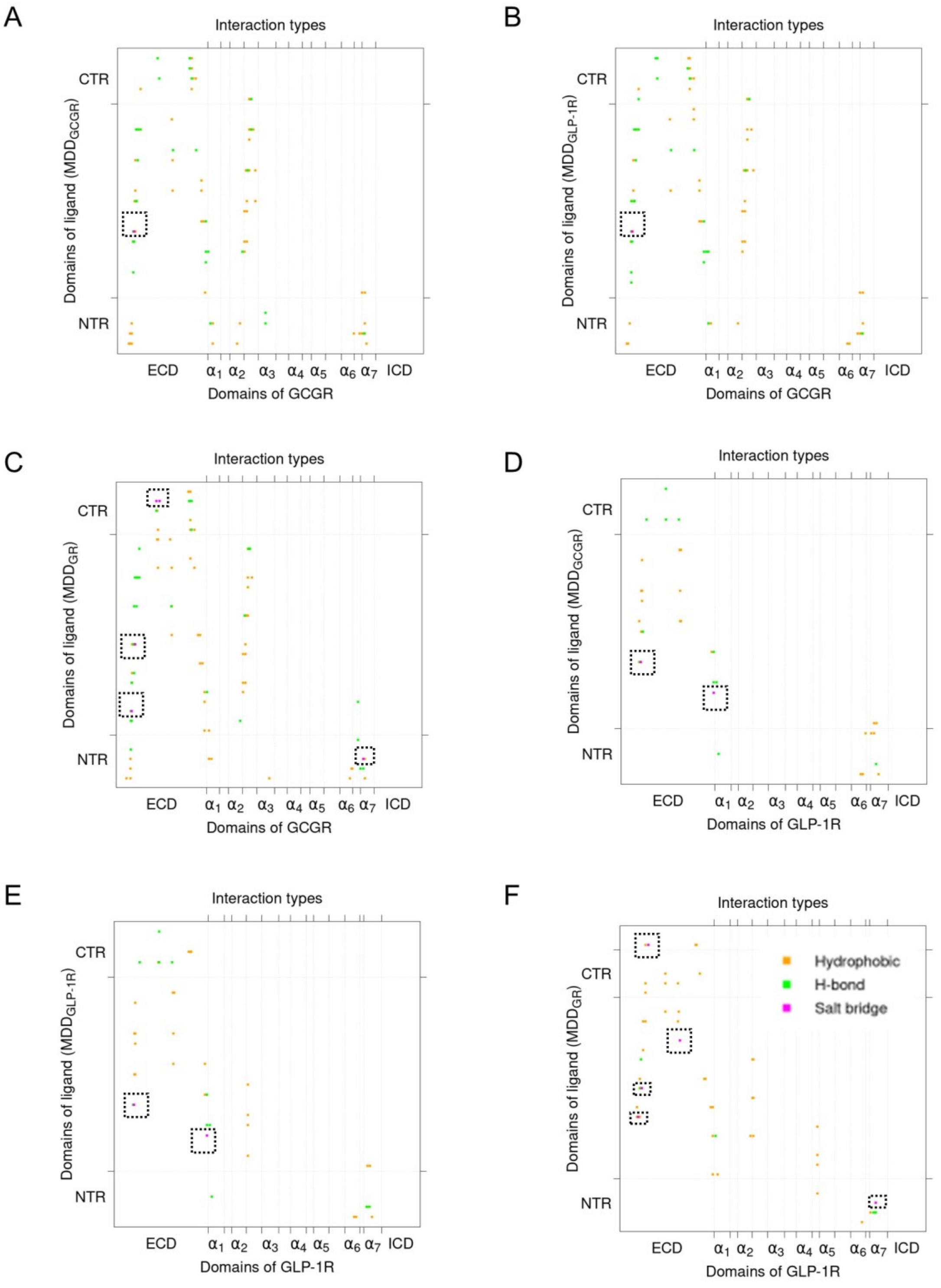

Fig. S25. Computed interaction maps of MDD-peptide/GRs complex computed from the last 50 ns of simulations using the CONAN tool: (A) MDD$_{GCGR}$ co-agonist-bound GCGR, (B) MDD$_{GLP\text{-}1R}$ co-agonist-bound GCGR, (C) MDD$_{GR}$ co-agonist-bound GCGR, (D) MDD$_{GCGR}$ co-agonist-bound GLP-

1R, (E) MDD$_{GLP-1R}$ co-agonist-bound GLP-1R and (F) MDD$_{GR}$ co-agonist-bound GLP-1R. Salt bridge interactions are circled by broken black lines.

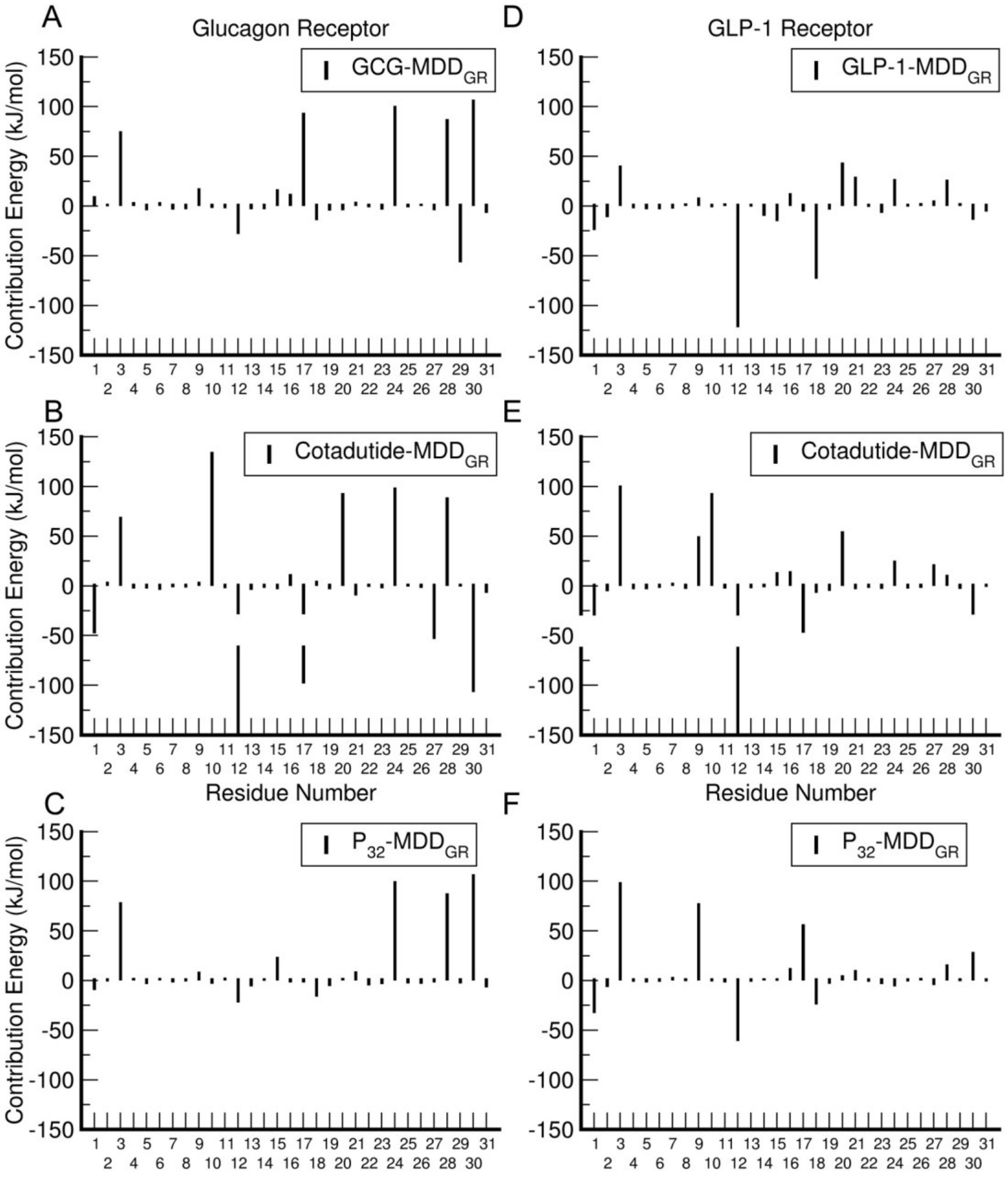

Fig. S26. Difference of residue-wise contribution maps for MDD co-agonist peptides binding to GCGR: (A) GCG relative to MDD$_{GR}$, (B) Cotadutide relative to MDD$_{GR}$, (C) P_{32} relative to MDD$_{GR}$, and MDD co-agonist peptides binding to GLP-1R: (D) GLP-1 relative to MDD$_{GR}$, (E) Cotadutide relative to MDD$_{GR}$, (F) P_{32} relative to MDD$_{GR}$.

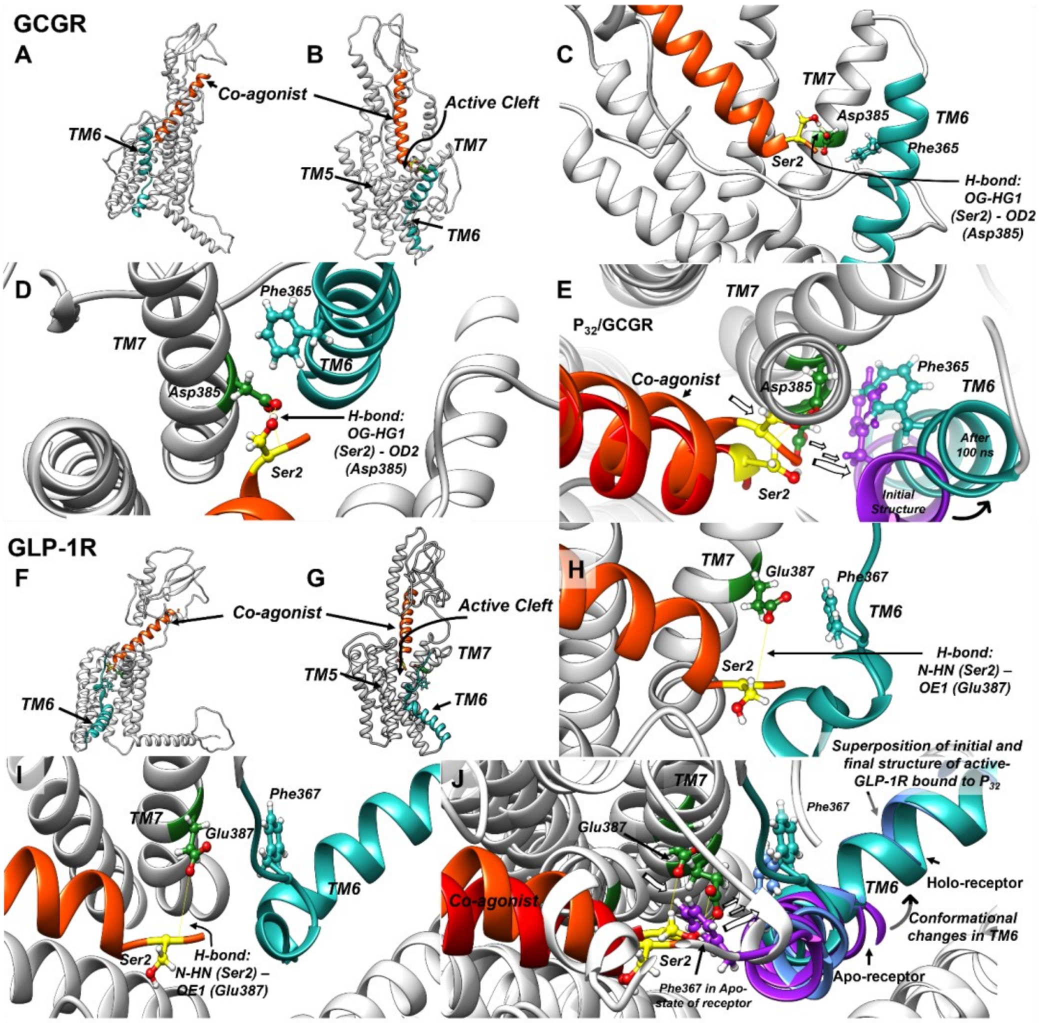

Fig. S27. Structural basis of glucagon and glucagon-like peptide-1 receptor (class B1 GPCRs) binding and activation by designed peptide co-agonist. Conformational dynamics of co-agonist/GCGR: (A) Co-agonist (P_{32}) in complex with GCGR, (B) Side view showing co-agonist P_{32} bound within the cavity of GCGR active cleft, (C) Hydrogen bond between pan-ultimate residue (Ser2) of peptide co-agonist and Asp385$_{TM7}$ of GCGR (Ser2:OG-HG1---OD2:Asp385$_{TM7}$), (D) Representation of hydrogen bond present in the vicinity of Phe365$_{TM6}$, (E) Superimposition of the initial and final structure obtained from MD simulation of P_{32}/GCGR showing a hindrance arises due to hydrogen bond formation causing an outward movement in residue Phe365$_{TM6}$; Conformational dynamics of co-agonist/GLP-1R: (F) Co-agonist (P_{32}) in complex with GLP-1R, (G) Side view showing co-agonist P_{32} bound within the cavity of GLP-1R active cleft, (H) Hydrogen bond between penultimate residue (Ser2) of peptide co-agonist and Glu387$_{TM7}$ of GLP-1R (Ser2:N-HN---OE1:Glu387$_{TM7}$), (I) Representation of hydrogen bond present in the vicinity of Phy367$_{TM6}$, and (J) Superimposition of the initial and final structure obtained from MD simulation of P_{32}/GLP-1R in active-state showing how hindrance arises due to hydrogen bond formation causing an outward movement in residue Phe367$_{TM6}$, the helix TM6 of the initial and final structure of the holo receptor is coloured in blue and cyan, respectively). Superimposed apo-GLP-1R (purple) structure on holo-receptor (P_{32}/GLP-1R) to show the conformational changes in helix 6 upon agonist binding.

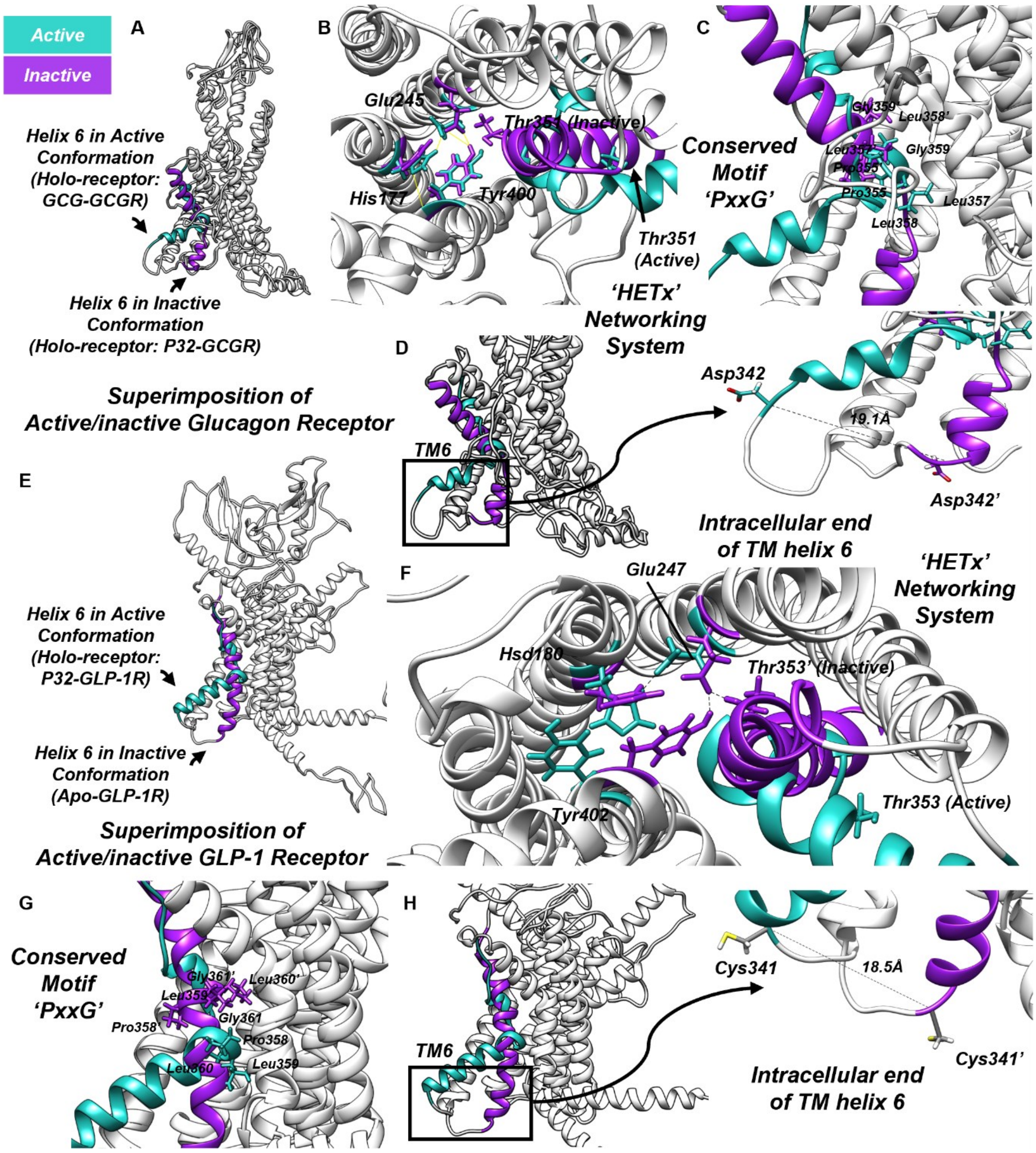

Fig. S28. Activation mechanism of class B1 GPCR: Superimposition of representative conformations of the active and the inactive state of simulated GRs, (A) Conformational transition shown by superimposing the structure obtained from the simulation of two different crystal structures of GCGR in complex with endogenous ligand (glucagon) and designed peptide (P_{32}). (B) HETx network that governs the transition between the inactive and active conformation of GCGR, (C) TM6 helix conformation highlighting the conserved PxxG motif that is important for activation of GCGR, (D) Structural changes in the intracellular end of TM6 helix and measured distance (19.1Å) between the intracellular terminal end of TM6 helix, (E) Conformational transition shown by superimposing the structure obtained from the simulation of GLP-1R in apo-state and in complex with designed peptide (P_{32}), (F) HETx networking system showing the difference in representative inactive and active conformation of GLP-1R, (G) TM6 helix conformation highlighting the conserved PxxG motif that is important for activation of GLP-1R, (H) Structural changes in the intracellular end of TM6 helix and measured distance (18.5Å) between the intracellular terminal end of TM6 helix.

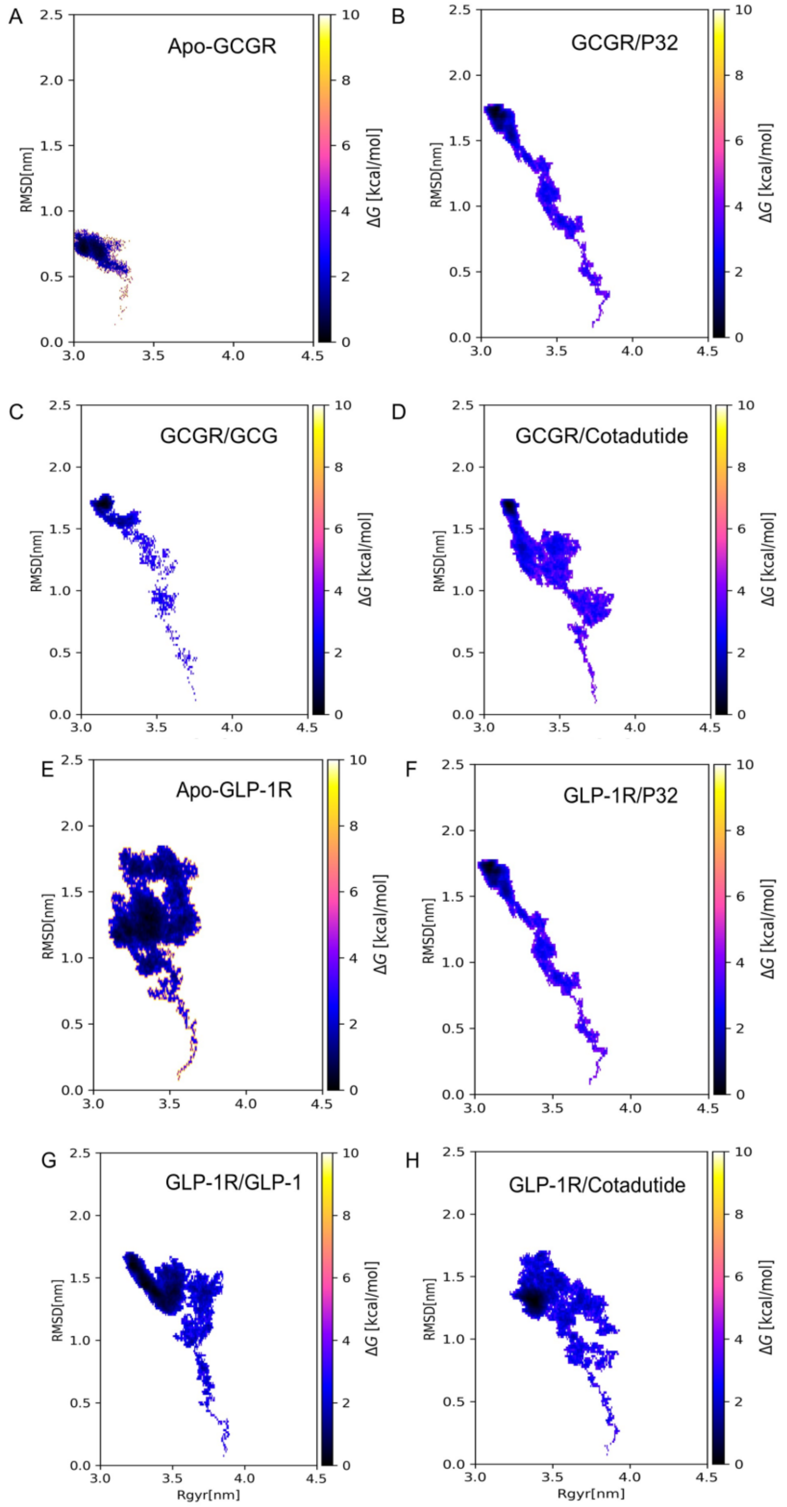

Fig. S29. Conformational landscapes of agonist-bound GRs against apo-GRs with free energy minimum (kcal/mol): free energy maps for simulated agonist in complex with GCGR (A) Apo-GCGR (B) GCGR/P32, (C) GCGR/GCG, (D) GCGR/Cotadutide; and free energy maps for simulated agonist in complex with GLP-1R (E) Apo-GLP-1R, (F) GLP-1R/P32, (G) GLP-1R/GLP-1, (H) GLP-1R/Cotadutide.

Table S1. Description of systems used for MD simulation with corresponding timescales of runs.

Serial Number	System Name	Description	Number of non-water atoms in receptor agonist complex	Length (ns)
	Modelled Endogenous Ligand with GRs			
1	GCGR_GCG	Endogenous peptide agonist (Glucagon) with GCG receptor (initial structure based on crystal structure of partial agonist-bound human GCGR, PDB ID: 5YQZ – Chain A, glucagon receptor and Chain B, peptide)	8091	100
2	GLP-1R_GLP-1	Endogenous peptide agonist (Glucagon-like Peptide-1) with GLP-1 receptor (initial structure based on the crystal structure of agonist-bound human GLP-1R, PDB ID: 6B3J – Chain A, GLP-1 receptor and Chain B, peptide)	7927	100
	Modelled Co-agonist Peptides from Phage-displayed Library with GRs<br>Designed co-agonist peptides (29 residues) in complex with GRs. Here, we used the same initial configuration, replacing the ligand from the initial structure as used above for the simulation of endogenous ligand/GR complexes).			
3	GCGR_P_{11}	Designed PDL peptide agonist (P_{11}) with GCG receptor	8094	100
4	GCGR_P_{23}	Designed PDL peptide agonist (P_{23}) with GCG receptor	8107	100
5	GCGR_P_{28}	Designed PDL peptide agonist (P_{28}) with GCG receptor	8086	100
6	GCGR_P_{32}	Designed PDL peptide agonist (P_{32}) with GCG receptor	8091	100
7	GCGR_P_{35}	Designed PDL peptide agonist (P_{35}) with GCG receptor	8089	100
8	GLP-1R_P_{11}	Designed PDL peptide agonist (P_{11}) with GLP-1 receptor	7937	100
9	GLP-1R_P_{23}	Designed PDL peptide agonist (P_{23}) with GLP-1 receptor	7948	100
10	GLP-1R_P_{28}	Designed PDL peptide agonist (P_{28}) with GLP-1 receptor	7929	100
11	GLP-1R_P_{32}	Designed PDL peptide agonist (P_{32}) with GLP-1 receptor	7934	100
12	GLP-1R_P_{35}	Designed PDL peptide agonist (P_{35}) with GLP-1 receptor	7932	100

	Modelled Dual-agonist Reference Peptide with GRs<br>Modelled reference experimental drug co-agonist peptide (30 residues) in complex with GRs. Here, we used the same initial configuration, replacing the ligand from the initial structure as used above for the simulation of endogenous ligand/GR complexes).			
13	GCGR_Cotadutide	Reference dual-acting peptide agonist (Cotadutide) with GCG receptor	8071	100
14	GLP-1R_Cotadutide	Reference dual-acting peptide agonist (Cotadutide) with GLP-1 receptor	7914	100
	Molecular Dynamics-directed Design (MDD) of Co-agonist Peptides<br>Designed co-agonist peptides in complex with GRs. Here, we used the same initial configuration, replacing the ligand from the initial structure as used above for the simulation of endogenous ligand/GR complexes).			
15	GCGR_MDD$_{GCGR}$	MD-directed (MDD) design peptide (constructed with aided biasedness towards Glucagon receptor with residue-wise decomposition energy data obtained from PDL-peptides and GCG receptor simulation) in complex with GCC receptor<br>Peptide Sequence Length: 29 residues	8091	100
16	GLP-1R_MDD$_{GCGR}$	MD-directed (MDD) design peptide (constructed with aided biasedness towards Glucagon receptor with residue-wise decomposition energy data obtained from PDL-peptides and GCG receptor simulation) in complex with GLP-1 receptor<br>Peptide Sequence Length: 29 residues	7934	100
17	GCGR_MDD$_{GLP-1R}$	MD-directed design peptide (constructed with aided biasedness towards Glucagon-like Peptide-1 receptor with residue-wise decomposition energy data obtained from PDL-peptides and GLP-1 receptor simulation) in complex with GCC receptor<br>Peptide Sequence Length: 29 residues	8091	100
18	GLP-1R_MDD$_{GLP-1R}$	MD-directed design peptide (constructed with aided biasedness towards Glucagon-like Peptide-1 receptor with residue-wise decomposition energy data obtained from PDL-peptides and GLP-1 receptor simulation) in complex with GLP-1 receptor<br>Peptide Sequence Length: 29 residues	7934	100

19	GCGR_MDD$_{GR}$	MD-directed design peptide (constructed with the aim at balanced activity towards both receptor, Glucagon receptor and Glucagon-like Peptide-1 receptor with residue-wise decomposition energy data obtained from PDL-peptides/Endogenous ligands and GCG & GLP-1 receptor simulation) in complex with GCC receptor<br>Peptide Sequence Length: 31 residues	8123	100
20	GLP-1R_MDD$_{GR}$	MD-directed design peptide (constructed with the aim at balanced activity towards both receptor, Glucagon receptor and Glucagon-like Peptide-1 receptor with residue-wise decomposition energy data obtained from PDL-peptides/Endogenous ligands and GCG & GLP-1 receptor simulation) in complex with GLP-1 receptor<br>Peptide Sequence Length: 31 residues	7966	100
	Modelled system to derive the conformational dynamics of GRs:			
21	Apo_GCGR	Apo-glucagon receptor (initial structure based on the crystal structure of partial agonist-bound human GCGR, PDB ID: 5YQZ – Chain A, glucagon receptor)	7619	100
22	Apo_GLP-1R	Apo-GLP-1 receptor (initial structure based on crystal structure of agonist-bound human GLP-1R, PDB ID: 6B3J – Chain A, GLP-1 receptor)	7460	100
23	GCGR_GCG	Endogenous peptide agonist (Glucagon) with GCG receptor (initial structure based on the crystal structure of agonist-bound human GCGR in active state, PDB ID: 6WPW – Chain E, peptide and Chain F, glucagon receptor): the data obtained from this simulation was used to demonstrate conformational dynamics of active GCGR against inactive GCGR (5YQZ).	8091	100

Table S2. MM/PBSA binding free energies (in kJ/mol) of different peptide agonist/GR complexes. Estimation of standard errors was done by using the bootstrap method.

Serial Number	System Name	van der Waals Energy	Electrostatic Energy	Polar Solvation Energy	SASA Energy	Binding Energy (Standard Error)
**1**	GCGR_P_{11}	-764.4 (1.3)	-654.6 (3.8)	1509.0 (3.9)	-91.3 (0.1)	-1.3 (4.4)
**2**	GCGR_P_{23}	-773.7 (1.5)	-489.3 (4.8)	1340.3 (3.8)	-92.4 (0.1)	-15.0 (3.3)
**3**	GCGR_P_{28}	-729.3 (1.3)	-723.6 (3.3)	1345.8 (3.8)	-87.8 (0.1)	-194.9 (2.5)
**4**	GCGR_P_{32}	-715.5 (1.5)	-871.0 (3.3)	1443.9 (4.8)	-88.0 (0.1)	-230.6 (2.4)
**5**	GCGR_P_{35}	-751.7 (1.5)	-886.0 (3.6)	1522.7 (4.8)	-89.3 (0.1)	-204.4 (2.2)
**6**	GCGR_GCG	-803.7 (1.1)	-586.4 (4.3)	1487.2 (5.7)	-91.8 (0.1)	5.9 (2.8)
**7**	GCGR_MDD_{GCGR}	-808.6 (1.6)	-1070.3 (3.8)	1519.1 (3.9)	-92.7 (0.1)	-452.7 (2.7)
**8**	GCGR_$MDD_{GLP\text{-}1R}$	-756.7 (1.4)	-1231.8 (3.9)	1382.4 (5.0)	-89.5 (0.1)	-695.3 (3.3)
**9**	GCGR_MDD_{GR}	-803.8 (1.7)	-1342.1 (5.1)	1661.3 (7.3)	-98.3 (0.1)	-583.0 (3.3)
**10**	GCGR_Cotadutide	-712.9 (1.2)	-1297.5 (4.6)	1539.1 (5.8)	-84.3 (0.1)	-555.6 (3.4)
**11**	GLP-1R_P11	-583.5 (2.4)	-377.2 (3.8)	1149.4 (4.6)	-74.0 (0.1)	114.5 (2.8)
**12**	GLP-1R_P23	-549.4 (2.2)	-339.0 (3.3)	1165.4 (5.5)	-72.2 (0.2)	204.6 (3.2)
**13**	GLP-1R_P28	-428.0 (1.5)	-534.8 (6.4)	1069.3 (9.2)	-58.3 (0.2)	48.1 (2.6)
**14**	GLP-1R_P32	-596.5 (1.5)	-130.1 (3.4)	768.5 (4.2)	-73.0 (0.4)	-31.3 (2.4)
**15**	GLP-1R_P35	-475.6 (1.8)	-169.5 (3.7)	755.7 (4.8)	-58.7 (0.1)	51.8 (2.8)
**16**	GLP-1R_GLP-1	-524.8 (1.5)	-941.9 (7.9)	1169.4 (9.2)	-70.1 (0.1)	-366.5 (3.5)
**17**	GLP-1R_MDDGCGR	-487.6 (1.2)	-426.6 (4.0)	848.6 (4.1)	-58.2 (0.1)	-124.6 (3.1)

**18**	GLP-1R_MDDGLP-1R	-548.3 (1.8)	-450.6 (6.1)	967.9 (7.8)	-67.2 (0.1)	-96.7 (2.8)
**19**	GLP-1R_MDDGR	-591.4 (1.5)	-636.3 (5.3)	1118.2 (6.1)	-77.3 (0.1)	-186.4 (3.5)
**20**	GLP-1R_Cotadutide	-455.4 (1.4)	-1122.6 (5.5)	1517.9 (6.2)	-66.5 (0.1)	-126.5 (3.0)

Table S3. Peptide agonist binding on GCGR and GLP-1R.

Serial Number	Peptide	GCGR Binding Energy (kJ/mol)	GLP-1R Binding Energy (kJ/mol)	GCGR/GLP-1R Binding Energy Ratio	GLP-1R/GCGR Binding Energy Ratio
**1**	P_{11}	-1.3 (4.4)	114.5 (2.8)	-0.01	-88.07
**2**	P_{23}	-15.0 (3.3)	204.6 (3.2)	-0.07	-13.6
**3**	P_{28}	-194.9 (2.5)	48.1 (2.6)	-4.05	-0.24
**4**	P_{32}	-230.6 (2.4)	-31.3 (2.4)	7.36	0.13
**5**	P_{35}	-204.4 (2.2)	51.8 (2.8)	-3.94	0.13
**6**	Cotadutide	-555.6 (3.4)	-126.5 (3.0)	4.39	0.22
**7**	MDD_{GCGR}	-452.7 (2.7)	-124.6 (3.1)	3.63	0.27
**8**	MDD_{GLP_1R}	-695.3 (3.3)	-96.7 (2.8)	7.19	0.13
**9**	MDD_{GR}	-583.0 (3.3)	-186.4 (3.5)	3.12	0.31

Table S4. Contribution of the important peptide residues to the binding of peptide co-agonist to GRs obtained with the MM/PBSA approach. Only residues that contributed significantly (< -50 kJ/mol) towards the ΔG_{bind} are shown.

Co-agonist residues involved in intermolecular interaction with GCGR		Co-agonist residues involved in intermolecular interaction with GLP-1R	
P_{11} Co-agonist Residues	Contribution to ΔG_{bind}	P_{11} Co-agonist Residues	Contribution to ΔG_{bind}
-	-	Asp9	-75.28
P_{23} Co-agonist Residues	Contribution to ΔG_{bind}	P_{23} Co-agonist Residues	Contribution to ΔG_{bind}
Asp9	-84.02	Asp9	-69.29
Asp21	-94.16	-	-
P_{28} Co-agonist Residues	Contribution to ΔG_{bind}	P_{28} Co-agonist Residues	Contribution to ΔG_{bind}
Asp9	-71.92	-	-
Asp21	-102.74	-	-
Asp24	-92.24	-	-
P_{32} Co-agonist Residues	Contribution to ΔG_{bind}	P_{32} Co-agonist Residues	Contribution to ΔG_{bind}
Asp9	-64.32	Asp9	-66.45
Asp21	-93.10	Asp15	-50.60
P_{35} Co-agonist Residues	Contribution to ΔG_{bind}	P_{35} Co-agonist Residues	Contribution to ΔG_{bind}
Asp9	-74.02	-	-
Asp21	-95.77	-	-
Asp28	-106.28	-	-
MDD_{GCGR} Co-agonist Residues	Contribution to ΔG_{bind}	MDD_{GCGR} Co-agonist Residues	Contribution to ΔG_{bind}
Asp9	-75.66	Asp9	-53.96
Asp21	-96.65	Asp15	-63.33
Asp24	-94.72	Asp21	-56.32
Asp28	-110.58	Asp24	-50.82
$MDD_{GLP\text{-}1R}$ Co-agonist Residues	Contribution to ΔG_{bind}	$MDD_{GLP\text{-}1R}$ Co-agonist Residues	Contribution to ΔG_{bind}
Asp9	-95.06	Asp9	-92.15
Asp21	-112.86	Asp15	-64.98
Asp24	-108.37	Asp21	-50.56
Asp28	-123.10	-	-
MDD_{GR} Co-agonist Residues	Contribution to ΔG_{bind}	MDD_{GR} Co-agonist Residues	Contribution to ΔG_{bind}
Glu3	-77.20	Glu3	-99.86
Asp9	-72.14	Asp9	-114.36
Asp21	-101.43	-	-
Asp24	-99.19	-	-
Asp28	-89.69	-	-
GCG Endogenous Peptide Agonist Residues	Contribution to ΔG_{bind}	GLP-1 Endogenous Peptide Agonist Residues	Contribution to ΔG_{bind}
Asp9	-64.04	Glu3	-60.15
Asp21	-82.94	Asp9	-106.81
-	-	Glu15	-59.37
Cotadutide Dual-agonist Peptide Residues	Contribution to ΔG_{bind}	Cotadutide Dual-agonist Peptide Residues	Contribution to ΔG_{bind}
Asp9	-68.84	Asp9	-65.42
Glu12	-76.10	Glu12	-59.03
Glu17	-98.68	-	-
Asp21	-109.88	-	-
Glu27	-66.84	-	-

Table S5. Mutation points on GCG template to design selected PDL-peptide and MDD peptides. Additionally, two residues (Arg30 and Gly31) from the C-terminal region of GLP-1 peptide were adopted into the design of MDD_{GR} to aid the enhanced receptor stabilization.

Peptide	Point A	Point B	Point C	Point D	Point E	Point F
P_{11}	P_{11}^{Q20H}	P_{11}^{M27L}				
P_{23}	P_{23}^{S16W}	P_{23}^{M27Q}				
P_{28}	P_{28}^{Q24D}	P_{23}^{M27I}	P_{28}^{T29S}			
P_{32}	P_{32}^{S16M}	P_{32}^{R17Q}	P_{32}^{Q20H}			
P_{35}	P_{35}^{M27L}	P_{35}^{N28D}	P_{35}^{T29S}			
MDD_{GCGR}	MDD_{GCGR}^{S16M}	MDD_{GCGR}^{Q24D}	MDD_{GCGR}^{N28D}			
$MDD_{GLP\text{-}1R}$	$MDD_{GLP\text{-}1R}^{S16W}$	$MDD_{GLP\text{-}1R}^{R17Q}$	$MDD_{GLP\text{-}1R}^{Q24D}$	$MDD_{GLP\text{-}1R}^{N28D}$		
MDD_{GR}	MDD_{GR}^{Q3E}	MDD_{GR}^{D15E}	MDD_{GR}^{S16W}	MDD_{GR}^{R17Q}	MDD_{GR}^{Q24D}	MDD_{GR}^{N28D}

Table S6. The residue-wise decomposition of ΔG_{bind} (kJ/mol) values of PDL mutants compared to the residue-wise free energies of wild-type endogenous agonists (GCG and GLP-1). All mutation points (yellow) on the GCG template and their wild-type value (orange) are highlighted. Peptide primary sequences of representative mutants are shown in Supplementary Fig. S1.

GCGR							GLP-1R						
**RN**	**GCG**	**P_{11}**	**P_{23}**	**P_{28}**	**P_{32}**	**P_{35}**	**RN**	**GLP-1**	**P_{11}**	**P_{23}**	**P_{28}**	**P_{32}**	**P_{35}**
**1**	150.55	-5.65	143.39	137.36	124.08	129.53	**1**	125.55	139.52	130.75	116.84	145.36	119.55
**2**	2.63	-1.16	0.48	2.85	1.33	0.4	**2**	-5.71	-0.61	-2.5	-1.17	0.52	0.51
**3**	-1.00	-13.03	-10.75	-0.01	0.71	-12.76	**3**	-60.15	0.21	0.75	-1.62	-4.05	0.61
**4**	-4.16	0.16	-2.8	-3.05	-1.67	-2.8	**4**	-0.98	-2.09	1.14	-0.1	0.11	0.16
**5**	-4.45	-1.79	-1.42	-2.12	-3.7	-4.47	**5**	-1.65	-1.67	-0.84	-0.39	-0.43	-0.51
**6**	-23.94	0.1	-23.91	-24.74	-21.88	-21.62	**6**	-20.95	-19.3	-19.23	-18.9	-19.4	-19.38
**7**	-7.52	0.06	-4.13	-6.23	-5.28	-3.53	**7**	-4.38	-1.24	-1.26	-0.34	-1.85	-0.93
**8**	0.09	-0.69	0.51	-1.52	-1.59	-1.45	**8**	0.85	1.48	0.34	-0.33	1.66	0.96
**9**	-64.04	20.88	-84.02	-71.93	-64.32	-74.03	**9**	-106.82	-75.29	-69.29	-37.5	-66.46	-48.51
**10**	-13.31	-2.86	-12.48	-13.1	-15.57	-12.23	**10**	-8.28	-9.55	-12.88	-7.9	-12.06	-9.85
**11**	5.64	-1.32	6.06	4.43	5.76	5.24	**11**	5.02	1	2.13	2.41	3.2	0.75
**12**	63.16	-11.29	88.09	80.45	71.07	61.32	**12**	3.66	58.71	75.07	64.7	74.31	69.31
**13**	-10.06	2.41	-10.7	-8.76	-13.3	-9.23	**13**	-7.87	-9.19	-12.42	-8.35	-7.76	-8.13
**14**	-12.70	0.63	-13.65	-12.64	-10.9	-11.08	**14**	-14.62	-2.95	-4.6	-4.5	-6.02	-0.4
**15**	-0.83	13.29	-14.71	-16.54	-1.66	-16.43	**15**	-59.37	-18.4	-35.16	-44.44	-50.61	-42.45
**16**	-1.79	0.74	-12.01	-2.25	-14.15	-3.51	**16**	-2.67	-2.09	-19.1	-2.86	-13.16	1.48
**17**	88.99	-12.52	88.89	94.17	-2.31	4.48	**17**	-1.81	52.09	35.91	58.23	1.35	3.86
**18**	100.73	-9.53	101.82	107.91	100.01	111.15	**18**	-5.38	49.43	56.87	43.78	67.79	47.88
**19**	-9.97	-1.27	-8.87	-10.25	-10.88	-8.92	**19**	-7.14	-9.51	-8.11	-6.61	-8.47	-8.07
**20**	-3.75	4.44	-1.54	-2.68	1.99	2.93	**20**	42.8	-0.41	5.72	4.66	-0.49	-1.37
**21**	-82.94	9.25	-94.16	-102.75	-93.11	-95.77	**21**	-18.58	-28.75	-29.7	-37.61	-39.91	-17.04
**22**	-26.89	-0.53	-24.52	-25.53	-27.18	-26.71	**22**	-14.26	-14.5	-13.15	-14.57	-14.37	-13.77
**23**	-13.56	0.96	-11.43	-14.12	-13.44	-12.21	**23**	-13.19	-11.38	-9.73	-9.89	-10.19	-11.37
**24**	-0.58	90.33	-1.38	-92.24	-0.02	1.08	**24**	-0.42	-0.44	1.86	-31.42	0.38	-0.68
**25**	-15.41	0.79	-13.87	-14.75	-15.42	-16.28	**25**	-1.7	-4.14	-0.41	-2	-1.67	-2.84
**26**	-16.98	2.19	-15.39	-16.72	-18.26	-18.74	**26**	-10.67	-13.23	-9.6	-10.76	-10.89	-14
**27**	-16.91	-1.52	-11.48	-11.85	-15.38	-13.95	**27**	-5.64	-12.4	0.83	-13.33	-19.01	-15.88
**28**	-2.99	-1.7	-2.04	-1.1	-2.78	-106.29	**28**	10.17	-3.12	1.73	-0.16	1.11	-7.4
**29**	-3.25	1.46	-3.43	-1.67	-5.2	-1.67	**29**	2.71	-0.88	-1.2	1.3	-1.5	-1.2
							**30**	15					
							**31**	-4.03					

Table S7. The effect of *in silico* GCG mutations on GR binding affinities. The residue decomposed ΔG_{bind} (kJ/mol) of GCG mutants that show variation in energies < -5 kJ/mol (green; improves binding), -5 to 5 kJ/mol (orange; no notable change in binding), and > +5 kJ/mol (red; worsens binding), compared to the residue decomposed ΔG_{bind} (kJ/mol) of wild-type endogenous peptides, GCG and GLP-1 (orange) are highlighted. The consequence due to the mutation point is reflected as corresponding positive (improvement in overall binding) or negative (worsening of overall binding) or variable (no notable change in overall binding) effects.

GCGR								GLP-1R							
**RN**	**GCG**	**P_{11}**	**P_{23}**	**P_{28}**	**P_{32}**	**P_{35}**	**Effect**	**RN**	**GLP-1**	**P_{11}**	**P_{23}**	**P_{28}**	**P_{32}**	**P_{35}**	**Effect**
**16**	-1.79	0.74	-12.01	-2.25	-14.15	-3.51	Positive	**16**	-2.67	-2.09	-19.1	-2.86	-13.16	1.48	Positive
**17**	88.99	-12.52	88.89	94.17	-2.31	4.48	Positive	**17**	-1.81	52.09	35.91	58.23	1.35	3.86	Negative
**20**	-3.75	4.44	-1.54	-2.68	1.99	2.93	Negative	**20**	42.8	-0.41	5.72	4.66	-0.49	-1.37	Positive
**24**	-0.58	90.33	-1.38	-92.24	-0.02	1.08	Positive	**24**	-0.42	-0.44	1.86	-31.42	0.38	-0.68	Positive
**27**	-16.91	-1.52	-11.48	-11.85	-15.38	-13.95	Negative	**27**	-5.64	-12.4	0.83	-13.33	-19.01	-15.88	Variable
**28**	-2.99	-1.7	-2.04	-1.1	-2.78	-106.29	Positive	**28**	10.17	-3.12	1.73	-0.16	1.11	-7.4	Positive
**29**	-3.25	1.46	-3.43	-1.67	-5.2	-1.67	Negative	**29**	2.71	-0.88	-1.2	1.3	-1.5	-1.2	Variable

Table S8. Co-agonist-GRs intermolecular hydrogen bonds (H-bonds) from MD simulations. All the listed peptide/receptor residue pairs are involved in intermolecular H-bond formation with at least >80% of frequency. Data from the last 50 ns of the simulation was used.

Peptide/receptor residues involved in intermolecular hydrogen bonds		Peptide/receptor residues involved in intermolecular hydrogen bonds		Peptide/receptor residues involved in intermolecular hydrogen bonds		Peptide/receptor residues involved in intermolecular hydrogen bonds		Peptide/receptor residues involved in intermolecular hydrogen bonds		Peptide/receptor residues involved in intermolecular hydrogen bonds	
GCGR residues	Co-agonist P_{11} residues	GLP-1R residues	Co-agonist P_{11} residues	GCGR residues	Co-agonist P_{23} residues	GLP-1R residues	Co-agonist P_{23} residues	GCGR residues	Co-agonist P_{28} residues	GLP-1R residues	Co-agonist P_{28} residues
GLN20^{ECD}	THR5	THR29^{ECD}	LYS12	GLN20^{ECD}	THR5	ARG20^{ECD}	TYR13	GLN22^{ECD}	GLN3	THR29^{ECD}	LYS12
GLN22^{ECD}	GLN3	THR29^{ECD}	SER16	GLN20^{ECD}	SER8	SER31^{ECD}	ASP15	GLN22^{ECD}	THR7	THR29^{ECD}	SER16
GLN22^{ECD}	THR7	SER31^{ECD}	ASP15	SER25^{ECD}	SER11	TYR69^{ECD}	GLN27	SER25^{ECD}	SER11	ARG121^{ECD}	ASN28
SER25^{ECD}	SER11	SER31^{ECD}	SER16	GLN27^{ECD}	ASP15	ARG121^{ECD}	GLN27	SER25^{ECD}	LYS12	ARG121^{ECD}	SER29
SER25^{ECD}	LYS12	SER117^{ECD}	ASN28	ASP63^{ECD}	THR29	ARG121^{ECD}	THR29	SER25^{ECD}	ASP15	GLU128^{ECD}	GLN20
SER25^{ECD}	ASP15	SER117^{ECD}	THR29	LYS64^{ECD}	THR29	SER124^{ECD}	GLN27	SER25^{ECD}	SER8	SER135^{ECD}	LYS12
SER25^{ECD}	SER8	ARG121^{ECD}	GLN24	GLN113^{ECD}	ASN28	GLU138^{TM1}	TYR13	GLN27^{ECD}	LYS12	SER135^{ECD}	ASP9
GLN27^{ECD}	LYS12	CYS126^{ECD}	GLN24	GLN113^{ECD}	THR29	GLU139^{TM1}	TYR13	GLN27^{ECD}	ASP15	SER136^{ECD}	ASP9
GLN27^{ECD}	ASP15	GLU138^{TM1}	TYR13	ARG116^{ECD}	THR29	TYR148^{TM1}	GLN3	ASP63^{ECD}	SER29	GLU138^{TM1}	TYR13
ASP63^{ECD}	THR29	GLU387^{TM7}	SER2	GLN122^{ECD}	GLN24	TYR148^{TM1}	THR7	LYS64^{ECD}	SER29	GLU138^{TM1}	GLN20
LYS64^{ECD}	THR29	THR391^{TM7}	GLN3	TYR138^{TM1}	ASP9	LYS197^{TM2}	GLN3	GLN113^{ECD}	ASN28	ASP372^{ECL3}	THR5
GLN113^{ECD}	ASN28			GLN142^{TM1}	TYR10	LYS197^{TM2}	THR7	GLN113^{ECD}	SER29	GLU387^{TM7}	SER2
GLN113^{ECD}	THR29			ARG199^{TM2}	TYR10	LYS202^{TM2}	SER11	ARG116^{ECD}	SER29	GLU387^{TM7}	GLN3
GLN122^{ECD}	GLN24			TYR202^{TM2}	SER11	LYS383^{TM7}	SER2	GLN122^{ECD}	ASP24	THR391^{TM7}	GLN3
GLN131^{TM1}	TYR13			TYR202^{TM2}	ASP15	GLU387^{TM7}	SER2	GLN131^{TM1}	TYR13		
LYS132^{TM1}	TYR13			TYR202^{TM2}	ARG18			LYS132^{TM1}	TYR13		
TYR138^{TM1}	ASP9			SER203^{TM2}	ARG18			TYR138^{TM1}	ASP9		
SER139^{TM1}	TYR10			GLN204^{ECL1}	ARG18			SER139^{TM1}	TYR10		
GLN142^{TM1}	TYR10			THR369^{TM6}	SER2			GLN142^{TM1}	TYR10		
TYR145^{TM1}	GLN3			LYS381^{TM7}	SER2			TYR145^{TM1}	GLN3		
TYR202^{TM2}	SER11			ASP385^{TM7}	SER2			TYR145^{TM1}	THR7		
TYR202^{TM2}	ASP15			ASP385^{TM7}	GLN3			TYR149^{TM1}	GLN3		
TYR202^{TM2}	ARG18			SER389^{TM7}	GLN3			LYS187^{TM2}	GLN3		
SER203^{TM2}	ARG18							ARG199^{TM2}	TYR10		
GLN204^{ECL1}	ARG18							TYR202^{TM2}	SER11		
GLU362^{TM6}	GLN3							TYR202^{TM2}	ASP15		
THR369^{TM6}	SER2							TYR202^{TM2}	ARG18		
THR369^{TM6}	THR5							SER203^{TM2}	ARG18		
ASP385^{TM7}	SER2							GLN204^{ECL1}	ARG18		
ASP385^{TM7}	GLN3							ASP385^{TM7}	SER2		
SER389^{TM7}	GLN3										

GCGR residues	Co-agonist P_{32} residues	GLP-1R residues	Co-agonist P_{32} residues	GCGR residues	Co-agonist P_{35} residues	GLP-1R residues	Co-agonist P_{35} residues
GLN22^{ECD}	GLN3	THR29^{ECD}	LYS12	GLN20^{ECD}	THR5	THR29^{ECD}	LYS12
SER25^{ECD}	SER11	SER31^{ECD}	ASP15	GLN22^{ECD}	GLN3	SER31^{ECD}	ASP15
GLN27^{ECD}	LYS12	ASP67^{ECD}	THR29	SER25^{ECD}	LYS12	ASP67^{ECD}	SER29
GLN27^{ECD}	ASP15	GLU68^{ECD}	THR29	SER25^{ECD}	SER8	GLU68^{ECD}	SER29
ASP63^{ECD}	THR29	ARG121^{ECD}	ASN28	GLN27^{ECD}	LYS12	ASP122^{ECD}	GLN24
LYS64^{ECD}	THR29	CYS126^{ECD}	GLN24	GLN27^{ECD}	ASP15	SER124^{ECD}	GLN20
GLN113^{ECD}	ASN28	CYS126^{ECD}	ASN28	ASP63^{ECD}	SER29	SER124^{ECD}	GLN24
GLN113^{ECD}	THR29	GLU127^{ECD}	GLN24	LYS64^{ECD}	SER29	SER135^{ECD}	ASP9
ARG116^{ECD}	THR29	GLU139^{TM1}	TYR13	GLN113^{ECD}	ASP28	GLU138^{TM1}	TYR13
GLN122^{ECD}	GLN24	TYR148^{TM1}	GLN3	GLN113^{ECD}	SER29	GLU139^{TM1}	TYR13
GLN131^{TM1}	TYR13	LYS197^{TM2}	THR7	ARG116^{ECD}	SER29	TYR148^{TM1}	GLN3
GLN131^{TM1}	GLN17	ASN300^{ECL2}	LYS12	GLN122^{ECD}	GLN24	THR391^{TM7}	GLN3
TYR138^{TM1}	ASP9	ASN300^{ECL2}	SER8	GLN131^{TM1}	TYR13		
GLN142^{TM1}	TYR10	GLU387^{TM7}	SER2	GLN131^{TM1}	ARG17		
TYR145^{TM1}	GLN3			TYR138^{TM1}	ASP9		
TYR145^{TM1}	THR7			GLN142^{TM1}	TYR10		
TYR149^{TM1}	GLN3			TYR145^{TM1}	THR7		
ASP195^{TM2}	TYR10			TYR202^{TM2}	SER11		
TYR202^{TM2}	SER11			TYR202^{TM2}	ASP15		
TYR202^{TM2}	ASP15			TYR202^{TM2}	ARG18		
TYR202^{TM2}	ARG18			SER203^{TM2}	ARG18		
GLN204^{ECL1}	ARG18			GLN204^{ECL1}	ARG18		
ASN298^{ECL2}	SER8			THR296^{ECL2}	SER8		
ASP385^{TM7}	SER2			ASN298^{ECL2}	THR5		
ASP385^{TM7}	GLN3			GLU362^{TM6}	GLN3		
SER389^{TM7}	GLN3			ASP385^{TM7}	SER2		
SER390^{TM7}	GLN3			ASP385^{TM7}	GLN3		
				SER389^{TM7}	GLN3		

Table S9. Co-agonist-GRs intermolecular salt bridge during molecular dynamics simulation.

Peptide/receptor residues involved in the intermolecular salt bridge			Peptide/receptor residues involved in the intermolecular salt bridge		
GCGR residues	Co-agonist P_{11} residues	Average contact distance (nm)	GLP-1R residues	Co-agonist P_{11} residues	Average contact distance (nm)
Arg378^{TM7}	Asp9	0.5007	Glu128^{ECD}	Arg17	0.2740
			Glu138^{TM1}	Arg17	0.4531
GCGR residues	Co-agonist P_{35} residues	Average contact distance (nm)	GLP-1R residues	Co-agonist P_{35} residues	Average contact distance (nm)
Arg116^{ECD}	Asp28	0.4905	Arg134^{ECD}	Asp9	0.4332
GCGR residues	Cotadutide residues	Average contact distance (nm)	GLP-1R residues	Cotadutide residues	Average contact distance (nm)
Arg146^{ECD}	Glu27	0.31	Arg151^{ECD}	Glu27	0.34
Asp225$^{\alpha 2}$	Lys10	0.39	Lys160^{ECD}	Glu27	0.27
Arg231^{ECL1}	Asp15	0.42	Lys232^{ECL1}	Asp15	0.27
GLP-1R residues	Co-agonist P_{23} residues	Average contact distance (nm)	GLP-1R residues	Co-agonist P_{28} residues	Average contact distance (nm)
Glu128^{ECD}	Arg17	0.5543	Glu138^{TM1}	Arg17	0.3277
Lys202^{TM2}	Asp15	0.2712			

Table S10. Intrinsic dynamics stability statistics of designed PDL co-agonists, respective wild-type endogenous peptide ligands (GCG & GLP-1), MDD-peptides and the reference dual agonist peptide, Cotadutide. Calculated root means square deviation of peptide backbone of (PDL co-/WT-/MDD co-) agonist in the simulations of (PDL co-/WT-/MDD co-) agonist/GR complex. The reference structure was taken from a 1 ns "isothermal-isobaric" ensemble equilibrium simulation and values within brackets represent the standard deviation. The radius of gyration (Rg) of C-alpha atoms of WT glucagon and glucagon-like peptide-1 receptor in individual co-agonist/GR complex simulations at 298 K.

Peptide Name	Receptor	RMSD	R_g	Receptor	RMSD	R_g
WT	GCGR	0.07 (0.01)	1.28 (0.009)	GLP-1R	0.11 (0.02)	1.39 (0.01)
P_{11}	GCGR	0.08 (0.01)	1.28 (0.01)	GLP-1R	0.15 (0.02)	1.32 (0.01)
P_{23}	GCGR	0.08 (0.01)	1.28 (0.01)	GLP-1R	0.11 (0.02)	1.28 (0.01)
P_{28}	GCGR	0.08 (0.01)	1.27 (0.009)	GLP-1R	0.24 (0.07)	1.28 (0.01)
P_{32}	GCGR	0.1 (0.01)	1.27 (0.009)	GLP-1R	0.12 (0.02)	1.29 (0.01)
P_{35}	GCGR	0.12 (0.02)	1.28 (0.01)	GLP-1R	0.11 (0.02)	1.33 (0.01)

List of Abbreviations and Acronyms

API: Active Pharmaceutical Ingredient
Cryo-EM: Cryo-electron Microscopy
CTR: C-terminal Region
DSSP: Dictionary of Secondary Structure of Proteins
ECD: Extracellular Domain
ECL: Extracellular Loop
FDA: U.S. Food and Drug Administration
FES: Free Energy Surfaces
GCG: Glucagon
GCGR: Glucagon Receptor
GLP-1: Glucagon-like Peptide-1
GLP-1R: Glucagon-like Peptide-1 Receptor
GPCR: G Protein-coupled Receptor
GR or GRs: Collectively both receptors (Glucagon Receptor and Glucagon-like Peptide-1 Receptor)
ICD: Intracellular Domain
ICL: Intracellular Loop
MDD-peptides: Molecular Dynamics (MD)-directed Design peptides
MDFE: Molecular Dynamics Free Energy Simulations
MM/PBSA Approach: Molecular Mechanics combined with the Poisson-Boltzmann Surface Area Approach
NTR: N-terminal Region
OXM: Oxyntomodulin
PDL: Phage-derived Library or Phage-displayed Library (term "PDL-peptides" denotes the five co-agonist peptides (P_{11}, P_{23}, P_{28}, P_{32} and P_{35})[29] screened from Phage-displayed library followed by molecular dynamics simulation)

PK-modifier: Pharmacokinetic-modifier
R_g: Radius of Gyration
RMSD: Root-mean-square Deviation
RMSF: Root Mean Square Fluctuation
TMR: Transmembrane Region
T2DM: Type-2 *diabetes mellitus*
vdW: van der Waals

Supplementary references

1. Best, R. B.; Hummer, G.; Eaton, W. A., Native contacts determine protein folding mechanisms in atomistic simulations. *Proceedings of the National Academy of Sciences* **2013,** *110* (44), 17874-17879.

2. Gilson, M. K.; Davis, M. E.; Luty, B. A.; McCammon, J. A., Computation of electrostatic forces on solvated molecules using the Poisson-Boltzmann equation. *The Journal of Physical Chemistry* **1993,** *97* (14), 3591-3600.

3. Kumari, R.; Kumar, R.; Lynn, A., g_mmpbsa—A GROMACS Tool for High-Throughput MM-PBSA Calculations. *Journal of Chemical Information and Modeling* **2014,** *54* (7), 1951-1962.

4. Genheden, S.; Ryde, U., The MM/PBSA and MM/GBSA methods to estimate ligand-binding affinities. *Expert Opin Drug Discov* **2015,** *10* (5), 449-61.

5. Johnson, B. C.; Métifiot, M.; Pommier, Y.; Hughes, S. H., Molecular Dynamics Approaches Estimate the Binding Energy of HIV-1 Integrase Inhibitors and Correlate with <i>In Vitro</i> Activity. *Antimicrobial Agents and Chemotherapy* **2012,** *56* (1), 411-419.

6. Chodera, J. D.; Mobley, D. L., Entropy-enthalpy compensation: role and ramifications in biomolecular ligand recognition and design. *Annu Rev Biophys* **2013,** *42*, 121-42.

7. Abraham, M. J.; Murtola, T.; Schulz, R.; Páll, S.; Smith, J. C.; Hess, B.; Lindahl, E., GROMACS: High performance molecular simulations through multi-level parallelism from laptops to supercomputers. *SoftwareX* **2015,** *1*, 19-25.

8. Mercadante, D.; Gräter, F.; Daday, C., CONAN: A Tool to Decode Dynamical Information from Molecular Interaction Maps. *Biophys J* **2018,** *114* (6), 1267-1273.

9. Tan, K. P.; Singh, K.; Hazra, A.; Madhusudhan, M. S., Peptide bond planarity constrains hydrogen bond geometry and influences secondary structure conformations. *Curr Res Struct Biol* **2021,** *3*, 1-8.

10. Ferreira de Freitas, R.; Schapira, M., A systematic analysis of atomic protein–ligand interactions in the PDB. *MedChemComm* **2017,** *8* (10), 1970-1981.

11. Unson, C. G.; Wu, C.-R.; Cheung, C. P.; Merrifield, R. B., Positively Charged Residues at Positions 12, 17, and 18 of Glucagon Ensure Maximum Biological Potency *. *Journal of Biological Chemistry* **1998,** *273* (17), 10308-10312.

12. Xiao, Q.; Giguere, J.; Parisien, M.; Jeng, W.; St-Pierre, S. A.; Brubaker, P. L.; Wheeler, M. B., Biological Activities of Glucagon-Like Peptide-1 Analogues in Vitro and in Vivo. *Biochemistry* **2001,** *40* (9), 2860-2869.

13. Pan, C. Q.; Buxton, J. M.; Yung, S. L.; Tom, I.; Yang, L.; Chen, H.; MacDougall, M.; Bell, A.; Claus, T. H.; Clairmont, K. B.; Whelan, J. P., Design of a Long Acting Peptide Functioning as Both a Glucagon-like Peptide-1 Receptor Agonist and a Glucagon Receptor Antagonist *. *Journal of Biological Chemistry* **2006,** *281* (18), 12506-12515.

14. Adelhorst, K.; Hedegaard, B. B.; Knudsen, L. B.; Kirk, O., Structure-activity studies of glucagon-like peptide-1. *J Biol Chem* **1994,** *269* (9), 6275-8.

15. Mroz, P. A.; Perez-Tilve, D.; Mayer, J. P.; DiMarchi, R. D., Stereochemical inversion as a route to improved biophysical properties of therapeutic peptides exemplified by glucagon. *Communications Chemistry* **2019,** *2* (1), 2.

16. Gallwitz, B.; Witt, M.; Paetzold, G.; Morys-Wortmann, C.; Zimmermann, B.; Eckart, K.; Fölsch, U. R.; Schmidt, W. E., Structure/activity characterization of glucagon-like peptide-1. *Eur J Biochem* **1994,** *225* (3), 1151-6.

17. Manandhar, B.; Ahn, J.-M., Glucagon-like Peptide-1 (GLP-1) Analogs: Recent Advances, New Possibilities, and Therapeutic Implications. *Journal of Medicinal Chemistry* **2015,** *58* (3), 1020-1037.

18. Yagami, T., Differential coupling of glucagon and beta-adrenergic receptors with the small and large forms of the stimulatory G protein. *Molecular Pharmacology* **1995,** *48* (5), 849.

19. Graaf, C. d.; Donnelly, D.; Wootten, D.; Lau, J.; Sexton, P. M.; Miller, L. J.; Ahn, J.-M.; Liao, J.; Fletcher, M. M.; Yang, D.; Brown, A. J. H.; Zhou, C.; Deng, J.; Wang, M.-W., Glucagon-Like Peptide-1 and Its Class B G Protein-Coupled Receptors: A Long March to Therapeutic Successes. *Pharmacological reviews* **2016,** *68* (4), 954-1013.

20. Qiao, A.; Han, S.; Li, X.; Li, Z.; Zhao, P.; Dai, A.; Chang, R.; Tai, L.; Tan, Q.; Chu, X.; Ma, L.; Thorsen, T. S.; Reedtz-Runge, S.; Yang, D.; Wang, M.-W.; Sexton, P. M.; Wootten, D.; Sun, F.; Zhao, Q.; Wu, B., Structural basis of Gs and Gi recognition by the human glucagon receptor. *Science* **2020,** *367* (6484), 1346.

21. Zhang, H.; Qiao, A.; Yang, D.; Yang, L.; Dai, A.; de Graaf, C.; Reedtz-Runge, S.; Dharmarajan, V.; Zhang, H.; Han, G. W.; Grant, T. D.; Sierra, R. G.; Weierstall, U.; Nelson, G.; Liu, W.; Wu, Y.; Ma, L.; Cai, X.; Lin, G.; Wu, X.; Geng, Z.; Dong, Y.; Song, G.; Griffin, P. R.; Lau, J.; Cherezov, V.; Yang, H.; Hanson, M. A.; Stevens, R. C.; Zhao, Q.; Jiang, H.; Wang, M. W.; Wu, B., Structure of the full-length glucagon class B G-protein-coupled receptor. *Nature* **2017,** *546* (7657), 259-264.

22. Liang, Y.-L.; Khoshouei, M.; Glukhova, A.; Furness, S. G. B.; Zhao, P.; Clydesdale, L.; Koole, C.; Truong, T. T.; Thal, D. M.; Lei, S.; Radjainia, M.; Danev, R.; Baumeister, W.; Wang, M.-W.; Miller, L. J.; Christopoulos, A.; Sexton, P. M.; Wootten, D., Phase-plate cryo-EM structure of a biased agonist-bound human GLP-1 receptor–Gs complex. *Nature* **2018,** *555* (7694), 121-125.

23. Hilger, D.; Kumar, K. K.; Hu, H.; Pedersen, M. F.; O'Brien, E. S.; Giehm, L.; Jennings, C.; Eskici, G.; Inoue, A.; Lerch, M.; Mathiesen, J. M.; Skiniotis, G.; Kobilka, B. K., Structural insights into differences in G protein activation by family A and family B GPCRs. *Science* **2020,** *369* (6503), eaba3373.

24. Trzaskowski, B.; Latek, D.; Yuan, S.; Ghoshdastider, U.; Debinski, A.; Filipek, S., Action of molecular switches in GPCRs--theoretical and experimental studies. *Current medicinal chemistry* **2012,** *19* (8), 1090-1109.

25. Song, G.; Yang, D.; Wang, Y.; de Graaf, C.; Zhou, Q.; Jiang, S.; Liu, K.; Cai, X.; Dai, A.; Lin, G.; Liu, D.; Wu, F.; Wu, Y.; Zhao, S.; Ye, L.; Han, G. W.; Lau, J.; Wu, B.; Hanson, M. A.; Liu, Z. J.; Wang, M. W.; Stevens, R. C., Human GLP-1 receptor transmembrane domain structure in complex with allosteric modulators. *Nature* **2017,** *546* (7657), 312-315.

26. Jazayeri, A.; Rappas, M.; Brown, A. J. H.; Kean, J.; Errey, J. C.; Robertson, N. J.; Fiez-Vandal, C.; Andrews, S. P.; Congreve, M.; Bortolato, A.; Mason, J. S.; Baig, A. H.; Teobald, I.; Doré, A. S.; Weir, M.; Cooke, R. M.; Marshall, F. H., Crystal structure of the GLP-1 receptor bound to a peptide agonist. *Nature* **2017,** *546* (7657), 254-258.

27. Gargaro, A. R.; Bloomberg, G. B.; Dempsey, C. E.; Murray, M.; Tanner, M. J., The solution structures of the first and second transmembrane-spanning segments of band 3. *Eur J Biochem* **1994,** *221* (1), 445-54.

28. Hilger, D.; Kumar, K. K.; Hu, H.; Pedersen, M. F.; Giehm, L.; Mathiesen, J. M.; Skiniotis, G.; Kobilka, B. K., Structural insights into ligand efficacy and activation of the glucagon receptor. *bioRxiv* **2019**, 660837.

29. Demartis, A.; Lahm, A.; Tomei, L.; Beghetto, E.; Di Biasio, V.; Orvieto, F.; Frattolillo, F.; Carrington, P. E.; Mumick, S.; Hawes, B.; Bianchi, E.; Palani, A.; Pessi, A., Polypharmacy through Phage Display: Selection of Glucagon and GLP-1 Receptor Co-agonists from a Phage-Displayed Peptide Library. *Scientific Reports* **2018,** *8* (1), 585.
